# Engineered dual selection for directed evolution of SpCas9 PAM specificity

**DOI:** 10.1038/s41467-020-20650-x

**Published:** 2021-01-13

**Authors:** Gregory W. Goldberg, Jeffrey M. Spencer, David O. Giganti, Brendan R. Camellato, Neta Agmon, David M. Ichikawa, Jef D. Boeke, Marcus B. Noyes

**Affiliations:** 1 Institute for Systems Genetics, NYU Langone Health, New York, NY 10016 USA; 2 Department of Biomedical Engineering, NYU Tandon School of Engineering, Brooklyn, NY 11201 USA; 3 Present Address: Neochromosome, Inc., Alexandria Center for Life Science, New York, NY 10016 USA

**Keywords:** Synthetic biology, Experimental evolution, CRISPR-Cas9 genome editing

## Abstract

The widely used *Streptococcus pyogenes* Cas9 (SpCas9) nuclease derives its DNA targeting specificity from protein-DNA contacts with protospacer adjacent motif (PAM) sequences, in addition to base-pairing interactions between its guide RNA and target DNA. Previous reports have established that the PAM specificity of SpCas9 can be altered via positive selection procedures for directed evolution or other protein engineering strategies. Here we exploit in vivo directed evolution systems that incorporate simultaneous positive and negative selection to evolve SpCas9 variants with commensurate or improved activity on NAG PAMs relative to wild type and reduced activity on NGG PAMs, particularly YGG PAMs. We also show that the PAM preferences of available evolutionary intermediates effectively determine whether similar counterselection PAMs elicit different selection stringencies, and demonstrate that negative selection can be specifically increased in a yeast selection system through the fusion of compensatory zinc fingers to SpCas9.

## Abstract (web summary)

The PAM specificity of SpCas9 can be altered with positive selection during directed evolution. Here the authors use simultaneous positive and negative selection to improve activity on NAG PAMs while reducing activity on NGG PAMs.

## Introduction

Derived from the CRISPR-Cas systems of prokaryotes^1^, CRISPR-associated (Cas) proteins operate in complex with their RNA guides to license sequence-specific nucleic acid targeting. The sequence specificity of Cas nucleases that are commonly used for DNA editing, including *Streptococcus pyogenes* Cas9 (SpCas9, hereafter Cas9), is jointly mediated by RNA–DNA base pairing and essential interactions with a target-abutting DNA sequence known as a protospacer adjacent motif (PAM)^2,3^. Cas9’s PAMs are recognized through hardwired protein–DNA interactions that mechanistically precede the formation of guide-RNA-dependent R-loops^4^, and therefore limit the sequence space available for targeting. Wild-type (Wt) Cas9 is known to interact with NGG PAMs and to a lesser extent with NAG and NGA PAMs, but not NCC PAMs^5–8^. The use of natural Cas9 orthologs or alternative Cas nucleases with different PAM repertoires has allowed additional targets to be accessed, albeit with varied degrees of success^9–13^. Other efforts to circumvent this limitation have employed protein engineering strategies that alter or broaden the PAM specificity of Cas9^7,14–18^, such as directed evolution systems that incorporate positive selection. Whereas Cas9 variants with broadened PAM repertoires offer expanded targeting scope, variants with altered PAM specificity offer alternative sequence requirements that can be exploited for single-nucleotide allelic discrimination at the PAM-recognition step^19–21^.

Directed evolution has been effectively exploited to re-engineer the substrate specificity of proteins from a diverse range of families^22,23^. Specificities can be evolved using positive selection, wherein the protein of interest’s activity on a substrate has been coupled with a survival advantage under the engineered screening conditions^24^. Positive selection is sufficient to drive the evolution of generalist variants with relaxed specificities, as well as variants with truly altered, orthogonal specificities^22,25^. To facilitate the evolution of orthogonal specificities in particular, directed evolution procedures may incorporate negative selection pressures that select against variants which retain their parental substrate preferences^26–31^. Simultaneous positive and negative selection has been previously employed to evolve high-fidelity Cas9 variants with reduced off-target activity^32,33^, but has not been utilized for the evolution of PAM specificity.

In the present study, we engineer *Escherichia coli* and *Saccharomyces cerevisiae* reporter systems to generate PAM-dependent, simultaneous positive and negative selection (dual selection) in vivo. We use these dual-selection systems to obtain Cas9 variants from two rounds of directed evolution and characterize their altered PAM preferences in downstream assays. Two ostensibly similar negative-selection PAMs were found to differentially counterselect the intermediate variant from round 1, due to its slight preference for one of the PAMs. Ultimately, we map the substitutions onto a published structure of PAM-bound Cas9 and propose a molecular model that may account for the functional alterations in PAM preference evolved in each round.

## Results

### Establishment of an ω-dCas9 dual-selection system in *E. coli*

**Fig. 1:**
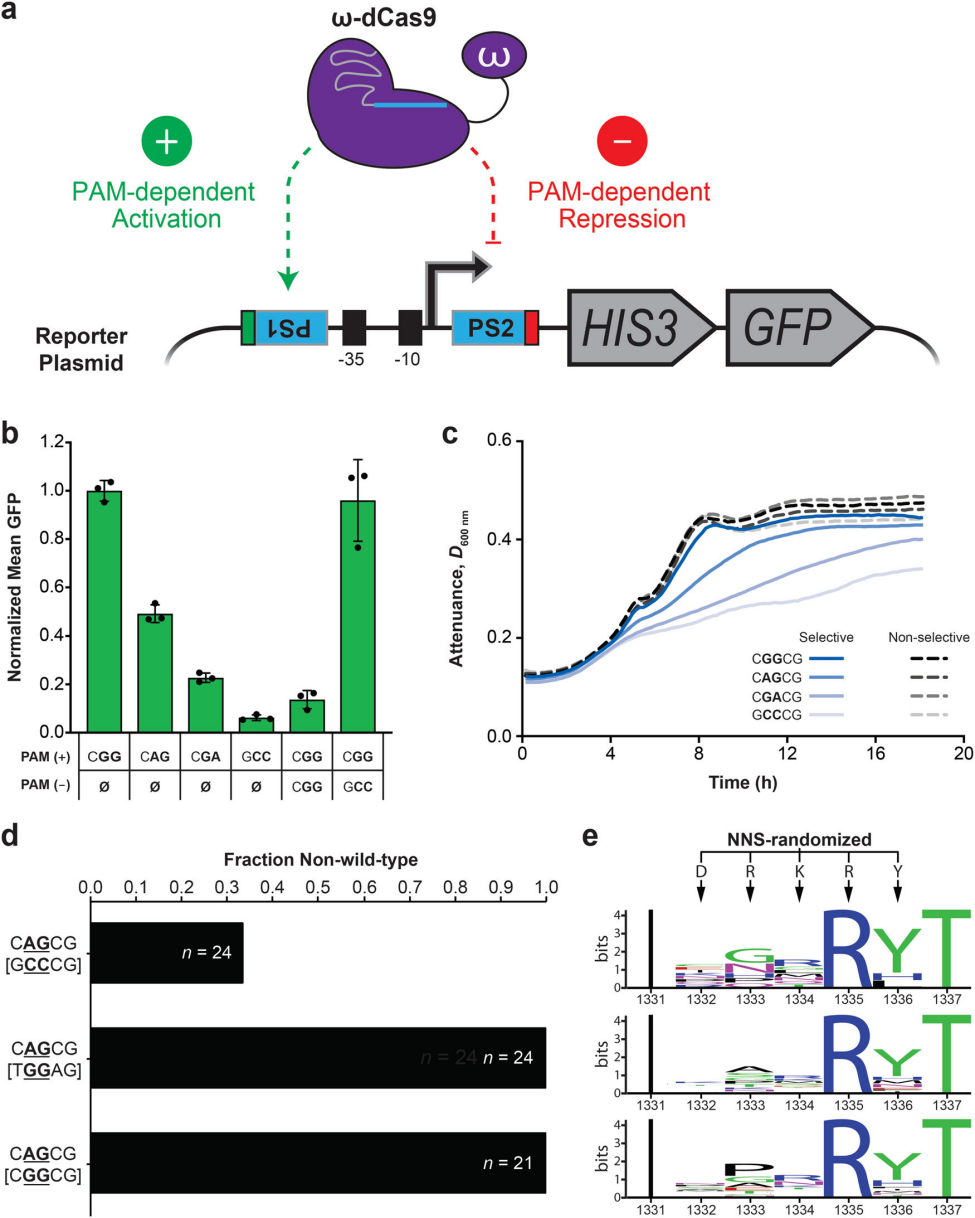
B1H-based dual-selection system for directed evolution of Cas9’s PAM specificity. **a** Schematic summary of the ω-dCas9 dual-selection system. Expression of the reporter plasmid’s synthetic *HIS3/GFP* operon is driven by transcription from an upstream *lac* core promoter (−10 and −35 boxes). ω-dCas9’s PAM-dependent interactions with two identical protospacers (PS1 and PS2) upstream and downstream of the promoter license activation and dominant repression, respectively. PAM positioning adjacent to each protospacer is indicated in green or red. **b** GFP fluorescence measured from *E. coli* cells with Wt ω-dCas9 and different reporter plasmid configurations. PAM sequence at the upstream (+) or downstream (−) protospacer is indicated; Ø denotes absence of the protospacer. Normalized mean values were calculated from populations of single cells analyzed by flow cytometry. Error bars, mean ± s.d. (*n* = 3, biological replicates). **c** High-resolution growth monitoring of Wt ω-dCas9 cultures with the single-target reporter plasmids tested in panel **b**. Minimal media were supplemented with 8 mM 3-AT (selective) or 0.1% histidine (non-selective). Attenuance (*D*_600 nm_) measurements plotted at each time point represent the average from three biological replicate cultures grown in parallel. **d** Bar plot representing the fraction of non-Wt PID sequences recovered from three selection experiments with the indicated reporter plasmid PAMs (the counterselection PAM is bracketed). The total number, *n*, of complete sequences recovered in each experiment is noted on the bars. Recoded Wt sequences were not detected in these experiments. **e** WebLogo^37^ summaries of NNS-randomized residues identified in non-Wt sequences from the experiments in panel **d**. Only eight (non-Wt) sequences were included for the top plot. Source data are available in the Source Data file.

Our initial efforts to evolve Cas9 variants with altered PAM preferences aimed to leverage *E. coli’s* formidable transformation efficiency, as well as previously described bacterial one-hybrid (B1H) reagents with a low background of false positives^34^. The catalytically dead double mutant of Cas9, Cas9^D10A,H840A^ (dCas9), was previously shown to offer programmable activation and repression in *E. coli* when fused to the non-essential omega (ω) subunit of *E. coli*’s RNA polymerase^35^. We exploited these features to establish a plasmid-based dual-selection system that adapts B1H reagents for use with ω-dCas9 fusions. In this system, ω-dependent transcriptional activation of a plasmid-borne *HIS3* marker is required for growth of the auxotrophic selection host within minimal media that lacks histidine. Histidine auxotrophy thus provides positive selection for functional B1H interactions between a binding site upstream of the *HIS3* promoter and ω fusion proteins expressed in *trans* (see ref. ^36^ for a review). To engineer dual selection, we programmed ω-dCas9 with a single guide RNA (sgRNA) that matches two identical protospacer sequences positioned upstream and downstream of the *HIS3* promoter in our reporter plasmids (Fig. 1a). This heterologous protospacer sequence is derived from a human-codon-optimized EGFP (*hEGFP*) expression cassette. Activation by ω-dCas9 at the upstream target is required to overcome positive selection pressure, whereas binding at the downstream target is designed to impair growth via dominant repression, thereby generating simultaneous negative selection. A *GFP* marker transcriptionally fused downstream of the *HIS3* coding sequence and not recognized by the *hEGFP* spacer provides an alternative readout of promoter activity. ω-dCas9’s binding interactions at each target are intrinsically PAM-dependent^4^; by modulating only the PAM sequences of these targets, we reasoned that this system could be employed for directed evolution of Cas9’s PAM specificity.

Previous reports demonstrated that ω-dCas9 (or dCas9-ω) transcriptional activation requires precise target site positioning upstream of promoters^15,35^, but prescriptive guidelines for ensuring optimal activation were proposed only recently^38^. Therefore, in our pilot experiments with Wt ω-dCas9, we sampled a series of 20 reporter plasmids that each carries a single upstream protospacer abutting an NGG PAM at different positions and orientations with respect to the promoter’s -35 box (Supplementary Fig. 1a, b). We found that maximal *GFP* activation was achieved with a ‘Reverse’ target that positions the PAM 25 or 36 base pairs (bp) upstream from the -35 box, and used the ‘R25’ configuration (Supplementary Fig. 1b) in subsequent designs. In additional pilot experiments, we confirmed that NGG-dependent *GFP* activation could be strongly repressed by inserting a second identical protospacer with another NGG PAM downstream of the promoter (Supplementary Fig. 1c). Equivalent repression was achieved with downstream targets in either orientation, presumably due to their proximity to the promoter’s transcriptional start site^35,39^, so we proceeded with the ‘Forward’ orientation for downstream targets (Supplementary Fig. 1b) in all subsequent experiments. Using single-target reporters, we further confirmed that *GFP* activation is hierarchically impaired when suboptimal PAMs are installed upstream (Fig. 1b), consistent with Wt Cas9’s known PAM preferences. To determine whether this hierarchy of activation would also detectably impact *HIS3* expression from these reporters and thus affect growth in selective minimal media, we monitored growth in liquid culture over 24 h after subculturing from rich media into either selective or non-selective minimal media (Fig. 1c). The selective minimal media used in B1H experiments throughout this work was supplemented with 3-aminotriazole (3-AT), a competitive inhibitor of the *HIS3* gene product that reduces background growth and effectively increases the stringency of our positive selection. As expected, growth was impaired in selective minimal media when suboptimal PAMs were provided, whereas growth in non-selective minimal media containing histidine and lacking 3-AT was PAM-independent (Fig. 1c).

We next tested our dual-selection system in library screening experiments intended to evolve ω-dCas9 variants that discriminate between similar PAM sequences. The initial ω-dCas9 library we constructed and screened was NNS-randomized (where ‘N’ represents any of the four DNA bases and ‘S’ represents ‘C’ or ‘G’) at five codons (1332–1336) of the PAM-interacting domain (PID), including the R1333 and R1335 residues that are critical for Cas9’s activity on NGG PAMs^40^. NNS-randomized triplets offer at least one codon for each of the 20 standard amino acids, and one stop codon. We configured three reporters with a CAG PAM for positive selection and one of either GCC, TGG, or CGG counterselection PAMs that had been validated in preliminary work above (Fig. 1b; Supplementary Fig. 1c). We considered the first five positions of the PAM when designing our experiments (Fig. 1d) because base-specific interactions outside of positions 2 and 3 have been found to contribute to the PAM preferences of engineered PID variants^41,42^, and even Wt Cas9^5,7^. Previous efforts to improve activity on NAG PAMs using positive selection converged on mutations outside the PID and reported no reduction in NGG recognition^43^, so we endeavored to evolve preference for NAG PAMs over NGG PAMs using our dual-selection system and PID mutagenesis. After co-delivering the library and reporter plasmids to our B1H selection strain and plating on selective media with 8 mM 3-AT, colonies were sampled at random for Sanger sequencing of the PID region. Exclusively non-Wt PID sequences were recovered in experiments with the NGG counterselection PAMs, whereas Wt Cas9 sequences encoding ‘DRKRY’ at positions 1332–1336 were multiply represented in experiments with the NCC control PAM (Fig. 1d; Supplementary Table 1). All of these Wt DNA sequences were identical to that of the parent template used for mutagenesis, did not conform to our NNS randomization scheme, and resulted from template-derived carryover during library construction. Parental carryover was avoided in subsequent plasmid library builds (see Methods). Importantly, an arginine was recovered at position 1335 in every isolate, even when non-parental, NNS-encoded library members were considered (Fig. 1e; Supplementary Table 1). Selection for an arginine at this position is expected to preserve a critical major-groove contact with the guanine at position three of the NAG PAM we employed for positive selection^40^. Collectively, these results immediately served to validate the fidelity of our selection strategy. However, the mutant isolates we obtained in these experiments were all unique, suggesting that they possessed equivalent functional activities, or that our reporter system was not stringent enough to converge on particular variants with the strongest functional outputs. Similar results were obtained in selection experiments with 5 mM 3-AT and the same NGG-counterselecting dual reporters, where no single variant was represented more than twice (Supplementary Table 2). In an effort to achieve greater selection stringency, we next screened this PID library with a cleavage-based selection system that employs *S. cerevisiae* reporter strains described below.

### *S. cerevisiae* selections yield a round 1 variant that prefers NAG over NGG PAMs

**Fig. 2:**
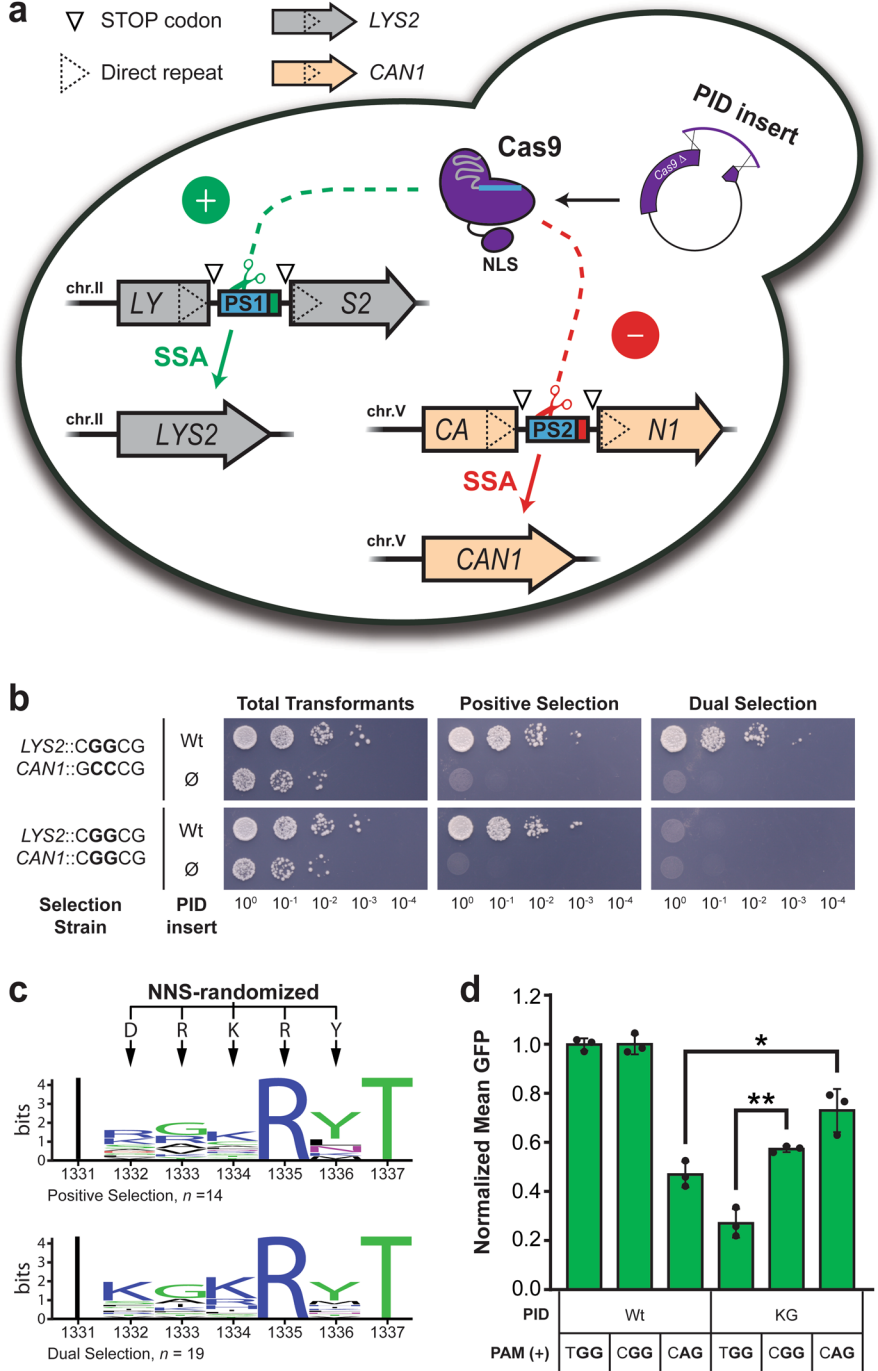
Cleavage-dependent selections in *S. cerevisiae* converge on a doubly substituted PID variant with altered PAM preference. **a** Schematic summary of the single-strand annealing (SSA) reporters employed for dual selection in yeast. The endogenous *LYS2* and *CAN1* marker loci are disrupted with identical protospacer insertions (PS1 or PS2, with PAMs in green or red) flanked by stop codons and ~100 bp direct repeat sequences which are not naturally duplicated in their respective native loci. Transformation-associated assembly of the Cas9 backbone with a complementing PID fragment allows for cleavage-dependent reconstitution of one or both markers through the SSA pathway. A single SV40 nuclear localization signal (NLS) is fused to the C-terminus of Cas9. **b** Validation of the yeast system using two selection strains configured with CGG or GCC PAMs at their *LYS2* and *CAN1* loci as indicated. Strains were transformed with backbone DNA and either a Wt PID insert or no PID (Ø), and plated in parallel as 10-fold serial dilutions on positive- and dual-selection media, as well as control media to enumerate total transformants. **c** WebLogo^37^ summaries of NNS-randomized residues identified in non-Wt isolates from the positive-selection (top) or dual-selection (bottom) screening experiments. The number of isolates used to generate each plot is denoted below. Recoded Wt isolates were not detected in either of these experiments. **d** GFP activation assays comparing the KG variant with Wt, normalized as in Fig. 1b. PAMs present at the upstream protospacer in single-target reporter plasmids are indicated below. Error bars, mean ± s.d. (*n* = 3, biological replicates). *p = 0.0180; **p = 0.0099. Source data are available in the Source Data file.

To expand our efforts beyond the binding-dependent selections in *E. coli*, we constructed a cleavage-dependent dual-selection system for screening PID libraries in yeast. This system exploits homology-directed library assembly in vivo^44,45^ and single-strand annealing^46,47^ (SSA) cleavage reporters. We adapted this approach from methods employed in a previous study, wherein an auxotrophic positive selection and colorimetric counterscreen were used to select a high-fidelity Cas9 variant from libraries mutagenized in the REC3 domain^48^. Our modified approach employs a *LYS2* positive-selection marker and a *CAN1* negative-selection marker for canavanine-based counterselection (Fig. 2a). Each locus is disrupted with identical protospacer insertions and a direct repeat which, upon cleavage, allows markers to be efficiently reconstituted through the SSA repair pathway. We used the same *hEGFP* protospacer from our B1H system, and again varied only the PAM sequences at each target site. In addition, our *Cas9* assembly strategy was designed to facilitate mutagenesis of the PID rather than the REC3 domain. We validated this system by co-transforming two dual-selection strains with the Wt PID insert and backbone fragments for in vivo assembly. Owing to the strong ‘CGG’ PAM installed at the *LYS2* locus in each strain, transformants efficiently survived positive selection on media lacking lysine—but only when inserts were provided (Fig. 2b). Moreover, when the same culture dilutions were plated on dual-selection media supplemented with canavanine, efficient survival was only observed for the strain that harbored a negligible GCC PAM at the *CAN1* locus.

We next used this yeast selection system to screen PID variants derived from the NNS-randomized ω-dCas9 library constructed above. To emulate one of our B1H reporter plasmids (Fig. 1d), we designed a selection strain with CAG at the *LYS2* target and CGG at the *CAN1* target. PID fragments were amplified from the library using 12 cycles of PCR to help reduce bias, and assembled with a 10-fold excess of backbone to minimize the frequency of double library transformants (see Methods). In accordance with our ω-dCas9 results (Supplementary Tables 1 and 2), parental Wt sequences were recovered under positive-selection conditions but not recovered with dual selection (Supplementary Table 3). However, when non-parental sequences were examined (Fig. 2c; Supplementary Table 3), one NNS-encoded variant was multiply represented in positive-selection and dual-selection experiments (3/14 isolates and 7/19 isolates, respectively; Supplementary Table 3). This doubly substituted variant (D1332K/R1333G, hereafter, ‘KG’), upon re-testing in single-target *LYS2* selection strains, was strongly reduced in its cleavage activity on the CGG PAM relative to Wt but not reduced in its activity on the CAG PAM (Supplementary Fig. 2a). Consistent with its apparently altered PAM preference, the KG variant was previously detected in one of the ω-dCas9 dual-selection experiments where Wt was robustly counterselected in *E. coli* (Supplementary Table 2). We further validated this variant by isolating its ω-dCas9 expression plasmid and pairing it with single-target reporters for testing in activation and repression assays. Whereas activation with either of the NGG PAMs was reduced relative to Wt, activation with the CAG PAM was increased (Fig. 2d). We corroborated this result with a repression assay where a single target was installed downstream of a constitutive promoter. In this context, repression with the CGG PAM was reduced relative to Wt, whereas repression with the CAG PAM was increased (Supplementary Fig. 2b).

Interestingly, while testing the KG variant in ω-dCas9 activation assays, we observed significantly stronger activation with the CGGCG PAM relative to the TGGAG PAM (Fig. 2d) despite having isolated this variant with counterselection against the CGG PAM in yeast. This prompted us to further investigate how our choice of negative-selection PAMs was influencing our selections in *E. coli*. As noted above, we considered a 5 bp PAM sequence in our experiments because interactions beyond the canonical 3 bp PAM have been previously described for Wt and engineered PID variants^5,7,41,42^. We first assessed the KG variant’s ability to bypass each NGG counterselection PAM in clonal, B1H-dependent growth assays. After co-transforming the B1H selection strain with our reporter and KG expression plasmids, cells were plated on selective minimal media with three different concentrations of 3-AT and monitored over time. Whereas the TGG counterselection PAM had virtually no effect on plating efficiency assessed at 44 h for any 3-AT concentration, the CGG PAM strongly inhibited colony formation at 8 mM 3-AT, and to a lesser extent at 5 mM 3-AT, even after 44 h incubations (Supplementary Fig. 3a, b). To more definitively demonstrate that this effect can operate in our library selection experiments, we deep-sequenced pools of co-transformants selected on plates with 2 mM or 8 mM 3-AT, using the same reporter plasmids from Fig. 1d. When considering all of the PID-containing reads retrieved after selection with the control reporter, parental Wt sequences comprised about 60% at either 3-AT concentration (Supplementary Fig. 3c), in agreement with our quantification from colony sequencing (Fig. 1d; Supplementary Table 1). In dual-selection experiments, this frequency dropped to less than 1 in 10,000. When we considered only reads with NNS-encoded PIDs, the recoded Wt reads that we detected were less frequent in dual-selection experiments than control selections (Supplementary Fig. 3d). Importantly, the frequency of KG variants we detected among NNS-encoded PIDs was indeed strongly reduced with the CGG counterselection PAM at 8 mM 3-AT, when compared to the other conditions tested. Collectively, our results indicate that the KG variant retains the ability to recognize CGGCG PAMs in vivo, albeit less strongly than Wt Cas9. In an effort to evolve a variant that further discriminates against NGG PAMs, we proceeded with additional rounds of selection that incorporate this CGG PAM for counterselection.

### Dual selection in *E. coli* yields a round 2 variant with improved discrimination

The KG variant that we isolated in round 1 selections was effectively bypassing dual selection in our yeast system, so we returned to our B1H system in round 2. We generated two additional ω-dCas9 libraries wherein error-prone PCR was used to randomly mutagenize a region within the KG variant’s PID (or within Wt Cas9’s PID for comparison) spanning codons 1101–1342 and part of codon 1100. Screening of the Wt library with our dual-selection plasmid on plates containing 8 mM 3-AT yielded non-conservative substitutions at position 1333 in all 21 of the sequences recovered (Supplementary Table 4). Mutation of R1333 is expected to reduce recognition of the CGG negative-selection PAM^40^. As anticipated, positive selection alone was much less stringent for substitutions at 1333 (4/24 isolates; Supplementary Table 5). No identical substitutions at other positions were observed more than twice in either experiment.

**Fig. 3:**
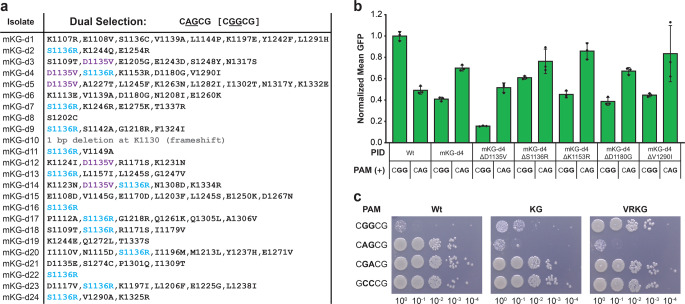
Identification of D1135V/S1136R substitutions that augment NGG discrimination within the KG background. **a** List of non-synonymous PID substitutions identified in a dual-selection ω-dCas9 library experiment with random mutagenesis in the KG variant’s PID. The reporter plasmid’s PAM configuration is displayed above with the counterselection PAM in brackets. Cyan and violet lettering was used to emphasize the S1136R and D1135V substitutions, respectively, which were multiply represented. **b** GFP activation assays comparing Wt to the mKG-d4 isolate and its single-reversion (Δ) derivatives as indicated, with normalization as in Fig. 1b. The PAMs present in each single-target reporter plasmid is indicated below. Error bars, mean ± s.d. (*n* = 3, biological replicates). **c** Plasmid cleavage assays comparing catalytically intact Wt, KG, and VRKG plasmids co-transformed with the single-target plasmids tested in Fig. 1c. 10-fold serial dilutions were plated in parallel on rich media with antibiotics to select for both plasmids and a minimally inducing concentration of IPTG (2.5 µM) for Cas9 expression. Source data are available in the Source Data file.

In contrast, when the KG library was screened with dual selection (Fig. 3a), a S1136R substitution was observed in 14/24 sequences, and a less-frequent D1135V substitution was also observed (5/24 sequences). With positive selection alone, no identical substitutions were observed more than twice at any position (Supplementary Table 5), so we proceeded to test a few of the dual-selection isolates in activation assays. The mKG-d4 isolate—which included both the S1136R and D1135V substitutions—displayed exceptional activity with the CAG reporter relative to the other candidates tested (Supplementary Fig. 4). When each of its five amino acid substitutions were reverted to that of the KG parent, the S1136R substitution was apparently required for improved discrimination against CGG PAMs, whereas the D1135V substitution was apparently required for maintaining robust CAG activity when combined with S1136R (Fig. 3b). After combining these two VR mutations with the parental KG mutations, we further confirmed that the quadruple substitution (henceforth, ‘VRKG’) is sufficient for bypass of our dual-selection reporters in B1H-dependent growth assays up to 8 mM 3-AT (Supplementary Fig. 5). To corroborate these results, we compared our evolved variants to Wt Cas9 in a bacterial plasmid cleavage assay. When single-target reporter and Cas9 plasmids are co-delivered, reduced colony formation on media with antibiotics that select for maintenance of both plasmids is indicative of target plasmid cleavage. For this purpose, the Wt, KG, and VRKG PIDs were cloned into a catalytically active and IPTG-inducible Cas9 backbone that lacks N- or C-terminal fusions. Cleavage of the CGG target plasmid by VRKG was undetectable upon plating with a minimally inducing concentration of IPTG, whereas the KG variant exhibited an intermediate phenotype when compared to Wt (Fig. 3c). However, when the same culture dilutions were plated with a maximally inducing concentration of IPTG, strong cleavage of the CGG target plasmid was clearly apparent with either the KG or VRKG variants (Supplementary Fig. 6b). Given that we could still detect CGG recognition with the VRKG variant in both cleavage and activation assays, we sought to further evolve its discrimination against NGG PAMs by increasing counterselection stringency in the next round of selection.

### Zinc finger fusions can improve counterselection stringency

It was previously shown that zinc finger (ZF) or TALEN DNA-binding domains can be fused to Cas9 to compensate for PAM-binding deficiencies in eukaryotic cells and thereby restore cleavage activity at targets where the PAM interaction is limited^14^. We postulated that the KG variant had survived canavanine counterselection in round 1 due to its attenuated recognition of the CGG PAM in the *CAN1* locus, and that such deficiencies could be overcome with ZF-mediated complementation. Using single-target *LYS2* selection strains, we first tested whether targets with a CGG PAM could be efficiently cleaved by the KG or VRKG variants if a strong ZF interaction was provided. We inserted the Zif268 consensus (Zif268c) binding site 13 bp downstream of the *hEGFP* protospacer and co-delivered PIDs with our standard yeast Cas9 backbone or a backbone containing the three ZFs of Zif268 fused to Cas9’s C-terminus. The KG variant, and to a lesser extent the VRKG variant, was able to cleave at virtually Wt levels only when the PIDs were assembled with a Cas9-Zif268 backbone in a strain that harbored the Zif268c binding site (Supplementary Fig. 7a). We next generated a dual-selection strain with the Zif268c binding site inserted only at the *CAN1* locus (Supplementary Fig. 7b, c) and validated this system with clonal PID fragments assembled into the backbones tested above. Dual-selection media strongly impaired plating efficiency with the KG and VRKG variants only when the Cas9-Zif268 backbone was provided, whereas plating efficiency with positive selection alone was similar with either backbone (Supplementary Fig. 7b).

Having established a system with improved counterselection stringency, we followed up with a library selection experiment where we randomly mutagenized the VRKG variant within its PID region using error-prone PCR (effectively mutagenizing residues 1102–1342) and assembled these insert fragments into the Cas9-Zif268 backbone upon delivery. Unfortunately, dual-selection media yielded very few colonies, and no identical substitutions were observed more than twice at any position of the PID in either positive-selection or dual-selection experiments (Supplementary Table 6). Failing to detect evolution in either of these experiments, we proceeded with additional assays to characterize the KG and VRKG variants from our first 2 rounds.

### Evolved variants exhibit altered PAM preferences

**Fig. 4:**
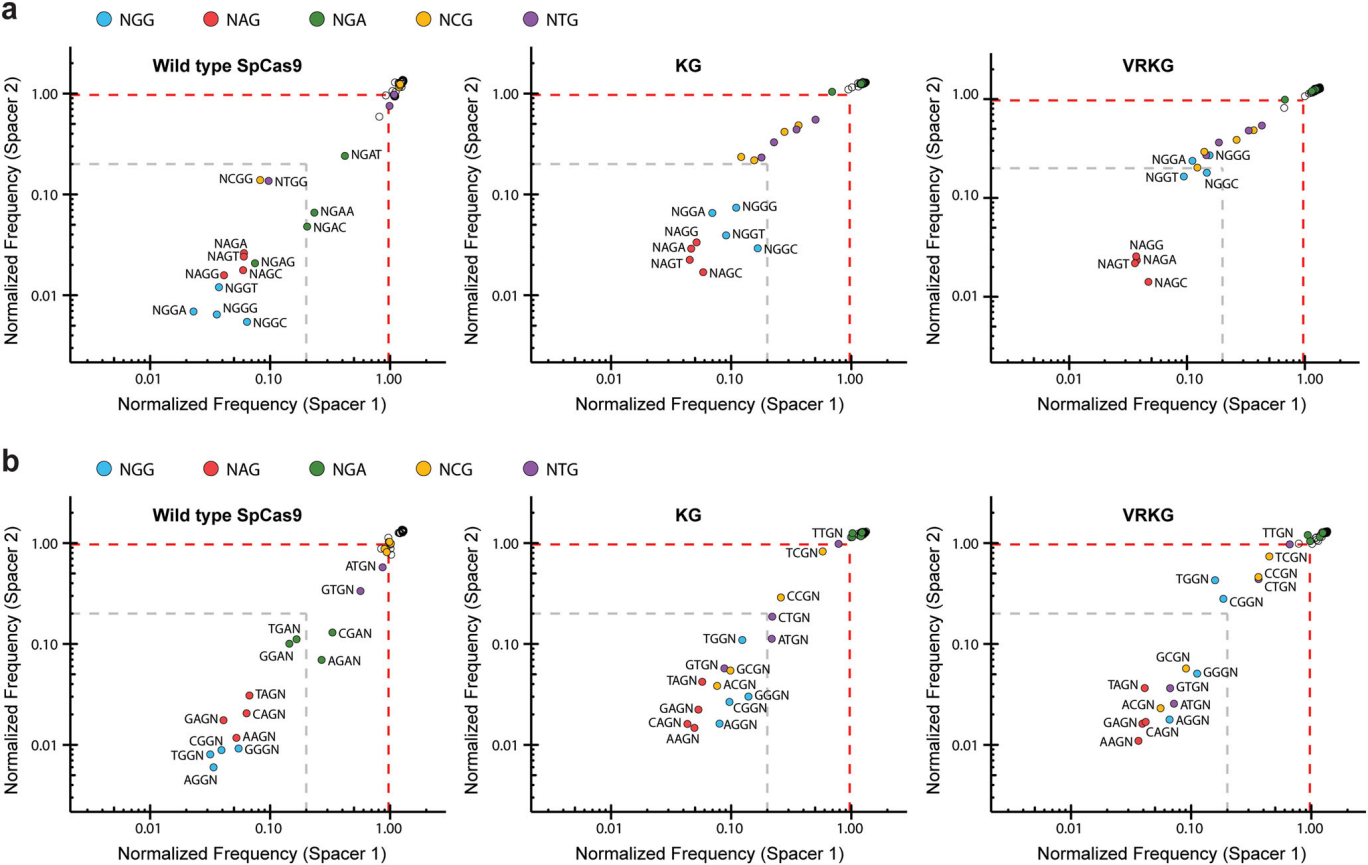
PAM repertoire profiling for the KG and VRKG variants. **a** Plasmid cleavage assays with a randomized PAM library. Wt, KG, or VRKG Cas9 plasmids programmed with a targeting spacer in their sgRNA scaffold (Spacer 1 or Spacer 2) were co-delivered with the PAM library and plated with 10 µM IPTG and antibiotics to select for both plasmids. After deep sequencing, PAM frequencies were calculated for each pool of double transformants and normalized to their frequencies in the pre-depletion PAM library. For each spacer (*X* or *Y* axis), normalized PAM frequencies were plotted with binning for all possible sequences at PAM positions two through four. Gray dashed lines denote a normalized frequency value of 0.2 (fivefold depletion). Red dashed lines denote a normalized frequency value of 0.977; none of the normalized frequency values from Wt dCas9 control experiments with either spacer were lower than 0.97 when rounded to the nearest hundredth (Supplementary Data 1). **b** Same data from experiments in panel **a**, but re-plotted with binning for all possible sequences at PAM positions one through three. Source data are available in the Source Data file.

To more comprehensively define the PAM specificity of our KG and VRKG variants, we employed an in vivo PAM-depletion assay similar to those described previously^5,7^. In our experiments, we co-delivered each Cas9 expression plasmid with a target plasmid library completely randomized at the first eight positions of the PAM, and then performed targeted deep sequencing on DNA from the co-transformed pools and raw PAM library to identify depleted PAMs. We targeted two protospacers abutting the randomized region in either orientation, and initially tested these spacers with clonal library derivatives harboring the CGG or CAG PAMs from above. While previously testing our evolved variants in plasmid cleavage assays with the *hEGFP* spacer, 2.5 µM IPTG was found to be sufficient for robust cleavage of the CAG target plasmid (Fig. 3c). However, when the VRKG variant was tested with library-targeting spacers we found that 10 uM IPTG was required to recapitulate this effect (Supplementary Fig. 8), so we proceeded with 10 uM for the library experiments. After normalizing reads in accordance with the pre-depletion PAM library, we initially plotted PAM frequencies for each variant or the Wt control by binning with different sequences at positions 2–4 of the PAM (Fig. 4a). Whereas Wt results were generally consistent with published work^7^, characterization of the KG and VRKG variants revealed an evolved preference for NAG PAMs, with the KG variant apparently more permissive for NGG PAMs than the VRKG variant. When we binned with the first 3 positions of the PAM, interestingly, we found an unexpected permissiveness for RNG PAMs (where ‘R’ represents A or G) in addition to NAG PAMs within the VRKG experiments (Fig. 4b; Supplementary Fig. 9). This implies that our directed evolution efforts to exclude NGG PAMs with negative selection ultimately resulted in discrimination against YGG PAMs (where ‘Y’ represents T or C), as well as the other YBG PAMs (where ‘B’ represents G, T, or C). Certain RNG PAMs such as ACG and GCG were likewise recognized by the KG variant (Fig. 4b; Supplementary Fig. 9), but neither variant retained Wt Cas9’s permissiveness for NGA PAMs (Fig. 4 and Supplementary Fig. 6b).

Having found that the KG and VRKG variants accommodate non-NAG and non-NGG PAMs, we sought to clarify whether their PAM repertoires had been broadened overall. We determined the total number of unique five-bp PAMs depleted by each variant and found that Wt, KG, and VRKG depleted on average 268.5, 254, and 270 of the 1024 possible PAMs, respectively, with the two spacers tested (Supplementary Fig. 10a). Given the striking similarity in the total number of PAMs depleted, we conclude that the specificity of these variants was principally altered rather than broadened. We also compared the total depletion activity achieved with each variant by summing the fold-depletion values measured for all 1024 five-bp PAMs. This analysis revealed greater total depletion with Wt than with KG or VRKG (Supplementary Fig. 10b), consistent with our results from other assays where maximal activity was only achieved using Wt Cas9 and NGG PAMs (Figs. 2d and 3b, Supplementary Fig. 2).

As an additional test of their activity, we benchmarked our evolved variants against Wt and two previously engineered PID variants^7,16^ using an established assay for EGFP knockdown in transiently transfected human cells^7,49,50^. When compared to Wt Cas9 with published sgRNAs^7^, the VRKG variant exhibited commensurate or somewhat improved activity across the four NAG PAMs tested (Supplementary Fig. 11a). However, its maximal efficiency was lacking when compared to efficiencies achieved with Wt at NGG PAMs, or the VQR and ‘SpCas9-NG’ (VRVRFRR) variants at their preferred NGA or NG PAMs, respectively (Supplementary Fig. 11b). Moreover, activity above background was clearly detectable when testing the VRKG variant with two YGG PAMs, suggesting that additional engineering would be required to discriminate more reliably against NGG PAMs. Nevertheless, it should be noted that this assay does not allow for direct comparisons between PAMs because sgRNA-specific activities can dominate the overall knockdown efficiency measured across targets^16,51,52^.

**Fig. 5:**
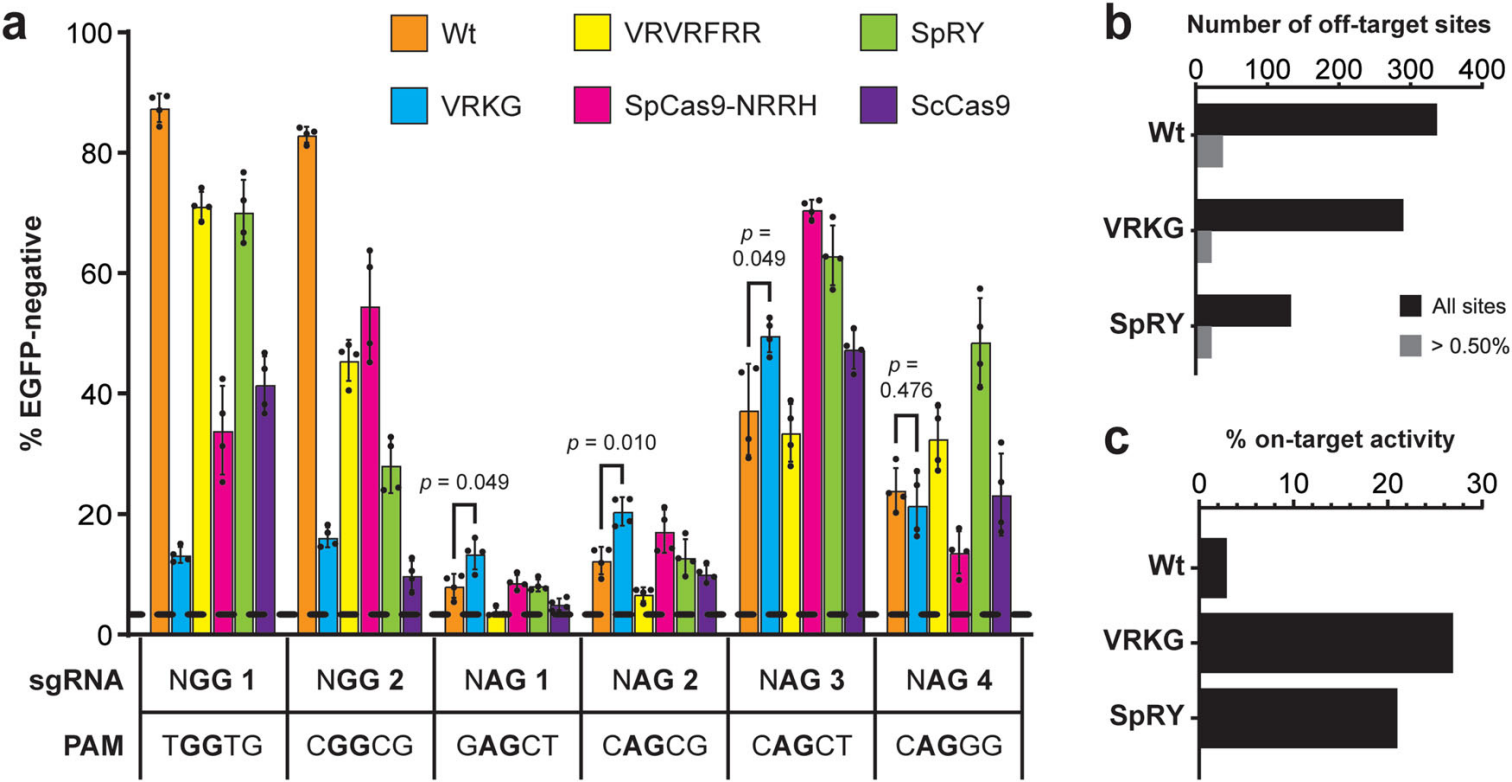
Comparison of VRKG with Wt Cas9 and other NAG-permissive variants in human cell culture targeting assays. **a** Disruption of a chromosomally integrated constitutive EGFP cassette in the U2OS background, using catalytically active wild-type or engineered variants with NGG- or NAG-targeting sgRNAs as indicated. EGFP knockdown activity is plotted as the percentage of EGFP-negative cells, determined from transiently transfected populations of single cells analyzed by flow cytometry. sgRNA sequences and nomenclature were designed previously^7^. *p* values calculated from two-tailed *t* tests comparing Wt Cas9 and VRKG with each NAG-targeting sgRNA are shown. Error bars, mean ± s.d. (*n* = 4, biological replicates). Black dashed line denotes the EGFP-negative background level determined from experiments with a non-targeting sgRNA. **b** Quantification of GUIDE-seq off-target sites detected for Wt Cas9, VRKG, and SpRY with the ‘HEK site 4’ sgRNA in U2OS.EGFP cells. Bars quantify all off-target sites (black bars) or only those off-targets that contributed >0.50% of summed GUIDE-seq read counts (gray bars) detected in each experiment. **c** Percent on-target activity, determined for each variant from the fraction of their on-target GUIDE-seq read counts out of summed GUIDE-seq reads detected overall. Source data are available in the Source Data file.

While this manuscript was in preparation, engineered variants with broadened PAM repertoires that include both NAG and NGG PAMs were described, such as the SpCas9-NRRH and SpRY variants with reported preferences for NRRH or N(R > Y) PAMs, respectively (where ‘H’ represents A, T, or C). To probe their on-target activities in relation to Wt Cas9 and VRKG, we repeated the EGFP knockdown assay with each of these variants and the four NAG and two NGG sgRNAs tested above. The VRVRFRR variant was also included in this wave because it has been shown to offer some activity on NA PAMs, despite its preference for NG PAMs. In addition, we tested the naturally occurring *Streptococcus canis* Cas9 (ScCas9) ortholog because of its reported tolerance for NNG PAMs and ability to utilize SpCas9’s sgRNAs^13^. Maximal NAG-targeting efficiency across the four guides was achieved using the NAG-3 sgRNA with SpCas9-NRRH, followed by SpRY, VRKG, and ScCas9, but neither natural nor engineered variants outperformed VRKG with the two lowest efficiency NAG-targeting guides (Fig. 5a). When considering all five positions of the PAM, relative targeting activities generally fell in line with the PAM preferences reported for each variant. For instance, SpCas9-NRRH and SpRY offered formidable activity on the various NGGH and NAGH PAMs tested, but SpRY, VRVRFRR, and even Wt Cas9 were favored with the NAG-4 sgRNA that targets a CAGGG PAM with a ‘G’ in the fourth position^5,16–18,52,53^. As a final comparison in this cell line, GUIDE-seq was performed to probe the off-target profiles of Wt, VRKG, and SpRY variants with a previously published sgRNA^17,54^. The on-target site for this sgRNA harbors a GGGTT PAM which in principle could be recognized by each of these variants (Supplementary Fig. 12). Though the fewest number of off-target sites were detected with SpRY, the highest percentage of on-target activity was measured with VRKG (Fig. 5b, c, and Supplementary Data 3). Expectedly, many of VRKG’s top-scoring off-target sites included PAMs that conform to NAG or RNG (Supplementary Fig. 12).

## Discussion

We describe here the engineering of two in vivo reporter systems that provide simultaneous positive and negative selection, or positive selection alone, to facilitate directed evolution of Cas9’s PAM specificity. Our ω-dCas9 system was derived from a plasmid-based B1H system that was previously used to evolve synthetic zinc fingers via *HIS3*-dependent positive selection and *URA3*-dependent negative selection with 5-fluoroorotic acid (5-FOA)^34^. Instead of the dual-promoter design used for zinc finger evolution, the ω-dCas9 system exploits a single-promoter architecture that allows positive selection stringency to be tuned with dCas9-dependent repression at a downstream secondary target (Fig. 1a). This provides negative selection without requiring 5-FOA-based counterselection. Using this system, positive selection alone is also attainable with isogenic reporter plasmids that either have a negligible PAM sequence installed at the repressive site (see Supplementary Fig. 13 for further validation of the NCC PAM used in this work) or lack the secondary protospacer altogether. In contrast, the yeast selection system we constructed enables screening of generally smaller library sizes than our ω-dCas9 system, but it allows both positive- and dual-selection isolates to be obtained from the same transformation mixture simply by plating on different media (Fig. 2). Its cleavage-dependent readout ensures that selected variants are not merely binding proficient, and offered sufficient stringency to detect evolution with positive selection alone (Supplementary Table 3). In principle, homology-directed assembly in vivo allows for successive rounds of selection in yeast without subcloning in *E. coli*. Furthermore, the use of independent positive- and negative-selection markers permits an asymmetric increase in counterselection stringency without perturbing positive selection (Supplementary Fig. 7).

Our use of negative selection in this study facilitated the isolation of Cas9 variants that partially discriminate against YGG PAMs. This was particularly clear in round 2, where the additional substitutions did not significantly alter activity on the CAG positive selection PAM, when compared with the KG parent in our bacterial GFP assay (Figs. 2 and 3; two-tailed *t* test, *p* = 0.6149). In other words, the evolution we observed in round 2 was effectively contingent on bypass of the CGG counterselection PAM. Furthermore, isolation of the KG variant from our site-saturation library in round 1 only modestly improved activity on CAG relative to Wt but strongly reduced activity on CGG (Fig. 2d and Supplementary Fig. 2b), and the latter effect stimulated near-complete bypass of counterselection in yeast. When we examined the other non-Wt variants isolated with positive selection alone, the three variants which maintained both arginines at 1333 and 1335 were found to be sensitive to canavanine, indicating that they had cleaved at the CGG counterselection PAM (Supplementary Table 3). Neither Wt nor non-Wt isolates with both arginines intact were detected among the accompanying dual-selection isolates. Notwithstanding, it should be cautioned that negative selection can impede step-wise traversal through evolutionary trajectories which may otherwise lead to altered specificity, for example, as in cases where crucial generalist intermediates are accessible with positive selection alone^22^. Consistent with this notion, we found that the CGGCG counterselection PAM constrained the growth of our KG intermediate more stringently than the TGGAG counterselection PAM in B1H assays, and consequently reduced KG’s frequency in evolved populations (Supplementary Fig. 3). Its relative ability to survive CGGCG counterselection more robustly in yeast than in bacteria was fortuitous, and suggests that the original canavanine-based SSA reporter we designed without ZFs might have lower effective sensitivity than our B1H dual-selection plasmids. Moreover, we found that mild enrichment for this variant in yeast was detectable even in the absence of negative selection (Supplementary Table 3).

**Fig. 6:**
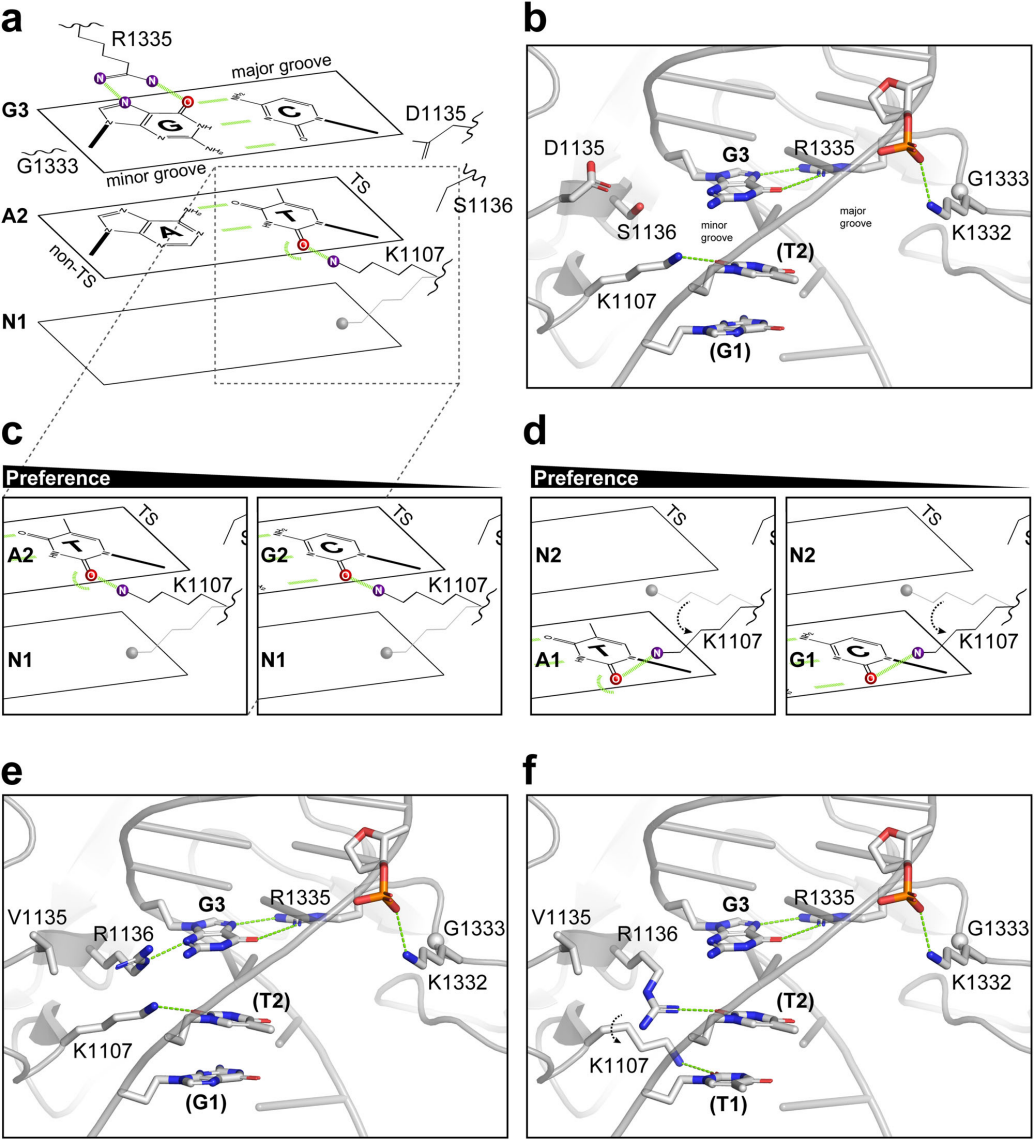
Molecular modeling of the KG and VRKG variants’ altered PAM recognition. **a** Schematic summary of the KG variant’s base-specific interactions with a NAG PAM. Major-groove interactions between R1335 and the non-target strand (non-TS) are maintained, as is the minor-groove interaction between K1107 and the target strand (TS). **b** Three-dimensional model of the KG variant’s PID interactions with a CAG PAM, based on a published crystal structure of PAM-bound Cas9^55^ (PDB 5F9R). Relevant target strand nucleobases are labeled in parentheses. The D1332K, R1333G, (A1) → (G1), and (C2) → (T2) substitutions were introduced with PyMOL for visualization purposes. One of several K1332 rotamers in range for contacting the phosphate backbone is shown (dashed green line). **c** Schematics as in panel **a**, but focusing on K1107’s minor-groove interaction with the O2 acceptor of a TS pyrimidine (boxed inset, dashed gray lines). Thymine harbors more free electrons (curved dashed green line) at its O2 acceptor than cytosine when they are Watson–Crick paired in duplex DNA. **d** Schematics as in panel **c**, but summarizing a putative alternative interaction between K1107 and either (T1) or (C1) nucleobases at position 1 of the TS. **e** Three-dimensional model as in panel **b**, but for the VRKG variant. R1136 is in range for contacting G3’s acceptor group in the minor groove, without clashing with K1107. **f** Three-dimensional model as in panel **e**, but with an AAG PAM. When the alternative K1107 rotamer is adopted, R1136 may also adopt an alternative rotamer in range for minor-groove contacts with (T2) of the TS.

The molecular and functional consequences of the KG and VRKG substitutions can be further interpreted in light of prior structural work on PAM-bound Cas9^55^. The R1333G substitution ablates a base-specifying major-groove contact with guanine at position 2 within NGG PAMs and may provide added flexibility to the adjacent K1332 residue, facilitating formation of a stabilizing interaction with the phosphate backbone (Fig. 6a, b). In turn, the KG variant’s partial preference for NAG PAMs over NGG PAMs likely results from a K1107-mediated minor-groove preference for thymine at position 2 on the target strand (Fig. 6c), given that thymine’s O2 acceptor is more electronegative than cytosine’s O2 in the minor groove of B-DNA^56,57^. The proposed K1107 preference would be masked in Wt Cas9 with R1333 intact, due to dominant specification of NGG PAMs via the major groove. Based on our modeling of an alternative K1107 rotamer in the minor groove, we further speculate that lysine 1107 can stably interact with thymine or cytosine at position 1 of the target strand (TS) in lieu of interacting with a nucleobase at position 2 (Fig. 6d). In other words, minor-groove interactions with the TS nucleobase at position 1 may compensate for weaker interactions occurring at position 2 when a thymine is absent there, as in non-NAG PAMs. Thus, K1107’s preferences in the minor groove could help to explain why the KG variant was permissive for certain non-NAG PAMs, especially those that conform to VGG (where ‘V’ represents G, A, or C) or RBG. In our model, K1332’s putative interaction with the TS backbone partially compensates for the loss of a major-groove contact at 1333 by adding non-specific affinity, and does not contribute to base-recognition directly. If K1332 also helps to stabilize specific interactions between lysine 1107 and the TS nucleobase at position 1, the effect is presumably indirect. However, this model does not address the other PAM hierarchies that we detected in experiments with KG, such as the preference for CGG over TGG, or ACG over ATG (Fig. 4b). Our functional interpretation of the VRKG variant’s S1136R substitution is more speculative, but we identified two arginine rotamers in range for minor-groove contacts with either the guanine at position 3 of the non-TS (Fig. 6e) or the second nucleobase of the TS (Fig. 6f). The former replaces a water-mediated interaction between guanine and S1136 that was identified in Wt Cas9^40^, whereas the latter requires that K1107 is simultaneously rotated to allow contacts with position 1 of the TS and avoid sidechain clashes (Supplementary Fig. 14a). In either of these proposed scenarios, the D1135V substitution could serve to accommodate R1136’s aliphatic side chain through a hydrophobic interaction. VRKG’s improved discrimination against YGG PAMs, and more generally YBG PAMs, may reflect a stricter reliance on interactions between K1107 and either a thymine or cytosine at position 1 of the TS, at least with non-NAG PAMs lacking a TS thymine to anchor K1107 at position 2 (Supplementary Fig. 14b). This discrimination was not absolute, and was surmounted by overexpression in *E. coli* (Supplementary Fig. 6b) or ZF-mediated compensation in yeast (Supplementary Fig. 7), suggesting that the evolved preference reflects only modest differences in PAM affinity. Indeed, ZF fusions are proposed to facilitate target searches by increasing the local concentration of Cas9 in the vicinity of PAMs^14^, rather than stabilizing transitions to a cleavage-proficient state or other steps in the cleavage mechanism^4^.

The results presented in this work highlight certain nuances concerning the usage of engineered selection systems and the evolution of Cas9’s PAM specificity. Evidently, our use of a single CGG counterselection PAM did not prevent the alternative RNG recognition modality from evolving in VRKG. In order to evolve further discrimination against non-NAG PAMs, or at least the RYG subset, it is possible that putative interactions between K1107 and position 1 of the TS could be excluded by screening VRKG libraries with negative selection configured against RBG PAMs. This might require that affinity for NAG PAMs is first bolstered through additional non-specific contacts, so that more aggressive base-specifying substitutions are tolerated in downstream engineering. However, non-specific contacts might instead favor broadening of the PAM repertoire, as reported for the VRVRFRR variant^16^. A perhaps more effective strategy for engineering NAG specificity would focus on generating major groove contacts that provide both affinity and specificity simultaneously. In principle this could be achieved using positive selection alone; prior work on directed evolution has established that positive selection systems are sufficient to evolve proteins with altered substrate specificity, at least in certain contexts^22,25^. For example, positive selection for improved recognition of NGA PAMs was successfully employed in engineering of the previously described VQR variant^7^, which robustly discriminates against NGG PAMs as a result of three PID substitutions. Increased activity on NGA PAMs was evidently sufficient for outcompeting parent proteins in selection assays and was accompanied by a concomitant loss in permissiveness for NGG PAMs, although the latter property was not selected per se. Our inability to evolve a major groove contact in place of R1333, analogous to the R1335Q substitution in VQR, likely constrained the maximal activity of our variants on NAG PAMs. Prior efforts to engineer specificity for NAAG converged on seven PID substitutions, including R1333Q/R1335Q/T1337R and the D1332K substitution we fortuitously recovered in our study, but produced a kinetically impaired variant^41,58^. We speculate that the SpCas9 PID is refractory to substitutions that robustly specify interactions with an adenine at position 2 of the non-TS, unlike the PID of *Streptococcus macacae* Cas9^59^. It should be recognized, however, that our positive selection was configured with only a single protospacer and a single subtype of the NAG PAM throughout this work. This is clearly distinct from the positive selection conditions imposed on natural type II CRISPR-Cas systems in phage-infected hosts, where Cas9 and its orthologs have access to a cohort of PAM subtypes during the spacer acquisition and targeting stages of CRISPR immunity^60–62^. The protospacer and PAM sequence diversity available to Cas9 variants in adapting populations may help to buffer against PAM specificity bottlenecks, facilitating step-wise evolution over time. Indeed, the use of a mixed PAM cohort during directed evolution of the xCas9 variant was postulated to have dampened positive selection stringency^17^. Anticipating that even small differences in the PAM preference of evolving variants can influence the selection stringencies elicited by PAM substrates, future directed evolution work could emphasize parallel screens with closely related positive or negative selection PAMs to ensure that diverse evolutionary intermediates are comprehensively sampled.

## Methods

### Microbial strains and growth conditions

Except as noted otherwise, *E. coli* strains XL1-Blue, TOP10, C2925 (*dam-/dcm-* from NEB), or US0 *ΔhisB, ΔpyrF*, *ΔrpoZ::zeo(Zeo*^*R*^*), F*′*[lacI*^*q*^*Z∆MI5 Tn10(Tet*^*R*^*)]* (ref. ^63^) were cultured for 16 h at 37 °C on a roller drum in sterile 14 mL round-bottom Falcon tubes (Corning) containing 5 mL 2xYT broth (Fisher), or for 12–16 h at 37 °C on sterile petri dish plates containing 2xYT/Bacto agar media (2xYT broth granules + Bacto agar powder, Fisher, adjusted to pH 7.0 after dissolution but before autoclaving) supplemented with glucose (2%) after autoclaving. Where applicable, media was supplemented with kanamycin (50 µg/mL) and/or carbenicillin (100 µg/mL) to maintain plasmids, and was supplemented with tetracycline (15 µg/mL) only when preparing chemically competent or electrocompetent XL1-Blue and US0 cells. 2xYT plates containing IPTG (2.5, 10, or 100 µM) were not supplemented with glucose, and were prepared from 2xYT agar powder (Fisher). The minimal media recipes for selective NM broths or Bacto-based selection plates supplemented with 3-AT (2, 5, or 8 mM) were described previously^64^; the recipe for non-selective NM broth is identical to the former but lacks 3-AT and is supplemented with histidine (0.1%). Minimal media used in this work was always supplemented with kanamycin, carbenicillin, and 100 µM IPTG. To build *E. coli* plasmid libraries in liquid culture, transformants were pooled into a 2 L flask containing 1 L SOB supplemented with glucose (1%) and recovered by shaking for 1 h at 37 °C before the culture was supplemented with the appropriate antibiotic; additional time for growth was then allowed under the same conditions until the culture was harvested for maxiprepping at ~0.6 attenuance (*D*_600 nm_). Otherwise, maxiprep-scale cultures were grown for 16 h at 37 °C in 2 L flasks containing 1 L 2xYT broth supplemented with appropriate antibiotics. To build *E. coli* plasmid libraries on plates, transformants were recovered for 1 h at 37 °C on a roller drum in sterile 15 mL conical-bottom tubes (Corning) containing 10 mL SOB supplemented with glucose (0.5%), and the cultures were then transferred to 4 °C for overnight storage after aliquots were withdrawn for serial dilution and plating. Colonies were counted the next day to quantify colony-forming unit (CFU) concentrations, and cultures were pelleted and resuspended in a smaller volume for plating on sterile 245 × 245 mm BioAssay (Corning) plates (~1.0e7 CFU per plate) containing 2xYT agar media supplemented with glucose (2%). All *S. cerevisiae* yeast strains employed in this work are derived from BY4741 (ref. ^65^; *MATa* *his3Δ1 leu2Δ0 met15Δ0 ura3Δ0*). Except as noted otherwise, BY4741 and its derivatives were cultured for 24 h at 30 °C on a roller drum in sterilized 18 × 150 mm disposable round-bottom glass tubes (Fisherbrand) containing 5 mL YEPD broth (Fisher), or for 44–48 h at 30 °C on sterile petri dish plates containing YEPD agar media. Where applicable, SC minimal media formulations (Sunrise Science) with leucine and/or uracil dropped out were used in place of YEPD to maintain YAC vectors. To prepare yeast competent cells, single colonies were inoculated for overnight growth and then subcultured into 25 × 150 mm glass tubes containing 20 mL of the same broth (1:100 for YEPD or 1:70 for SC). After 4 h (YEPD) or 5.25 h (SC) of growth, subcultures were harvested for washing and lithium acetate transformations using a method similar to the high-efficiency protocol described previously^66^. Each subculture yielded four transformation reaction aliquots; multiple subcultures were prepared from the same starter culture and pooled after washing if additional aliquots were needed. SSA reporter experiments used a quadruple dropout formulation (SC-Arg-Leu-Ura-Lys) for positive selection; this was supplemented with canavanine (60 µg/mL) for dual selection. Transformants required an additional 24 h of incubation at 30 °C for colony formation on dual-selection plates.

### DNA preparation and cloning

All clonal *E. coli* plasmid DNA was purified from 5 mL cultures as described above using miniprep reagents from Qiagen, or from 1 L cultures using maxiprep reagents from Qiagen. Plasmid libraries recovered in *E. coli* were maxiprepped from 1 L cultures or from cells scraped off plates. Liquid cultures were generally pelleted by centrifugation at 4000 x *g* for 5–15 min when prepping DNA. Scraped cells were resuspended in 2xYT broth and centrifuged for 15 min at 4500 × *g*, then aliquoted for maxiprepping (≤1.0 g of pellet mass per maxiprep). To extract DNA from co-transformant pools for deep sequencing, cells were similarly scraped and harvested but aliquoted for miniprepping (≤0.2 g of pellet mass per miniprep). Most PCR fragments used for cloning were generated with Phusion polymerase (Thermo). Taq (NEB) or GoTaq Green (Promega) were used to generate amplicons ≤1.5 kb for colony PCR and deep sequencing, or when a cloning fragment could not be obtained with Phusion. The round 1 PID library and randomized PAM library inserts were generated with Expand (Roche). Manufacturers’ recommended cycling conditions were generally followed for PCRs, except as noted for library inserts obtained with degenerate oligos, or for yeast colony PCRs where 90 s annealing times were used with GoTaq. Yeast colony lysates were generated by resuspending cells in 40 µl NaOH (20 mM), boiling for 7 min, and then centrifuging to pellet the cell debris; 2 µl of the resulting supernatants were used to seed GoTaq PCR reactions. To screen the round 1 PID library in yeast, inserts were re-amplified from the unselected plasmid library and pooled from 24 parallel reactions carried out with Phusion and 12 standard cycles. The round 3 VRKG library insert for screening in yeast was generated by equimolar pooling of two error-prone PCR reactions templated on the pGG403 plasmid at different concentrations (either 6 ng or 30 ng), using the GeneMorph II (Agilent) kit. All restriction-digested plasmid backbones, as well as insert pools used for library ligations in bacteria or assemblies in yeast, were purified from gel-excised bands using either Zymoclean Large Fragment kits (Zymo) or MinElute kits (Qiagen). All other DNA extracted from gels or PCR reactions was purified using Qiagen reagents and MinElute (Qiagen), SpinSmart (Denville), or EconoSpin (Epoch) columns.

Except during library building procedures or as otherwise noted, chemically competent XL1-Blue or TOP10 cells were used for plasmid cloning throughout this work. Occasionally, clones were obtained from electrocompetent XL1-Blue cells by dialyzing Gibson-assembled DNA samples on 0.025 µm membrane filters (Millipore) prior to electroporation. All electroporations were carried out using an Eppendorf Electroporator 2510 configured for 1800 V and 0.1 cm cuvettes (USA Scientific) containing 80 µl of cells pre-mixed with ≤3 µl DNA.

Plasmids used for expression of ω-dCas9 or Cas9 variants in *E. coli* are all originally derived from the pB1H2_UV2-ω-dCas9_UV5-sgRNA plasmid with a sgRNA targeting the heterologous human codon-optimized *EGFP* (*hEGFP*) cassette of U2OS cells^49^. Expression of ω-dCas9 fusions is driven by an IPTG-inducible *lacUV2* promoter, which is a variant of the *lacUV5* promoter with two ‘down’ mutations in the −10 element (previously referred to as *lacUV5m*^63^); expression of sgRNAs is driven by a strong constitutive core promoter derived from *lacUV5*, but with a different spacer sequence between the −10 and −35 elements. This plasmid was built in multiple restriction/ligation steps from synthesized DNA originally bearing EcoRI/NheI restriction sites compatible with an EcoRI- and XbaI-digested backbone derived from pB1H2w2-zif268 (ref. ^63^ and Addgene #18045). The expected sequence of the final plasmid is provided with others in Supplementary Data 4. Unless noted otherwise, construction of each clonal plasmid was verified by Sanger sequencing across all recombinant junctions and all non-backbone regions derived from PCR products or commercially synthesized DNA fragments. The pB1H2_UV2-ω-dCas9::Kan_UV5-sgRNA derivative has an AgeI- and XbaI-flanked kanamycin resistance (Kan^R^) cassette in place of ω-dCas9’s PID, and this plasmid was used as an acceptor vector for scarless ligation of the round 1 PID library insert saturated at positions 1332–1336. The library insert was generated by PCR using oligonucleotide sequences provided in Supplementary Data 5. All commercially synthesized ssDNA oligos and dsDNA fragments were purchased from Integrated DNA Technologies (IDT). Acceptor vector DNA was prepared from a *dam-/dcm-* host strain, C2925, to allow standard restriction digestion at the Dam-sensitive XbaI site. The round 2 random mutagenesis libraries were built from the pB1H2_UV2-ω-dCas9::K2re_UV5-sgRNA (K2re) plasmid, a re-designed acceptor vector with BsaI sites flanking the Kan^R^ cassette. Its BsaI sites produce custom backbone overhangs for scarless ligation with BsaI-digested library inserts generated by error-prone-PCR with oligos oGG732 and oGG738 (effectively mutagenizing residues 1101–1342 and part of the codon for 1100). The BsaI-based insert design ensured that parental plasmids used as PCR templates were not a source of unmutated insert fragments in the subsequent ligation step. The K2re plasmid is a derivative of the pB1H2_UV2-ω-dCas9::K2_UV5-sgRNA (K2) plasmid that was generated by Gibson-assembling DNA fragments with a BsaI-digested K2 backbone; in addition to the features described above, K2re harbors a synonymous substitution in the ampicillin resistance cassette of K2 that eliminates a BsaI site. The K2 plasmid was derived from pB1H2_UV2-ω-dCas9::Kan_UV5-sgRNA and harbors a Dam-insensitive XbaI site to allow preparation from XL1-Blue, but was not used for any libraries in this work. The mKG-d4 reversion series plasmids (pGG393-397) and VRKG equivalent (pGG403) were constructed by Gibson-assembling PCR fragments with a BsaI-digested K2re backbone. To purify ω-dCas9 plasmids directly from library isolates, which also contain reporter plasmids, plasmid DNA was miniprepped from the library isolates and used for transformation of chemically competent XL1-Blue cells. After plating on rich media supplemented with carbenicillin, 5 single colonies were patched on plates with carbenicillin and kanamycin or carbenicillin alone, and a Carb^R^Kan^S^ isolate was chosen for downstream miniprepping. Vectors for expression of catalytically active Cas9 variants in *E. coli* are derived from pGG399, which was initially constructed by Gibson assembling PCR fragments with a ~3.4 kb plasmid backbone liberated from K2re via MluI/XhoI digestion. pGG399 harbors BsaI sites for scarless cloning of spacers into the sgRNA scaffold; both PAM-depletion spacers were initially ligated as annealed oligos into a BsaI-digested pGG399 backbone. In *E. coli*, all catalytically active Cas9 variants were expressed as 1368-residue proteins lacking the N- and C-terminal fusions present in ω-dCas9 plasmids. In the final designs, Cas9 expression was enhanced by replacing the *lacUV2* promoter with the *lacUV5* promoter.

The *E. coli* reporter plasmids employed in this work have one or two *hEGFP* targets installed at the *HIS3/GFP* promoter as depicted in Fig. 1a and Supplementary Fig. 1b; the *GFP* cassette is a yeast codon-optimized version of *EGFP* that is not targeted by the *hEGFP* sgRNA. All constructs tested in Supplementary Fig. 1a, including the R25 plasmid and ‘No Target’ control, were cloned using EcoRI/AgeI-digested inserts and EcoRI/AgeI-digested backbones of the pGHUC plasmid described previously^34^; oligos used for generation of the single-target inserts are listed in Supplementary Data 5. All other reporter plasmids are derived from the R25 plasmid or its derivatives by ligating or Gibson-assembling DNA fragments with backbone DNA that included at least the sequences external to AgeI and NheI in pGHUC. The randomized PAM library was generated by ligating an AgeI/EcoRI-digested library insert with an AgeI/EcoRI-digested backbone of the pH3U3::Amp acceptor vector; this acceptor vector was derived from a pH3U3-based plasmid^67^ by replacement of AgeI/EcoRI-internal sequences with an Amp^R^ cassette. The expected sequence of this library (with Ns representing randomized bases) is presented in Supplementary Fig. 7. The reverse primer employed for insert generation included a 20 bp randomized barcode that was utilized in PAM-depletion analyses. Clonal target plasmids for initial testing of the PAM-depletion spacers were generated with Gibson assembly using AgeI/EcoRI-digested backbone DNA from a random library clone isolated on rich media. This isolate was also used as a template for amplification of fragments from the AgeI/EcoRI insert region, with variable-PAM primers at the EcoRI junction.

*S. cerevisiae* vectors employed for constitutive expression of sgRNA and Cas9 components were all initially derived from two plasmids described previously^68^. The *hEGFP* sgRNA plasmid used for selection experiments, as well as the *LYS2*- or *CAN1*-targeting sgRNA plasmids used for knock-in of the SSA reporters, were each generated by Gibson-assembling a single bridging oligo with NotI-digested pNA0304 acceptor vector backbone. The pNA0306 Cas9 plasmid was used as a PCR template for amplification of backbone and human-codon-optimized Wt Cas9 fragments to generate the PID acceptor vector for round 1 selection experiments. This acceptor vector, pGG211, harbors a NheI/PspOMI-flanked Kan^R^ cassette in place of Cas9’s PID and was Gibson-assembled from gel-purified PCR products. Relative to the pNA0306 reference, a single synonymous mutation in the Cas9 ORF was identified at S145, but disregarded. Non-junction backbone sequences outside of the Cas9 region were not re-sequenced after this assembly. For yeast selection attempts in round 3, the Cas9-Zif268 acceptor vector (pGG442) was generated in two steps by Gibson-assembling fragments with restriction-digested backbone DNA that included at least the sequences external to NheI and XhoI sites in pGG211.

Vectors for expression of Cas9 and sgRNA components in human cells are derived, respectively, from the MSP469 and BPK1520 plasmids described previously^7^. Both plasmids were obtained through Addgene. The MSP469 plasmid encodes the VQR variant; to generate isogenic Wt, KG, VRKG, and VRVRFRR plasmids for testing with VQR, commercially synthesized and PCR-amplified linear DNA fragments were Gibson-assembled with an EcoRV/XhoI-digested backbone fragment from MSP469. Construction of the SpCas9-NRRH, SpRY, and ScCas9 plasmids was carried out similarly, but using a NotI/XhoI-digested backbone from MSP469. For SpCas9 plasmids, non-synonymous substitutions relative to MSP469 were designed to avoid rare codons (<10% frequency) based on human codon usage. For ScCas9, which contains >100 substitutions relative to SpCas9, a mosaic coding scheme was generated with the MSP469 background by transplanting codons present in the ScCas9 sequence available on Addgene (plasmid #117700, associated with ref. ^13^). To clone the sgRNA plasmids, annealed oligos were ligated into an Esp3I-digested BPK1520 backbone.

### *E. coli* plasmid library construction

The round 1 plasmid library insert was constructed using a degenerate oligo essentially as described previously, but for a single step^69^. 15-cycle PCR reactions were pooled from 96 wells, PCR-purified, digested with AgeI/XbaI, and then gel-extracted from a band migrating at the expected length. Approximately 10 ng of pB1H2_UV2-ω-dCas9_UV5-sgRNA plasmid template was used per PCR reaction. The gel-purified insert DNA was ligated overnight at 16 °C with ~20 µg gel-purified backbone DNA that had been likewise digested with AgeI/XbaI (5:1 molar ratio of insert-to-backbone). Ligation reactions were then cleaned and concentrated by ethanol precipitation and split into 15 aliquots of electrocompetent US0 cells for electroporation. After recovering the pooled transformants for 1 h in a 1 L flask, an aliquot was withdrawn for serial dilution and plating to measure the Carb^R^ and Carb^R^Kan^R^ CFUs, and carbenicillin was added to the culture before returning to the shaker. The Carb^R^Kan^R^ CFUs are presumed to result from self-ligation of singly-cut backbone DNA and uncut backbone plasmid; their counts were subtracted from total Carb^R^ counts to estimate the final library size (6.5e7 members). ODs were periodically measured from the library culture by blanking against a similarly treated control culture inoculated in parallel with a proportional volume of electrocompetent cells that were electroporated without DNA. The round 2 plasmid libraries were constructed similarly, except that backbone DNA was derived from the K2re plasmid, and each insert was generated with 16 error-prone PCR reactions using standard oligos and the GeneMorph II (Agilent) kit. The pB1H2_UV2-ω-dCas9[KG]_UV5-sgRNA plasmid or wild-type equivalent was used as template DNA. BsaI-HFv2 (NEB) was used for digestion of both the insert and backbone DNA. Small-scale ligations were initially performed to determine the template concentrations needed to achieve a desired mutation rate for the PID region, according to guidelines described previously^70^, and ~10 ng per reaction was found to be optimal. Mutation rates were estimated by Sanger-sequencing of 10–12 random transformants from each ligation, isolated on rich media. The final library sizes for the mWt and mKG libraries were estimated at 4.7e8 and 4.8e8, respectively, with PID mutation rates of ~1.0% and ~1.2% per bp, respectively. In accordance with procedures used for the round 1 PID library, the randomized PAM library was built using degenerate oligos and a plasmid template, except that 5 or fewer electrocompetent cell aliquots were needed for electroporation, and transformants were selected on plates after overnight storage at 4 °C. The final library size (2.9e7) was estimated as described above for transformant pools except that Kan^R^ and Kan^R^Carb^R^ CFU were enumerated from each transformation aliquot and the net Kan^R^ CFU counts were then summed.

### Construction of yeast selection strains

Selection strains were generated using a CRISPR-assisted knock-in method, essentially as described previously^68^. In brief, parent strains were transformed with the Wt Cas9 expression vector (pNA306) prior to editing, and then sgRNA plasmid (~60 ng) and gel-purified linear donor fragments (~100–300 ng) were co-delivered in a second round of transformation. Transformants were plated on SC-Leu-Ura media to maintain both plasmids, and colonies were patched onto SC-Leu-Ura and either SC-Lys or SC-Arg+Can plates to score for disruption of the *LYS2* or *CAN1* locus, respectively. Candidate SC-Leu-Ura patches were sequenced at their target locus by colony PCR, and sequence-verified isolates were subsequently inoculated from patch scars into YEPD broth to promote loss of the CRISPR plasmids. After overnight growth, cultures were diluted and spread on YEPD plates to obtain single colonies and replica-plated to score for plasmid loss. Strain isolates cured of both plasmids were ultimately transformed with the *hEGFP* sgRNA plasmid (pGG197) for use in downstream selection experiments. Single-target selection strains were generated directly from BY4741. To generate dual-target selection strains, a single-target strain that was cured of only the sgRNA plasmid was used as an intermediate for a second round of editing at the other locus. Donor templates included a single-base synonymous recode to break the PAM of the native target, which, is otherwise regenerated beyond the downstream repeat after knock-in. All donors also included ~40 bp homology arms and were PCR-amplified with synthetic linear templates and oligos listed in Supplementary Data 5; their expected sequences post amplification are listed in Supplementary Data 4. A summary of the *S. cerevisiae* strains used in this work is also provided in Supplementary Data 6.

### Bacterial GFP fluorescence assays

All fluorescence measurements were taken from aliquots of non-selective NM minimal media subcultures grown with IPTG (100 µM) for 20 h at 37 °C. After mixing with a chilled PBS (0.5% FBS) solution, aliquots were kept on ice and analyzed with a SONY SH800 cell cytometer. Mean GFP values were calculated from 30,000 events per culture using compensation settings pre-calibrated with US0 derivatives constitutively expressing either GFP or mCherry alone. Each NM subculture was generated by dilution (1:2000) from independent post-exponential rich media cultures grown for 8–10 h at 37 °C in 2xYT supplemented with glucose (0.5%). Rich media cultures were inoculated from single colonies of US0 co-transformants harboring reporter and ω-dCas9 plasmids. In pilot experiments, 5 mL of each broth was used for culturing and subculturing in round-bottom tubes; otherwise, experiments were carried out with 1 mL of each broth and continuous shaking in 2 mL 96-well V-bottom assay blocks (Corning) that had been autoclaved after pre-seeding wells with 4 mM glass beads (Fisher). For activation or dual-activation/repression experiments, plots were generated by normalizing to the replicate-averaged mean value for Wt ω-dCas9 with a single-target NGG reporter obtained on the same day of the experiment, where applicable. For repression experiments, the single-target NCC reporter with Wt ω-dCas9 was used for normalization. Otherwise, values were plotted without normalization in arbitrary units.

### High-resolution growth curves

Attenuance (*D*_600 nm_) measurements were taken every 10 min from selective or non-selective NM minimal media subcultures grown with IPTG (100 µM) and continuous shaking for 18 h at 37 °C in a Synergy H1 microplate reader (BioTek). For subculturing, 96-well SensoPlate microplates with a clear, flat bottom (Greiner Bio-One) were used. Each 200 µl subculture was generated by dilution (1:100) from independent post-exponential rich media cultures; these 1 mL rich cultures were inoculated and grown in 96-well assay blocks as described above for GFP fluorescence assays.

### ω-dCas9 selection assays on 3-AT plates

All ω-dCas9 experiments using 3-AT- supplemented selective minimal media plates were initiated by co-transforming electrocompetent US0 cells with a ω-dCas9 expression plasmid and a single- or double-target reporter plasmid. For library selection experiments, 1.5 µl of ω-dCas9 library plasmid maxipreps were co-delivered with 1.5 µl of reporter plasmid minipreps concentrated 20–30x by ethanol precipitation (typically corresponding to ~300–400 ng library plasmid and at least 1500 ng reporter plasmid). These conditions favor the formation of double transformants that have received only a single ω-dCas9 library plasmid by providing a molar excess of reporter plasmid, which, is otherwise limiting if unconcentrated minipreps are delivered (the reporter plasmids have a low-copy pSC101 origin of replication). Following electroporation, cells were recovered with rotation at 37 °C in 15 mL conical-bottom tubes containing 10 mL SOB supplemented with glucose (0.5%). After 1.25 h recovery, cells were pelleted by centrifugation at 1900 × *g* for 10 min and then resuspended in 5 mL non-selective minimal media for another 1 h recovery with rotation in round-bottom tubes. Finally, cells were pelleted again, transferred to 2 mL tubes and washed twice in similar minimal media but lacking histidine (3-AT was not added), then resuspended in 1 mL of this media for storage at 4 °C overnight. An aliquot was withdrawn prior to refrigeration for serial dilution and plating on 2xYT media supplemented with glucose (2%) and both antibiotics to quantify the total number of double transformants. Based on Carb^R^Kan^R^ CFU counts recorded the next day, 5.0e7 double transformants from each sample were spread on 150 × 15 mm round plates containing selective minimal media supplemented with 3-AT (2, 5, or 8 mM). Plates were incubated at 37 °C and colony formation was monitored for up to 96 h. Where applicable, serial dilutions were also prepared for plating in parallel on rectangular PlusPlates (Singer Instruments) containing IPTG-supplemented rich media or selective minimal media as described below for the clonal selection experiments. To assess background (false-positive) colony formation post selection, reporter plasmids were initially co-transformed in parallel with a negative control ω fusion plasmid that does not provide activation (pB1H2w2-mutOdd or pGG369), and plated similarly. In most cases, well-isolated single colonies were sequenced directly from colony PCRs, with patching or inoculation into liquid rich media performed in parallel to isolate each clone for downstream characterization where applicable. Otherwise, colonies were picked for miniprepping before sequencing. To generate round 1 post-selection pools for deep sequencing, the site-saturation library was oversampled ~6x in each replicate by spreading 4.0e8 double transformants onto two BioAssay plates (2.0e8 per plate) containing selective minimal media supplemented with 3-AT (2 or 8 mM). Plates were incubated at 37 °C and retrieved after 44 h or 77 h for 2 mM or 8 mM 3-AT, respectively. After miniprepping DNA from scraped pools, the mutagenized PID region was amplified with a single PCR step (15 cycles) to introduce indices and Illumina adapters for multiplexed sequencing on a NextSeq 500 in two separate runs, using primers listed in Supplementary Data 5. For clonal selection experiments, 1 µl of ω-dCas9 plasmid miniprep was co-delivered with 1 µl of reporter plasmid miniprep. After the recovery and washing steps described above, cells were serially diluted and plated on rectangular PlusPlates (Singer Instruments) containing rich media or selective minimal media supplemented with 3-AT at the indicated concentrations. All plates were supplemented with antibiotics to select for both plasmids as well as IPTG (100 µM), and were photographed at the specified time points after incubation at 37 °C (or after a room temperature incubation as indicated).

### WebLogo generation

WebLogos^37^ were generated using the online web tool hosted at https://weblogo.berkeley.edu/logo.cgi. Default parameters were used except that sequence type was set to amino acid, the small sample correction box was unchecked, and files were exported as Encapsulated PostScripts (EPS).

### Yeast SSA reporter selection assays

All yeast selection experiments were initiated by transforming selection strains with a defined quantity of gel-purified Cas9 plasmid fragments for in vivo assembly. For library selection experiments, 130 ng backbone DNA was co-delivered with PID library insert at 1:0.1 M ratios. Owing to the genetic readout inherent to SSA reporters and our use of a constitutive expression system, this molar ratio was critical for ensuring segregation of different library inserts into separate cells upon transformation, and thus reliable coupling between phenotype and genotype. The total number of transformants plated with positive selection (3.9e5 for the round 1 library and 4.5e4 for the round 3 library) or dual selection (3.9e5 for the round 1 library and 1.2e5 for the round 3 library) was estimated from the concentration of CFU quantified on SC-Leu-Ura media. Well-isolated single colonies were randomly chosen from selection plates for patching onto the same media and cell mass was subsequently picked from patches for sequencing by colony PCR. A proline codon was inadvertently duplicated external to the NheI restriction site during design of the pGG211 backbone used for Cas9 assembly in round 1, and this presumably resulted in the elevated frequency of Q1091P substitutions observed in those selections (Supplementary Table 3). This was corrected in the Cas9-Zif268 (pGG442) and isogenic Cas9 control (pGG431) backbones. For clonal selection experiments, 150 ng backbone DNA was co-delivered with PID library insert at 1:3 M ratios. Cells were serially diluted and plated in parallel on SC-Leu-Ura and positive-selection media (as well as dual-selection media, where applicable). After 48 h at 30 °C (or 72 h for dual selection), plates were photographed and then stored at benchtop for up to 16 h to facilitate scoring by eye. After this time the percentage of *LYS2* + transformants was calculated as the ratio of *LYS2* + *, LEU2* + , *URA3* + CFUs obtained with positive-selection media over the total transformant (*LEU2* + , *URA3* + *)* CFUs obtained on SC-Leu-Ura. All library and clonal PID inserts were flanked by ~60 bp homology arms for assembly into backbones, and were generated with PCR or error-prone PCR using primers oGG629/oGG630. Backbone and insert fragment concentrations were quantified prior to experiments using the Qubit dsDNA Broad Range kit (Invitrogen) for library selections or a NanoDrop Spectrophotometer (Thermo) for clonal selections.

### Analysis of deep sequencing data from round 1 selection pools

Approximately 9–30 million raw paired-end reads (75 × 75) were obtained for each sample with a NextSeq 500 (Illumina). Using UNIX commands, reads were merged with PEAR, and the 15 bp PID sequences encoding 1332–1336 were retrieved from each merged read where a perfectly matching flanking sequence could be identified. For each sample, sequences were translated with EMBOSS Transeq and compiled into a file that lists all the retrieved DNA sequences with their corresponding protein sequences. To calculate parental Wt frequencies, the number of parental Wt DNA sequences in each file was divided by the file’s total number of sequences. In parallel, a second set of files was generated in which only the DNA sequences that conform to the NNS coding scheme (which excludes parental Wt) were listed with their corresponding protein sequences. These latter files were used for the KG and recoded Wt protein frequency calculations, agnostic of DNA sequence. A custom python script was also used to determine the number of reads derived from each DNA coding variant for every NNS-encoded protein, but this analysis was not required for our frequency calculations.

### Plasmid cleavage assays in *E. coli*

All plasmid cleavage experiments were initiated by co-transforming electrocompetent US0 cells with a (d)Cas9 expression plasmid and a single-target plasmid. For clonal plasmid cleavage assays, 1 µl of each plasmid miniprep was co-delivered, and cells were recovered with rotation at 37 °C in 15 mL conical-bottom tubes containing 10 mL SOB supplemented with glucose (0.5%). After 1.25 h recovery, cells were serially diluted and plated on media with antibiotics to select for both plasmids (kanamycin and carbenicillin) or only the Cas9 plasmid (carbenicillin). Plates were supplemented with IPTG or glucose as indicated in the figures and photographed after 16 h at 37 °C. For library cleavage (PAM-depletion) assays, 2 µl of Cas9 (or dCas9 control) plasmid minipreps were co-delivered with 1 µl of the PAM library maxiprep. These conditions inherently favor the formation of double transformants that have received only a single PAM library plasmid, because our unconcentrated Cas9 plasmid preps are typically in molar excess of pSC101-based (low-copy) target plasmid preps. After 1.25 h recovery as described above, cells were pelleted by centrifugation at 1900 × *g* for 10 min, washed twice in 2 mL tubes using fresh broth, then resuspended in a final volume of 1 mL for storage at 4 °C overnight. An aliquot was withdrawn prior to refrigeration for serial dilution and plating on 2xYT media supplemented with glucose (2%) and both antibiotics to quantify the total number of double transformants. Based on Carb^R^Kan^R^ CFU counts recorded the next day, 1.0e7 double transformants from each sample were spread on individual BioAssay plates containing 2xYT media supplemented with IPTG (10 µM) and both antibiotics. Plates were incubated for 10 h at 37 °C and then scraped for miniprepping. To introduce indices and Illumina adapters for multiplexed sequencing on a NextSeq 500, the target plasmid’s randomized regions were PCR-amplified in a single step (15 cycles) from the undiluted miniprep pools or pre-depletion PAM library maxiprep, using unique combinations of primers listed in Supplementary Data 5.

### Analysis of deep sequencing data from the PAM-depletion assay

Approximately 8–22 million raw paired-end reads (36 × 36) were obtained for each sample with a NextSeq 500 (Illumina). Using UNIX commands, each read pair was first assigned a 22 bp identifier by concatenating its 8 bp PAM region and 14 bp of its 20 bp barcode region; multiple read pairs with the same identifier were assumed to result from PCR amplification during Illumina indexing and were counted as only one read pair for that 8 bp PAM in downstream steps. Read counts were tallied separately for each PAM orientation (forward or inverted) when analyzing the pre-depletion PAM library sample. After tallying read counts for all 8 bp PAMs detected in each sample, a custom python script was used to extract tallies from user-defined PAM bins of 5 or fewer fixed bp (as indicated in the plots). Frequencies were calculated for each bin as the ratio of PAM-specific read counts over total counts in that sample. Finally, PAM frequencies for each spacer were normalized to their frequencies in the pre-depletion PAM library, and exported for plotting with ggplot2 in R. Otherwise, to calculate total depletion for all 5 bp PAM possibilities, normalized fold-depletion values were first calculated as the multiplicative inverse of each normalized PAM frequency before summing and plotting.

### Modeling of KG and VRKG substitutions on a PAM-bound Cas9 structure

PyMOL (Schrödinger) was used to visualize the PAM contacts of a previously published structure^55^ (PDB 5f9r), and amino acid or nucleobase substitutions were introduced with the mutagenesis feature. For each amino acid substitution, we manually evaluated all possible sidechain rotamers to identify plausible configurations that positioned polar contacts between 2.8 and 3.5 Å apart without producing steric clashes. The R1332K substitution yielded various plausible rotamers, and we arbitrarily chose one for visualization in all figures.

### EGFP knockdown in human cells

Human cell culture assays for EGFP knockdown were performed essentially as described previously^7^, but with the following modifications. The X-001 program was used on a Lonza 2b nucleofector, and reaction mixtures were accordingly scaled up to 500,000 cells with 2000 ng Cas9 plasmid, 666 ng sgRNA plasmid, and 80 ng ptdTomato. Cells were harvested 52 h post transfection and then kept on ice, but were analyzed on a SONY SH800 cell cytometer with 20,000 events captured per sample. The percentage of EGFP-negative cells in each sample was determined from a subpopulation after gating for the top ~50% of tdTomato-expressing cells; the tdTomato-expressing population was identified by comparing control samples that were untransfected or transfected with ptdTomato alone. Each Cas9/sgRNA combination was tested in quadruplicate by performing two biological duplicates on separate days. Finally, a global average of 3.39% background EGFP loss (denoted with a black dashed line) was estimated from four biological duplicate experiments with a non-targeting sgRNA plasmid (BPK1520) performed on four separate days. The clonal U2OS.EGFP cell line used for these assays was obtained from the Joung lab, and harbors a constitutively expressed *hEGFP* cassette integrated in the genome^49^.

### Statistical testing

All quantitative plots presented in this work were generated using Microsoft Excel, GraphPad Prism, or ggplot2 for R. All *p* values were calculated in GraphPad Prism using unpaired, two-tailed *t* tests with Welch’s correction; Gaussian distributions were assumed.

### Off-target profiling with GUIDE-seq

Culturing and nucleofection of the U2OS.EGFP cell line for GUIDE-seq was performed essentially as described for the EGFP knockdown assays above, except that a Lonza 4D-Nucleofector was used with 200,000 cells per sample, and 100 uM dsODN was co-transfected with the Cas9 and sgRNA plasmids (without ptdTomato). Cells were harvested 72 h post-transfection to extract genomic DNA for library preparation and indexed amplicon sequencing, essentially as described previously^54^. Approximately 4–9 million raw paired-end reads (150 × 150) were obtained in total for each sample using an Illumina MiSeq instrument. Read processing and analyses for off-target sites with no more than 7 mismatches were performed as described previously^54^; sites with unique, molecular-indexed reads that collectively mapped to only one strand of the reference sequence were not scored unless unique, molecular-indexed reads derived from both the positive (+) and negative (−) strands of the ODN tag were also detected for that site. The GRCh38 genome assembly was used as reference, and sequence deviations at the first position of the PAM were not scored as mismatches. A separate reference file containing the expected sequence of the lentiviral insertion that includes *hEGFP* was also queried for off-targets (see Supplementary Data 4 for this sequence), although the lentiviral integration site was not determined.

### Reporting summary

Further information on research design is available in the Nature Research Reporting Summary linked to this article.

## Supplementary information

Supplementary Information

Peer Review File

Description of Additional Supplementary Files

Supplementary Data 1

Supplementary Data 2

Supplementary Data 3

Supplementary Data 4

Supplementary Data 5

Supplementary Data 6

Reporting Summary

## Data Availability

Relevant data supporting the findings of this study will be made available in the published article and its Supplementary Information files, the Sequence Read Archive of the NCBI (BioProject accession codes: PRJNA672767, PRJNA673046, and PRJNA673206), and from the corresponding authors upon reasonable request. Source data are provided with this paper.

## Source data

Source Data
