# Supplementary material for: Engineered dual selection for directed evolution of SpCas9 PAM specificity
Source: Nat Commun. 2021 Jan 13;12:349. doi: 10.1038/s41467-020-20650-x (PMC7807044; doi:10.1038/s41467-020-20650-x)
Supplement: Supplementary file 1 — Supplementary Information [file 41467_2020_20650_MOESM1_ESM.pdf]

Supplementary Information

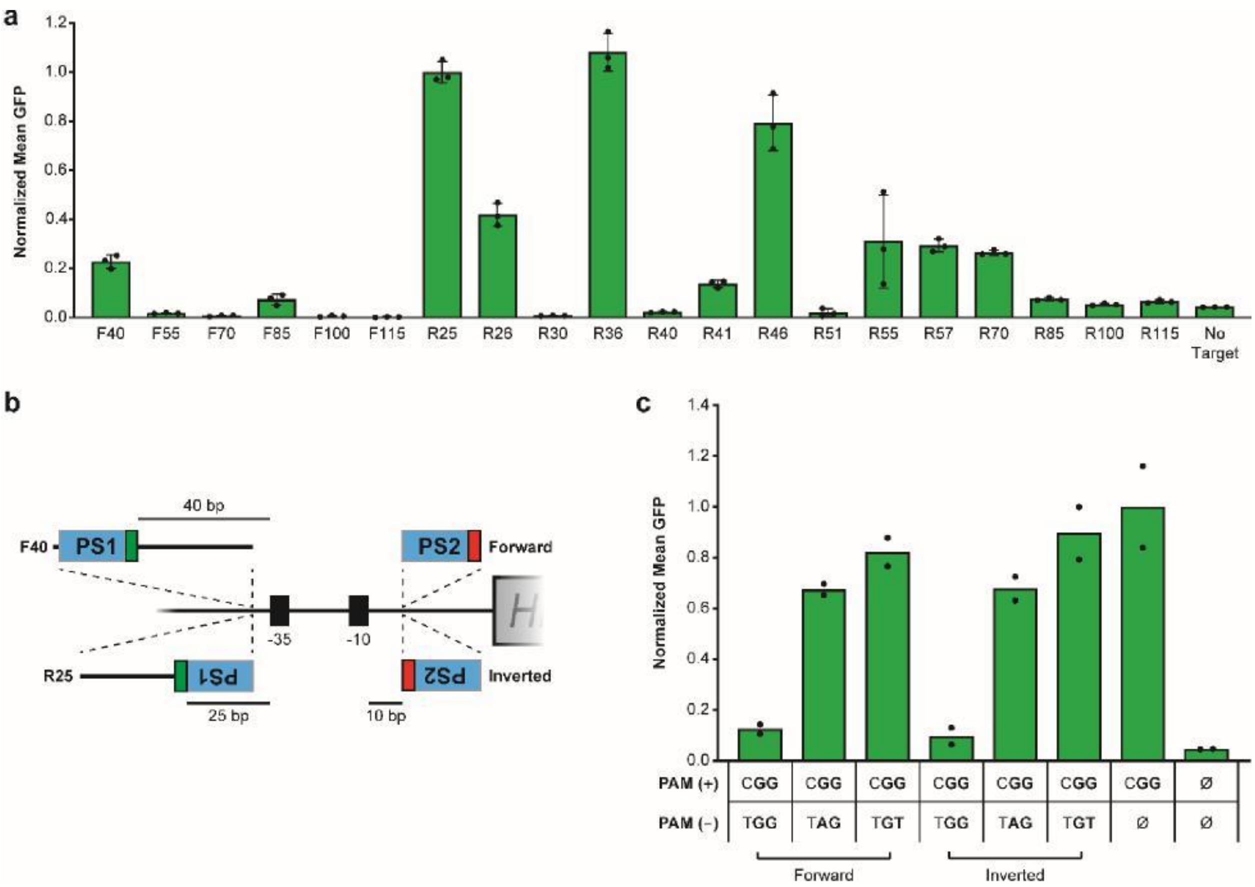

Supplementary Figure S1. Preliminary testing of ω-dCas9-dependent regulation with single- or dual-target reporter plasmids. (**a**) GFP fluorescence measured from *E. coli* cells with Wt ω-dCas9 and reporter plasmids each containing a single protospacer and NGG PAM in different positions and orientations upstream of the *lac* core promoter driving *HIS3/GFP*. The 'No Target' reporter plasmid control lacks *hEGFP* protospacers. Normalized mean fluorescence values were calculated from populations of single cells analyzed by flow cytometry. Error bars, mean ± s.d. (*n* = 3, biological replicates). (**b**) Schematic summary of the different upstream protospacer (PS1) and downstream protospacer (PS2) configurations tested in panels **a** and **c**, with the first three bases of their respective PAMs shown in green or red. Spacing between the upstream PAMs and -35 box, or downstream insertions and -10 box, is indicated in bp. The upstream F40 and R25 configurations tested in panel **a** are shown as examples; other spacing configurations were generated with a variable-length filler sequence fused at the promoter-proximal end of the inserted fragments (delimited by dashed lines). Downstream insertions were all 23 bp in length and identically spaced from the -10 box, but were tested in either Forward or Inverted orientations as indicated. (**c**) GFP fluorescence measurements with Wt ω-dCas9 as in panel **a**, but with various reporter plasmid configurations. PAM sequence at the upstream (+) or downstream (-) protospacer is indicated below; Ø denotes absence of

the protospacer. Orientation of the downstream targets are also indicated (Forward or Inverted). The R25 configuration was used for each of the upstream targets, where applicable. The R25 and ‘No Target’ constructs from panel **a** were re-tested here as controls. n = 2, biological replicates

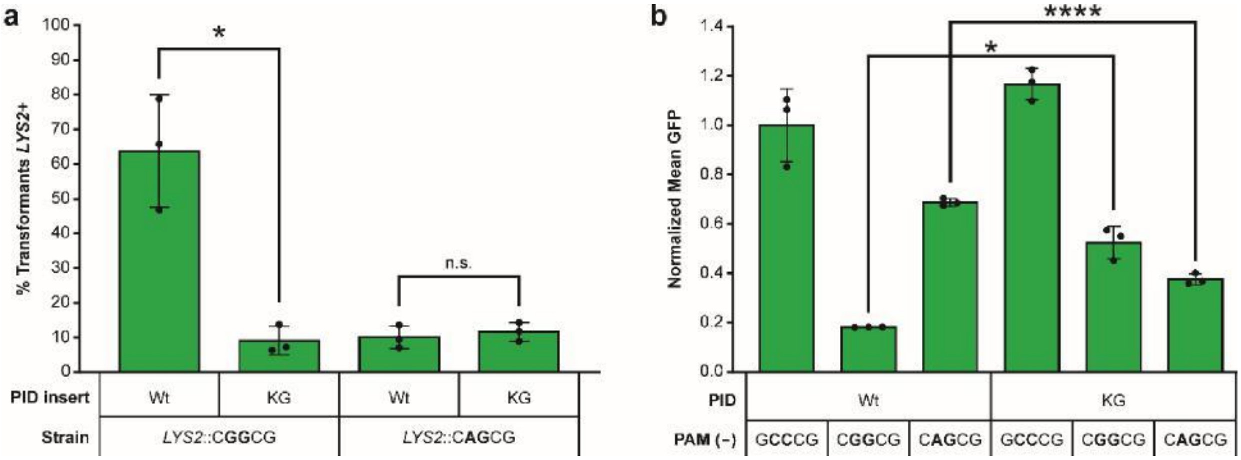

Supplementary Figure S2. Additional validation of the KG phenotype in cleavage and repression assays with CGG and CAG PAMs. (**a**) SSA reporter assays comparing Wt and KG cleavage activities in single-target yeast selection strains. Cas9 backbone was co-delivered with a molar excess of clonal Wt or KG PID insert, and cells were plated in parallel on positive-selection plates and control plates to determine total transformants. The percentage of *LYS2*+ transformants in each replicate was determined from the ratio of positive-selection plate CFUs over control plate CFUs. Error bars, mean ± s.d. ($n = 3$, biological replicates). *, $p = 0.0224$; n.s., $p = 0.5575$ (**b**) GFP fluorescence experiments comparing Wt and KG repression activities in bacteria. Each ω-dCas9 plasmid was co-delivered with a constitutive reporter plasmid harboring only a single counterselection protospacer and one of the indicated PAMs. Mean values were calculated as in **Fig. 1b**, but the NCC PAM with Wt ω-dCas9 was used for normalization purposes. Error bars, mean ± s.d. ($n = 3$, biological replicates). *, $p = 0.0119$; ****, $p < 0.0001$

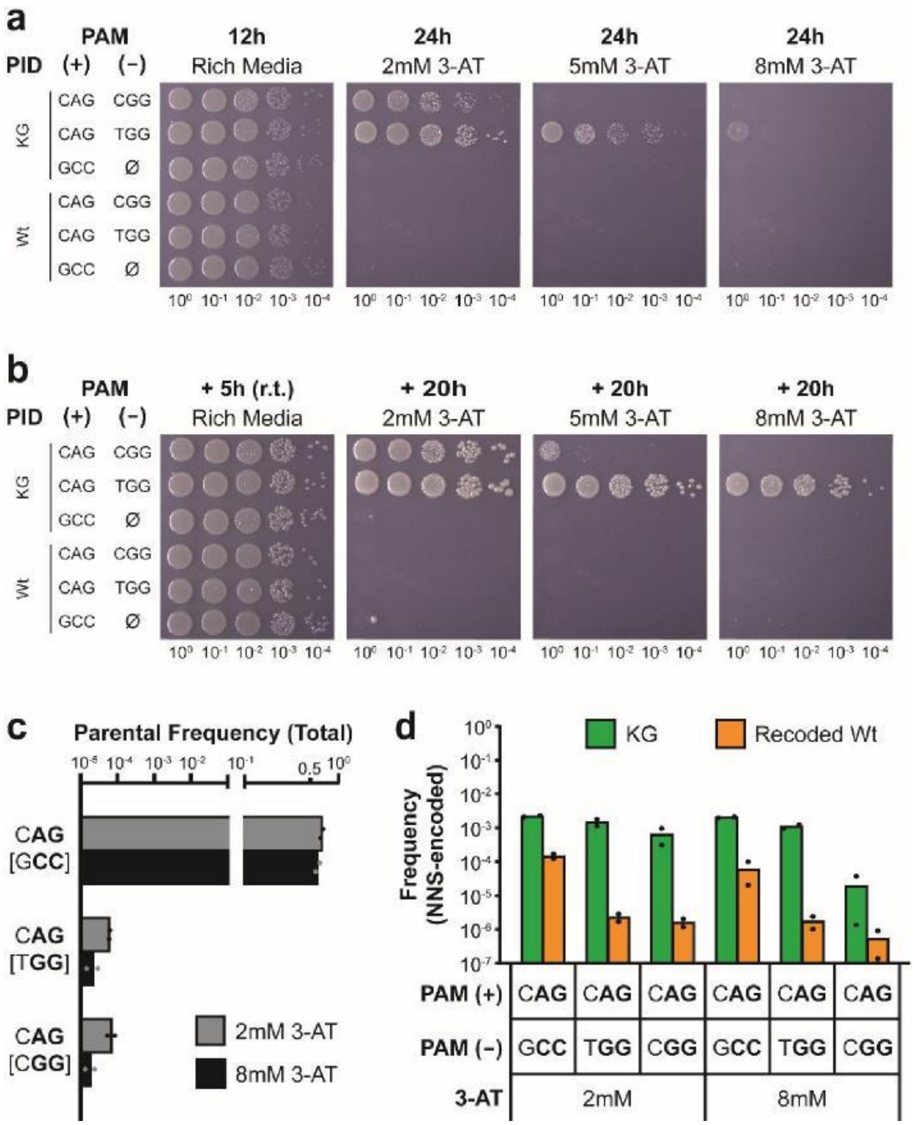

Supplementary Figure S3. Partial preference for CGGCG over TGGAG can constrain evolution of the KG variant in B1H dual-selection assays. (**a**) Comparison of the Wt and KG PID growth phenotypes in clonal ω-dCas9 selection assays with different reporter plasmids. Reporter plasmid PAM sequences at the upstream (+) and downstream (-) protospacer are indicated; Ø denotes absence of the protospacer altogether. Co-transformants were plated as 10-fold serial dilutions on rich media or selective minimal media plates with 2, 5, or 8 mM 3-AT, and incubated at 37 °C for the specified amounts of time. (**b**) The same plates from panel **a**, but after additional incubation times at 37 °C or room temperature (r.t.) as indicated. (**c**) Deep sequencing experiments to determine the frequency of parental Wt sequences out of total PID sequences retrieved in post-selection pools of the round 1 library, with different reporter plasmids and plating on either 2 mM or 8 mM 3-AT. For each experiment, reporter plasmid PAMs are indicated with the counterselection PAM in brackets. *n* = 2, biological replicates (**d**) The same deep sequencing experiments from panel **c**, but re-analyzed to determine

the frequency of KG or recoded Wt sequences out of total NNS-encoded sequences retrieved. *n* = 2, biological replicates

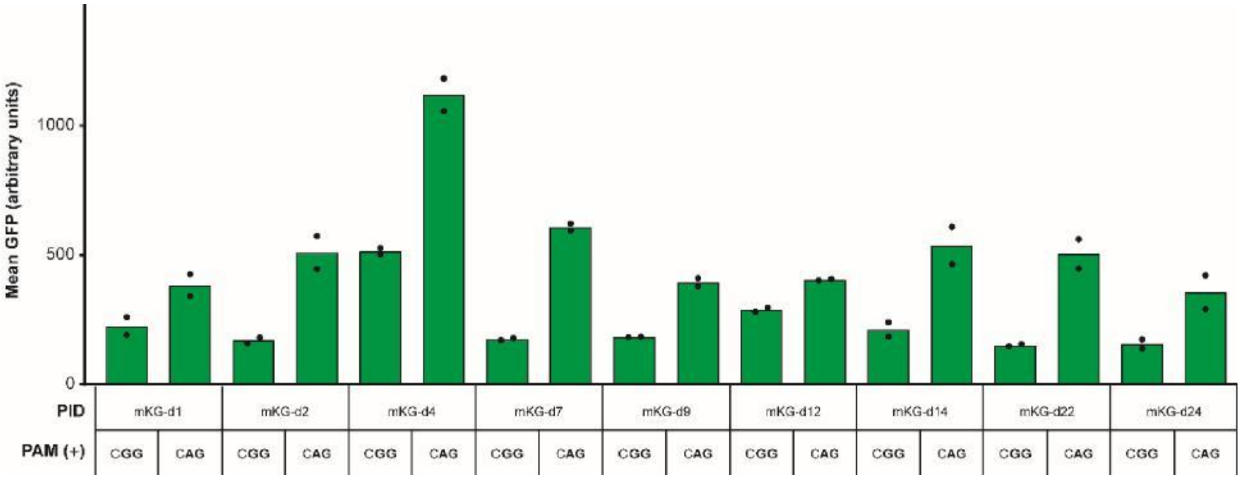

Supplementary Figure S4. Functional testing of nine candidates from the dual-selection experiment in Fig. 3a. GFP fluorescence measured from *E. coli* cells co-transformed with ω-dCas9 plasmids and one of two reporter plasmids containing a single upstream protospacer and PAM as indicated. Isolates are labeled according to their designations in **Fig. 3a**. Mean values were plotted in arbitrary units without normalization. n = 2, biological replicates

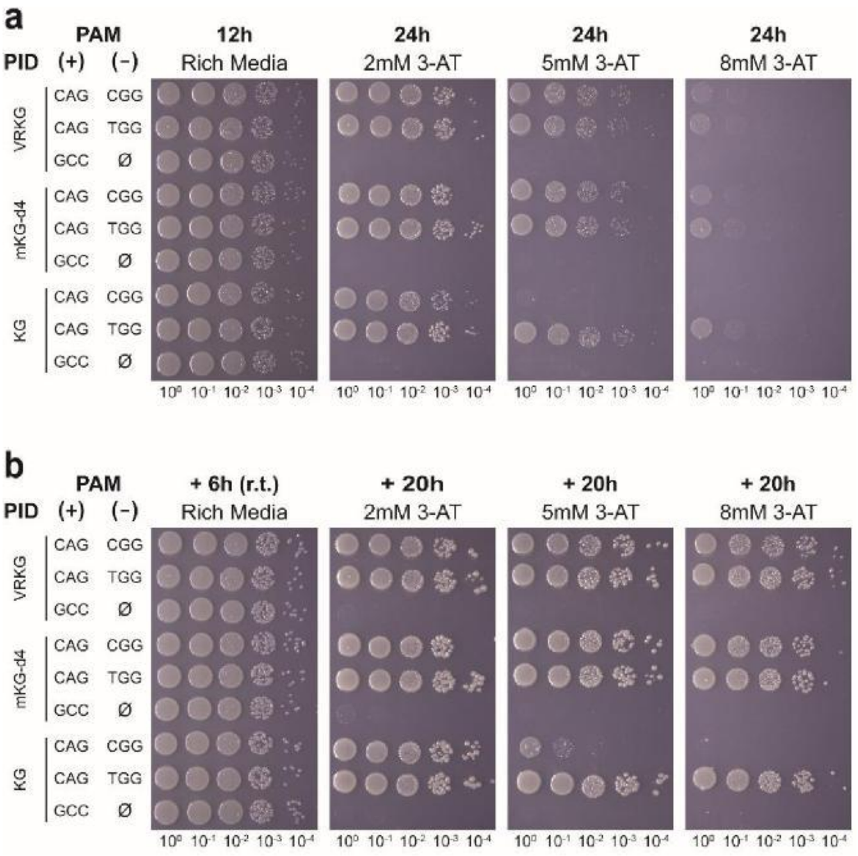

Supplementary Figure S5. The VRKG substitutions are sufficient for bypass of the CGG negative selection PAM in B1H-dependent growth assays. (**a**) Comparison of the KG, VRKG, and mKG-d4 PID growth phenotypes in clonal ω-dCas9 selection assays with different reporter plasmids. Reporter plasmid PAM sequences at the upstream (+) and downstream (-) protospacer are indicated; Ø denotes absence of the protospacer altogether. Co-transformants were plated as 10-fold serial dilutions on rich media or selective minimal media plates with 2, 5, or 8 mM 3-AT, and incubated at 37 °C for the specified amounts of time. (**b**) The same plates from panel **a**, but after additional incubation times at 37 °C or room temperature (r.t.) as indicated.

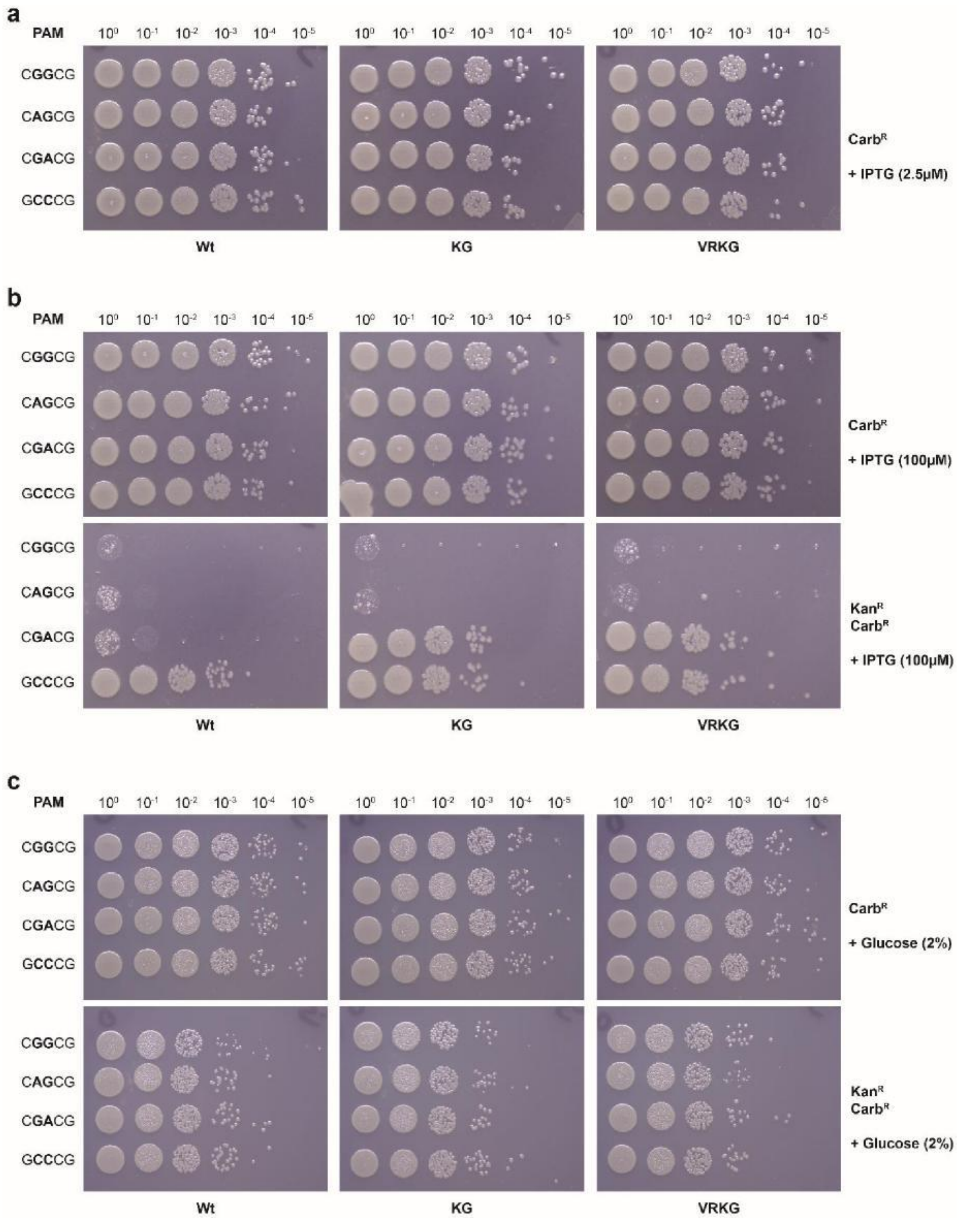

Supplementary Figure S6. Additional plating of the culture dilutions from Fig. 3c with alternative media conditions. (**a**) Control plates containing rich media with IPTG (2.5uM) and carbenicillin but without kanamycin, to select only for Cas9 plasmids. (**b**) Rich media plates containing a maximally inducing concentration of IPTG (100uM) and

either carbenicillin alone (top) or both carbenicillin and kanamycin (bottom). (**c**) Rich media plates containing glucose (2%) to further repress Cas9 expression in the absence of IPTG, and either carbenicillin alone (top) or both carbenicillin and kanamycin (bottom). In all experiments, the carbenicillin-resistant transformants are more numerous than double transformants even under conditions where the target plasmids are not cleaved.

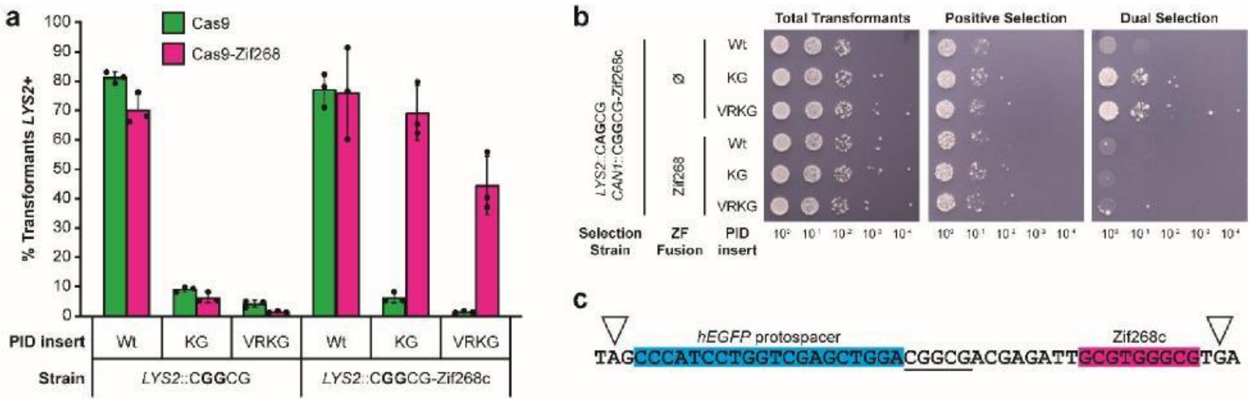

Supplementary Figure S7. Fusion of compensatory ZFs to the KG and VRKG variants can be exploited for increased CGG counterselection stringency in yeast dual-selection strains. (**a**) SSA reporter assays comparing Wt, KG, and VRKG cleavage activities in single-target selection strains with a CGG PAM and either a Zif268c binding site or no ZF binding site at the *LYS2* locus. The Cas9-Zif268 backbone or control Cas9 backbone was co-delivered with a molar excess of clonal PID inserts, and cells were plated in parallel on positive-selection plates and control plates to determine total transformants. The percentage of *LYS2*+ transformants in each replicate was determined from the ratio of positive-selection plate CFUs over control plate CFUs. Error bars, mean ± s.d. (n = 3, biological replicates). (**b**) Testing of the Wt, KG, and VRKG PIDs in a dual-selection strain configured with a CAG PAM at the *LYS2* locus and a CGG PAM with the Zif268c binding site at the *CAN1* locus. The Cas9-Zif268 backbone or control Cas9 backbone with no ZF fusion (Ø) was co-delivered with a molar excess of clonal PID inserts, and cells were plated in parallel as 10-fold serial dilutions on positive-selection plates, dual-selection plates, and control plates to determine total transformants. (**c**) Sequence of the target insertion between direct repeats at the *CAN1* locus in the dual-selection strain from panel **b**. The *hEGFP* protospacer and Zif268c binding site sequences are highlighted in cyan and magenta, with the first 5 bases of the PAM underlined. Stop codons are denoted with triangles as in **Fig. 2a**.

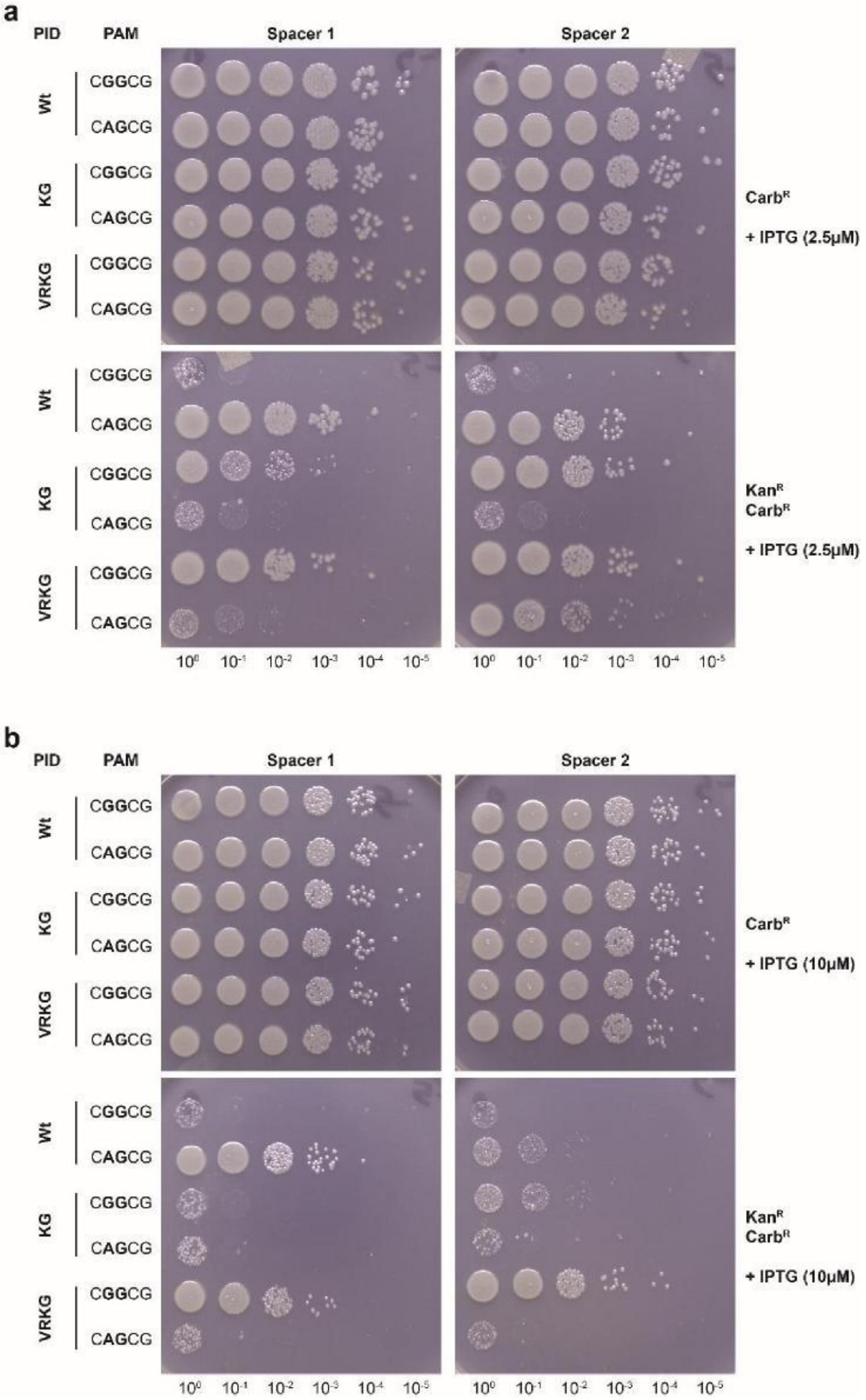

Supplementary Figure S8. Clonal plasmid cleavage assays for initial testing of two spacers targeting the PAM library. (**a**) Wt, KG, or VRKG Cas9 plasmids programmed with one of two spacers in their sgRNA scaffold (Spacer 1 or Spacer 2) were co-transformed with clonal target plasmids harboring the CGG or CAG PAMs adjacent to

corresponding protospacers. Cells were plated in parallel as 10-fold serial dilutions on rich media with a minimally inducing concentration of IPTG (2.5 μM) for Cas9 expression and either carbenicillin alone (top) or carbenicillin and kanamycin to select for both plasmids (bottom). (**b**) The same culture dilutions from panel **a** but plated on media containing 10 μM IPTG. In all experiments, the carbenicillin-resistant transformants are more numerous than double transformants even under conditions where the target plasmids are not cleaved.

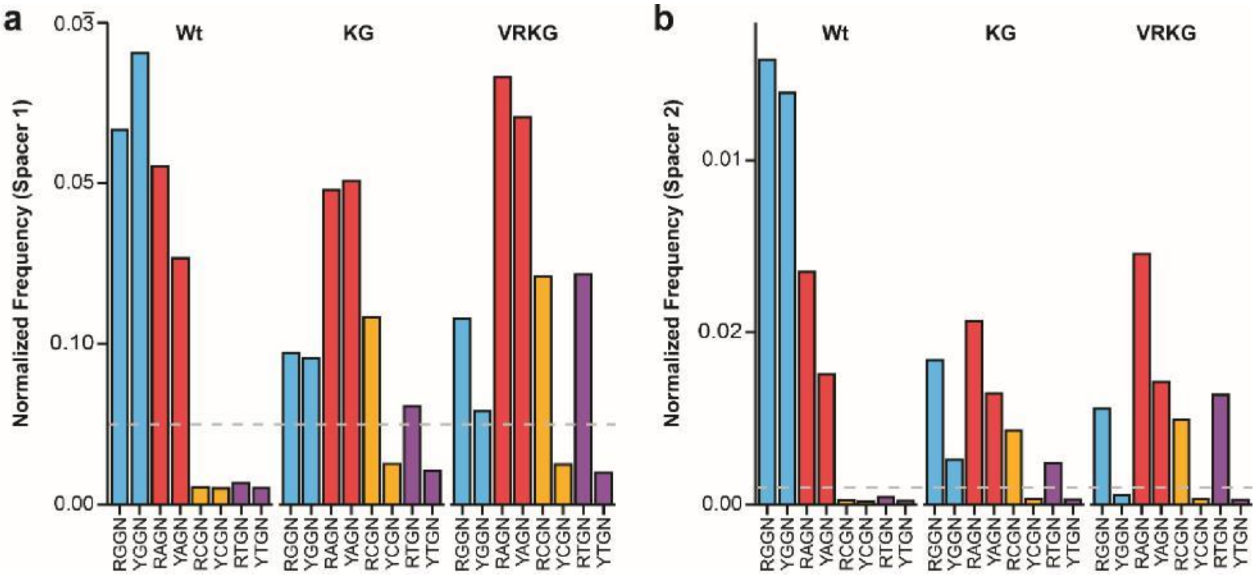

Supplementary Figure S9. Bar plot representation of normalized PAM frequencies from Fig. 4b, re-calculated with an alternative (R/Y)(G/A/C/T)GN binning scheme. (**a**) Normalized PAM frequencies from Wt, KG, and VRKG experiments with Spacer 1 were re-calculated for different PAM bins as indicated. The PAM color coding scheme from Fig. 4 was maintained here. Grey dashed line denotes a normalized frequency value of 0.2 (fivefold depletion). (**b**) PAM-depletion experiments with Spacer 2, re-analyzed and plotted as in panel **a**.

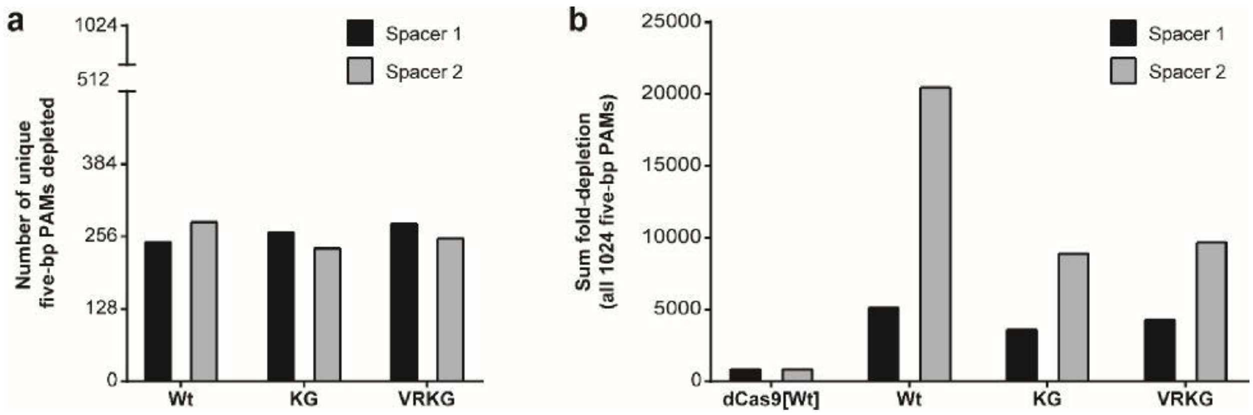

Supplementary Figure S10. PAM repertoires of the KG and VRKG variants are similar to Wt Cas9's in scope but license lower overall activity. (**a**) Quantification of unique five-bp PAMs depleted by the Wt, KG, or VRKG variants with either spacer 1 or spacer 2. Five-bp PAMs were scored as 'depleted' only if their normalized frequency was less than 0.89 because normalized five-bp PAM frequencies measured for the dCas9 control with Wt PID and either spacer were all greater than 0.89 (Supplementary Data 2). (**b**) Summation of fold-depletion values measured for all 1024 five-bp PAMs with either spacer 1 or spacer 2. Background fold-depletion values of 1024.42 (Spacer 1) and 1024.74 (Spacer 2) were obtained for the dCas9 control with Wt PID.

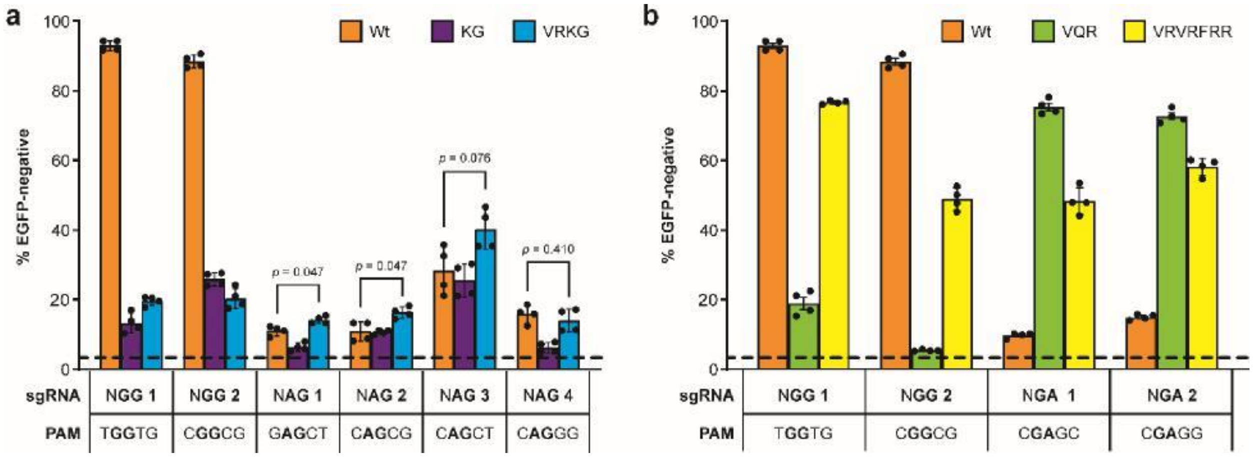

Supplementary Figure S11. Benchmarking of engineered PID variants against Wt Cas9 in a human cell culture assay for EGFP knockdown. EGFP knockdown experiments comparing the KG and VRKG variants with Wt Cas9 on NGG and NAG PAMs as indicated. Knockdown activity was plotted as the percentage of EGFP-negative cells, determined from transiently transfected populations of single cells analyzed by flow cytometry. sgRNA sequences and nomenclature were designed previously[1]. *p* values calculated from two-tailed *t* tests comparing Wt and VRKG with each NAG-targeting sgRNA are shown. Error bars, mean ± s.d. (*n* = 4, biological replicates). Black dashed line denotes the EGFP-negative background level determined from experiments with a non-targeting sgRNA. (**b**) EGFP knockdown experiments plotted as in panel **a**, but comparing the VQR and VRVRFRR variants with Wt Cas9 on NGG and NGA PAMs as indicated. Error bars, mean ± s.d. (*n* = 4, biological replicates). Wt data is re-plotted from panel **a** for ease of comparison.

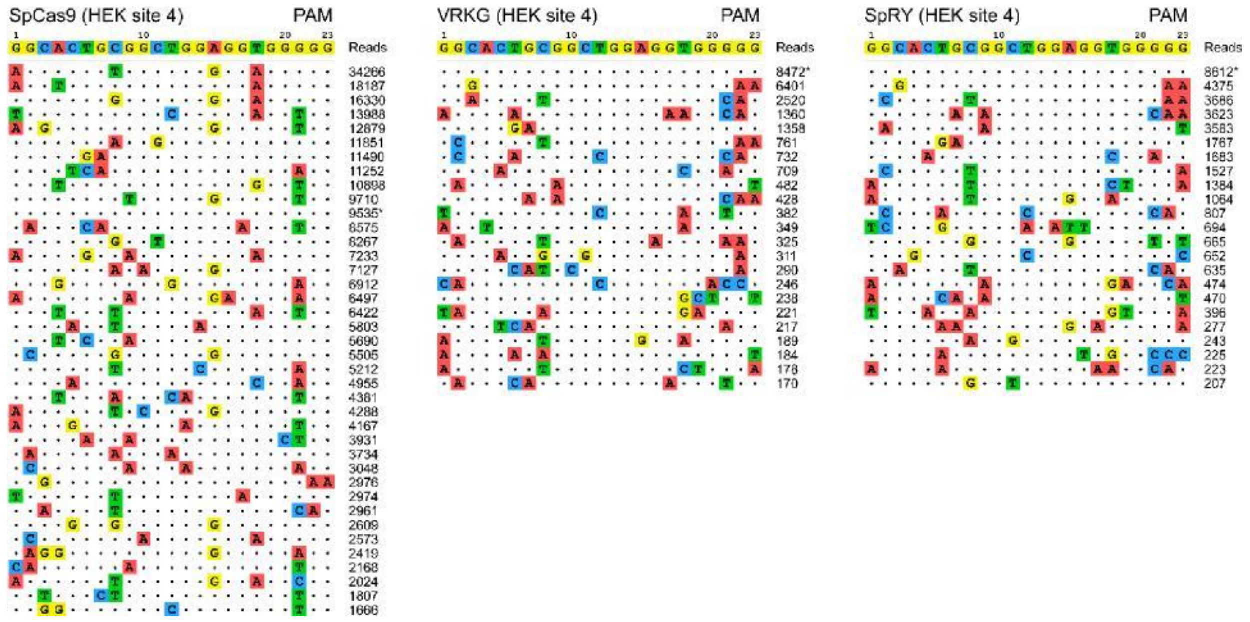

Supplementary Figure S12. Summary of top-ranking GUIDE-seq hits detected for Wt Cas9, VRKG, and SpRY. Target sites contributing over 0.50% of the summed total GUIDE-seq reads obtained for each variant are shown below the expected 'HEK site 4' on-target protospacer and PAM, with mismatches indicated for each off-target sequence. GUIDE-seq read counts measured for the on-target site in each experiment are denoted with an asterisk. The complete lists of off-targets passing GUIDE-seq thresholds in each experiment are presented in **Supplementary Table S7**.

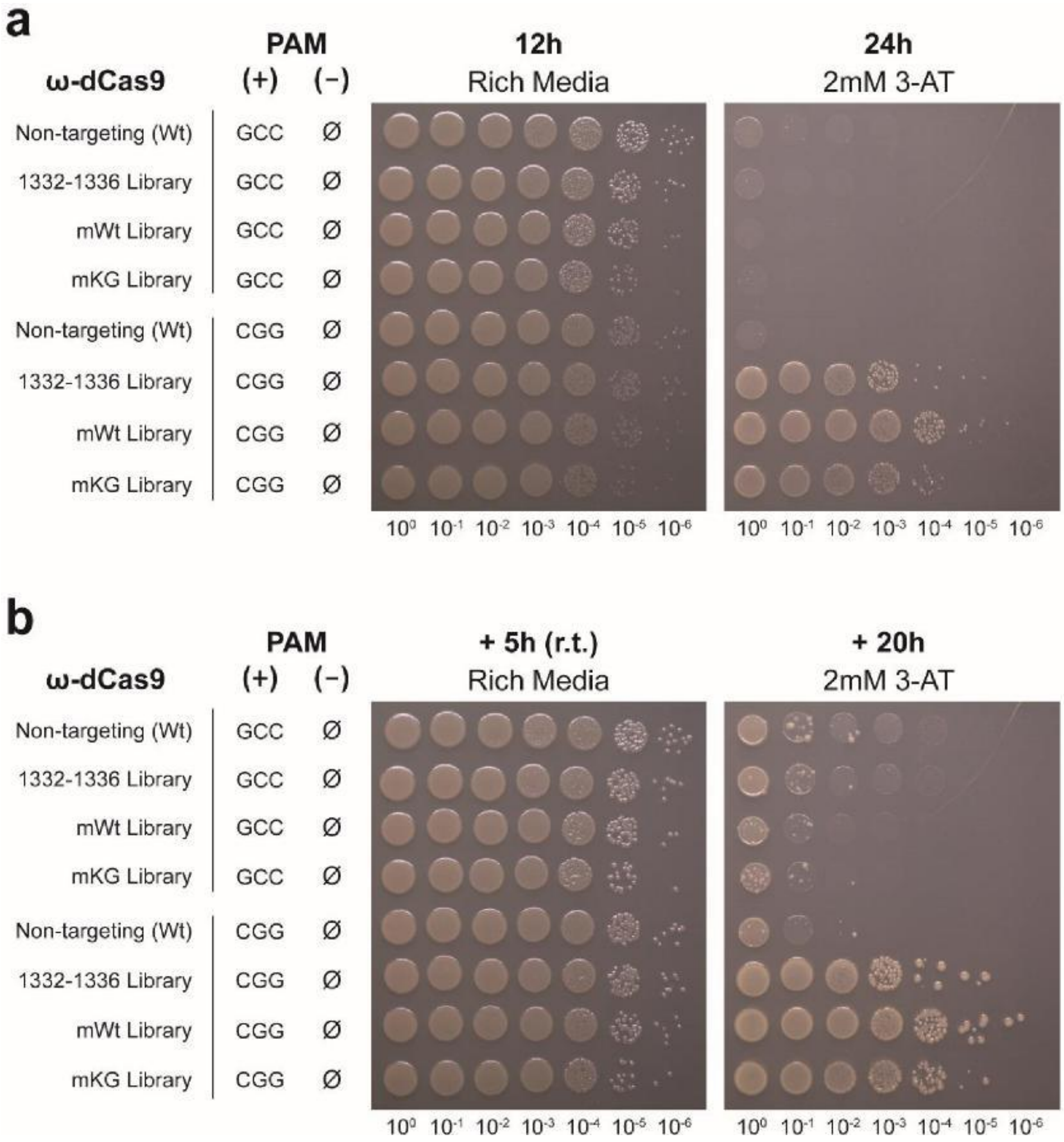

Supplementary Figure S13. ω-dCas9 libraries tested in this work do not encode NCC targeting activity above background. (**a**) Library selection experiments for comparing colony formation after co-delivery of reporter plasmids with one of the three ω-dCas9 plasmid libraries or a clonal non-targeting control plasmid harboring the Wt PID. Reporter plasmid PAM sequences at the upstream (+) and downstream (-) protospacer are indicated; Ø denotes absence of the protospacer altogether. Co-transformants were plated as 10-fold serial dilutions on rich media or selective minimal media plates with 2 mM 3-AT, and incubated at 37 °C for the specified amounts of time. (**b**) The same plates from panel **a**, but after additional incubation times at 37 °C or room temperature (r.t.) as indicated.

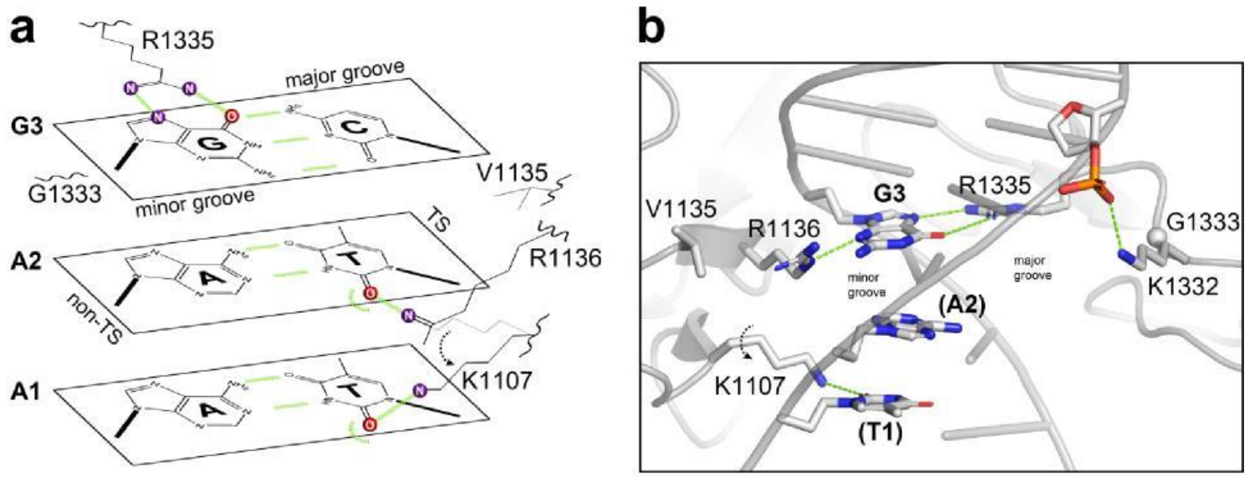

Supplementary Figure S14. Modeling of possible alternative recognition modalities for the VRKG variant's interactions with AAG and ATG PAMs. (**a**) Schematic summary of base-specific recognition via the major and minor groove as in **Fig. 5a**, but for the VRKG variant with an AAG PAM. In principle, R1136's sidechain may adopt an alternative rotamer conformation in range for minor-groove contact with (T2) of the TS if K1107 is concomitantly rotated for contact with (T1). (**b**) Three-dimensional model of the VRKG variant's PID interactions as in **Fig. 5e**, but with an ATG PAM. In this scenario, contact between the (A2) nucleobase and either R1136 or K1107 is disfavored; R1136 instead contacts G3 of the non-TS, whereas K1107 adopts the proposed alternative rotamer conformation for contacting (T1) of the TS.

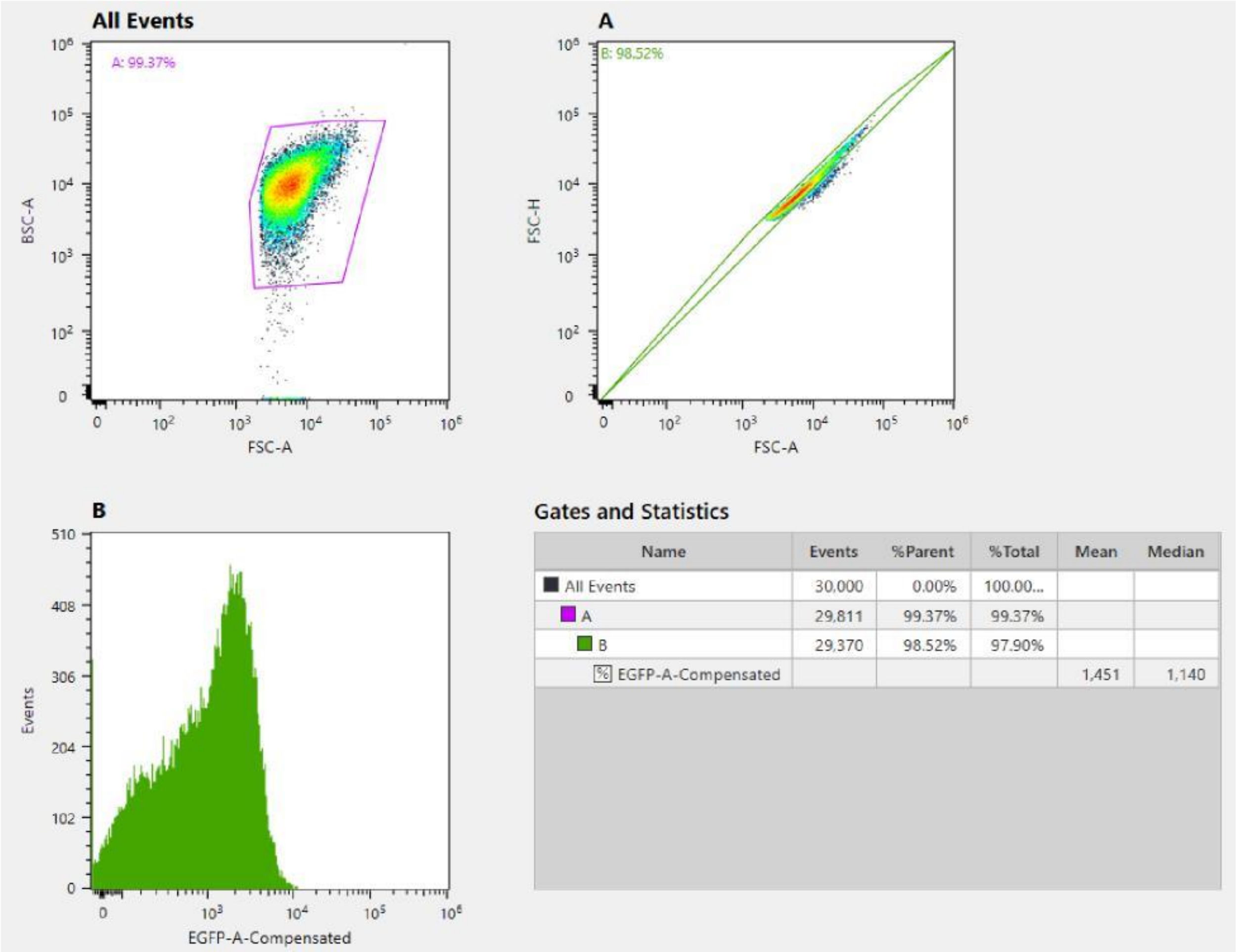

Supplementary Figure S15. Summary of flow cytometry analytical gating strategy used in bacterial fluorescence assays. Pseudo-colored density plot of total events is shown in the upper left, with gate ‘A’ capturing the majority of total events detected based on measurements of forward scatter area (FSC-A) and backscatter area (BSC-A). Density plot in the upper right shows gating of the ‘B’ population from ‘A’, to provide slight enrichment for singlet events based on FSC-A measurements and forward scatter height (FSC-H) measurements. A histogram displaying EGFP-fluorescent events detected from ‘B’ based on EGFP area (EGFP-A) measurements is shown in the bottom left. The mean EGFP fluorescence values, recorded for bar plots in this work, are calculated in arbitrary units on the bottom right. Data presented are from an actual experiment with Wt ω-dCas9 and a CGG PAM reporter plasmid (Fig. 2d); identical gates were applied whenever performing this assay.

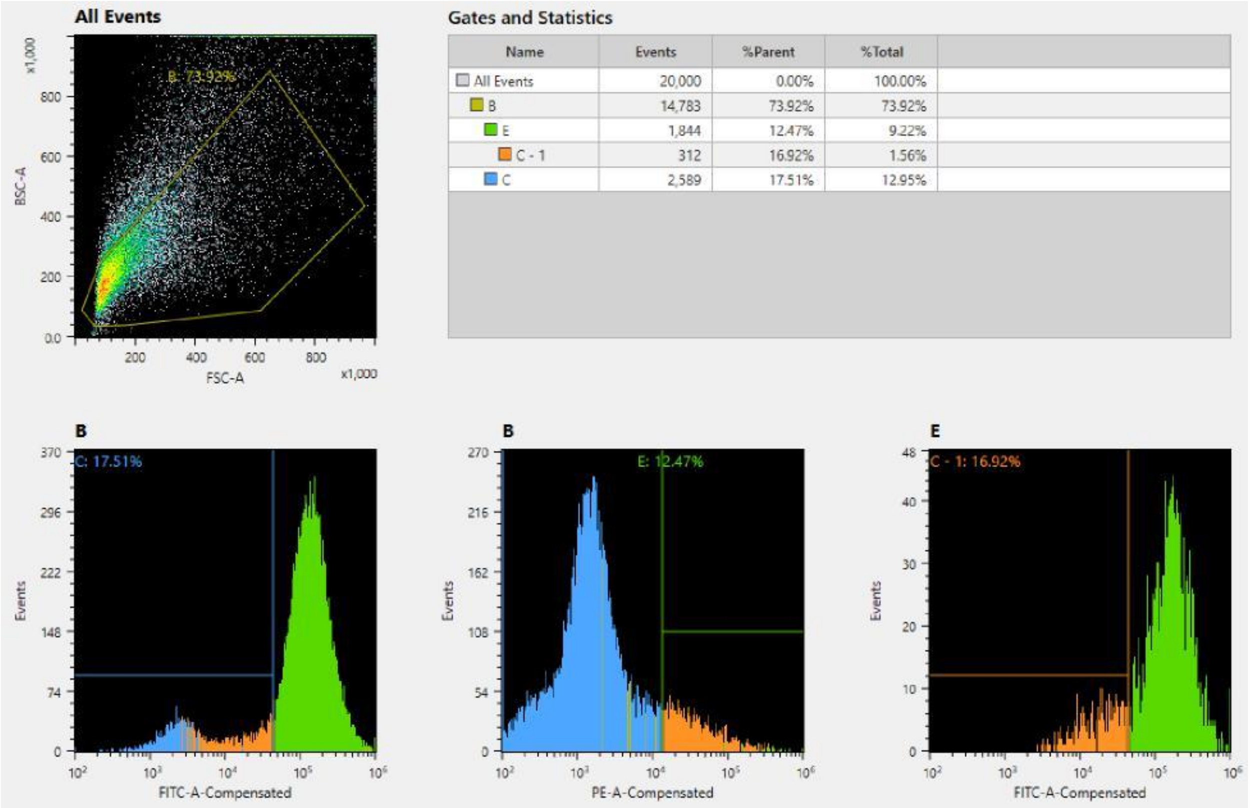

Supplementary Figure S16. Summary of flow cytometry analytical gating strategy used in human cell culture EGFP knockdown assays. Pseudo-colored density plot of total events is shown in the upper left, with an arbitrary 'B' gate intended to capture a majority of cellular events based on measurements of forward scatter area (FSC-A) and backscatter area (BSC-A). Relevant histograms are shown below, with 'E' gated on 'B' to capture the top ~ 50% of tdTomato-fluorescent events based on PE area (PE-A) measurements, and 'C – 1' gated on 'E' to determine the percentage of EGFP-negative events based on FITC area (FITC-A) measurements. The bulk percentage of EGFP-negative events determined directly from 'B' is displayed in the bottom left histogram (gate 'C') for comparison purposes only; all bar plot values were recorded from the 'C – 1' gate on 'E' as shown in the bottom right, and as summarized in the top right. Data presented are from an actual experiment with the VRKG variant and NGG-2 sgRNA (Fig. 5a), and identical gates were applied whenever performing this assay.

Supplementary Table S1. List of PID substitutions identified in the 8 mM 3-AT selection experiments from **Fig. 1d**.

Positive Selection	8mM 3-AT CAGCG [GCCCG]	Dual Selection	8mM 3-AT CAGCG [TGGAG]	Dual Selection	8mM 3-AT CAGCG [CGGCG]
1331 – 1337	Other substitutions	1331 – 1337	Other substitutions	1331 – 1337	Other substitutions
I DRKRY T		I QSYRY T	S1230G	I AAMRI T	
I EHRRY T		I FPMRY T		I QTNRY T	
I PNMRY T		I YASRY T		I DTTRY T	
I DRKRY T		I KSLRM T		I NGRRN T	
I DRKRY T		I RPKRY T	R1114G	I YERRY T	
I NQTRY T		I IGHRY T		I NGHRM T	
I DRKRY T		I KSSRY T		I PANRY T	
I DRKRY T		I AGRRI T		I HPSRY T	
I DRKRY T		I NQYRY T		I CPWRY T	
I DRKRY T		I RAMRY T		I KPTRY T	
I DRKRY T		I KNKRH T		I SPGRY T	
I DRKRY T		I RTRRY T		I MGNRY T	
I DRKRY T	V1149A	I TPKRE T		I AACRY T	
I QGGRY T		I GASRF T		I RGRRF T	
I DRKRY T		I VTRRY T		I PCKRH T	
I RGRRH T		I KGDRY T		I SERRY T	
I DRKRY T		I PANRH T		I MNKRY T	
I DRKRY T		I CADRY T		I NPRRH T	
I KPLRL T		I TARRN T		I SPNRY T	
I DRKRY T		I GKRRN T		I LPYRH T	
I CNNRY T		I DTCRY T		I PPRRT T	
I IGQRY T		I ESMRY T			
I DRKRY T		I DKTRM T			
I DRKRY T		I TNVRY T			

The reporter plasmid PAM configurations used for these experiments are displayed at the top of each column with the counterselection PAM sequence in brackets. Non-synonymous substitutions relative to Wt are shown with red lettering.

Supplementary Table S2. List of PID substitutions identified in 5 mM 3-AT dual-selection experiments with the site-saturation library tested in **Fig. 1d.**

Dual Selection	5mM 3-AT CAGCG [TGGAG]	Dual Selection	5mM 3-AT CAGCG [CGGCG]
1331 – 1337	Other substitutions	1331 – 1337	Other substitutions
I KGKRY T		I EPRRY T	
I NTRRY T		I LTRRY T	
I PSRRY T		I PPKRN T	
I RAMRY T		I GPNRY T	
I IKKRY T		I ETKRY T	
I MYRRY T		I IAKRY T	
I HPKRM T		I VGRRY T	
I QQRRY T		I LATRY T	
I SATRY T		I DCKRY T	
I RPCRY T		I APYRM T	
I DPCRY T		I DAKRY T	E1275K
I QGHRH T		I GPFRM T	
I QARRY T		I KPVRY T	
I PGKRM T		I YSRRY T	
I TGTRY T		I YSKRY T	
I PGYRY T		I SSFRY T	
I PGARY T		I ETKRY T	
I NAFRY T		I SNQRY T	
I ASRRY T		I DVYRY T	
I SARRY T		I HPKRM T	
I LNKRY T		I PPARY T	
I KPRRY T		I KAFRY T	
I QGYRY T			

The reporter plasmid PAM configurations used for these experiments are displayed at the top of each column with the counterselection PAM sequence in brackets. Non-synonymous substitutions relative to Wt are shown with red lettering.

Supplementary Table S3. List of PID substitutions identified in *S. cerevisiae* selection experiments from **Fig. 2c.**

Positive Selection:	*LYS2+* (14/58)		Dual Selection:	*LYS2+*, Can^{R} (19/19)	
Can^{S}	1331 – 1337	Other substitutions	Can^{S}	1331 – 1337	Other substitutions
*	I KGKRY T			I KGKRY T	
	I RAQRL T			I TTLRM T	
*	I LRCRL T			I KGKRY T	
	I DVTRM T			I KGKRY T	Q1091P
	I RKKRY T	Q1091P		I KGKRY T	
	I RAARY T			I RSIRH T	
	I VGLRN T			I VGKRV T	
*	I ARKRY T			I FIYRY T	Q1091P
	I KGKRY T			I SKHRI T	Q1091P
	I RVSRK T		n.a.	I KKKRN T	
	I SYYRY T			I PAKRT T	
*	I SRCRN T			I KGKRY T	
	I KGKRY T			I KGKRY T	
	I GGRRY T			I GARRM T	
				I SASRY T	Q1091P
				I KPHRK T	
				I IIRRY T	
				I KVRRL T	
				I KGKRY T	

The non-Wt fraction of total sequences recovered from each experiment is denoted in parentheses above each column. Non-synonymous substitutions relative to Wt are shown with red lettering; parental Wt sequences recovered in the positive selection experiment are not listed. The 14 non-Wt positive selection isolates were scored for canavanine resistance by patching on a dual-selection plate; canavanine-sensitive (Can^{S}) isolates are marked with an asterisk. Q1091P substitutions likely resulted from the backbone used for assemblies and were not studied further (see Methods).

Supplementary Table S4. List of PID substitutions identified in a dual-selection ω-dCas9 experiment with random mutagenesis in the Wt PID.

Isolate	Dual Selection: CA<u>G</u>CG [C<u>GG</u>CG]
mWt-d1	K1231I,E1275K,Q1305K,R1333P
mWt-d3	S1106F,L1206F,L1291H,R1333P
mWt-d4	Y1232H,E1304G,R1333C
mWt-d6	F1181L,H1241L,R1333C
mWt-d7	L1164R,E1189D,L1194S,K1231Q,L1236I,Q1254R,A1285T,R1333C
mWt-d8	L1228P,K1296I,R1333C
mWt-d9	Q1221H,K1300I,R1333C
mWt-d10	P1229S,E1275G,K1325T,T1330A,R1333C
mWt-d11	A1147G,R1171S,E1225G,V1259A,Q1305L,R1333P
mWt-d12	D1135V,K1161I,I1196V,Q1221H,L1238F,L1245S,L1318I,K1325R,R1333S
mWt-d13	E1243G,Q1272H,R1333C
mWt-d15	S1172G,H1264R,F1313L,R1333P
mWt-d16	H1241R,G1247S,E1250K,E1271V,D1288N,K1325R,R1333C
mWt-d17	S1159Y,F1174I,Y1201F,G1218R,R1333P
mWt-d18	R1333P,S1338F
mWt-d19	M1213L,R1333C
mWt-d20	L1157F;E1225V;K1255E;P1301S;R1333C
mWt-d21	S1230C;R1333C
mWt-d22	S1240G;Q1254P;E1304K;L1315S;R1333C
mWt-d23	M1169T;E1170D;S1173P;K1191R;K1278E;Y1294C;T1314S;R1333P
mWt-d24	D1117E;K1156R;L1226Q;R1298S;R1333C

The reporter plasmid's PAM configuration is displayed above with the counterselection PAM in brackets. Orange-colored lettering was used to emphasize various substitutions detected at R1333.

Supplementary Table S5. List of PID substitutions identified in positive-selection ω-dCas9 experiments with random mutagenesis in the Wt or KG PIDs.

Isolate	Positive Selection: CAGCG [GCCCG]
mWt-p1	S1248G,K1263E,K1289T,S1292G,P1321H
mWt-p2	H1311Q,D1328N,T1329S,R1333S
mWt-p3	N1093S
mWt-p4	E1150V,Q1221H,E1243D
mWt-p5	K1278R
mWt-p6	no PID substitutions
mWt-p7	K1118R,D1135N,K1231E,E1243V,R1333H
mWt-p8	L1206F,K1296R
mWt-p9	V1149A,K1192N,S1240G,K1246M,F1324S
mWt-p10	N1252S,S1338A
mWt-p11	K1124N,V1143A
mWt-p12	K1130N,M1213L,L1318R,D1332V
mWt-p13	I1195V
mWt-p14	R1333S
mWt-p15	M1213V,H1262R
mWt-p16	I1179T,K1188M,L1245F,P1321H
mWt-p17	T1102S,Y1242C,R1333S
mWt-p18	no PID substitutions
mWt-p19	E1253G,K1263R,H1349R
mWt-p20	Q1272R
mWt-p21	K1246M,S1274C,A1293S,Q1305H,L1315M
mWt-p22	A1320V
mWt-p23	F1134Y,A1320V
mWt-p24	V1143A,Q1272L
mKG-p1	V1290A,K1300E
mKG-p2	L1157V,P1178S,D1267G,K1289E
mKG-p3	G1218E,K1231N,E1253V,K1296Q,Q1305L
mKG-p4	V1100E,K1129I,K1156R
mKG-p5	F1235L,Q1305R
mKG-p6	K1255Q,K1332R
mKG-p7	K1113R,N1208Y,E1250V,K1296N
mKG-p8	K1161E,K1185I,E1250G,E1304V,F1327Y
mKG-p9	A1121G,S1230C
mKG-p10	K1197R,K1231E,Y1242C,S1248G,K1296N,A1306V
mKG-p11	V1146D,D1180V,F1276Y,N1295I
mKG-p12	K1300R,I1331T
mKG-p13	T1102S
mKG-p14	D1180V,P1229T,L1236I,P1249S,K1278R,D1284E,T1337S
mKG-p15	K1153N,E1268G
mKG-p16	N1295I
mKG-p17	Y1187C,S1248R
mKG-p18	Q1272R
mKG-p19	K1263M,D1284G,D1328N
mKG-p20	no PID substitutions
mKG-p21	P1229T,K1244R,D1251E
mKG-p22	F1258L
mKG-p23	E1150V,N1208S
mKG-p25	L1226Q,D1267Y,S1292R,K1296R,L1312I,A1322S

The reporter plasmid's PAM configuration is displayed above with the counterselection PAM in brackets. Teal-colored lettering was used to emphasize various substitutions detected at R1333 or K1296 in a few of the isolates. As indicated, mutagenized Wt isolates are listed in the top portion of the table, whereas mutagenized KG isolates are listed in the bottom portion.

Supplementary Table S6. List of additional non-synonymous PID substitutions identified in VRKG library experiments with the selection strain from Supplementary **Fig. S7b.**

Isolate	Dual Selection: *LYS2+*, Can^R
mVRKG-d2	F1105L,Y1242C,Q1261R,F1327Y,E1341G
mVRKG-d3	premature stop at K1158
mVRKG-d4	K1113E,K1246M,S1292G,I1310V,A1323T,F1327L
mVRKG-d5	V1233L,K1332M
mVRKG-d7	K1197R,K1244E,N1286I,K1296E,G1333E
mVRKG-d8	frameshift at S1363
mVRKG-d9	I1195T,N1208S,A1217V,N1295S
mVRKG-d11	I1302K,P1321H,D1328N,K1340R
mVRKG-d12	L1119H,P1178S,K1278E,N1295S,T1330S

Isolate	Positive Selection: *LYS2+*	Can^S
mVRKG-p1	N1115D,N1252S,N1286S,I1309V	*
mVRKG-p2	L1245S,F1258L,S1292R,T1316S	*
mVRKG-p3	H1264R	*
mVRKG-p4	no PID substitutions	*
mVRKG-p5	R1136G,E1341G	*
mVRKG-p6	no PID substitutions	*
mVRKG-p7	L1266F,Q1272L	*
mVRKG-p8	M1213I,E1271G,D1284N,I1331V	*
mVRKG-p9	F1258Y,Q1261L	*
mVRKG-p10	Q1256R	*
mVRKG-p11	L1236S,Y1242C,K1255E	*
mVRKG-p12	no PID substitutions	*
mVRKG-p13	N1208D,V1233E	*
mVRKG-p14	no PID substitutions	*
mVRKG-p15	no PID substitutions	*
mVRKG-p16	K1153R,E1183K,L1194S,E1225G,K1289N	*
mVRKG-p17	S1172G	*
mVRKG-p18	S1154L	*
mVRKG-p19	E1150V,V1160D,R1298S	*
mVRKG-p20	frameshift at Q1305	
mVRKG-p21	L1226Q,N1295I,D1299G,S1363Y	*
mVRKG-p22	Q1272R,S1292G,F1324S	*
mVRKG-p23	M1213V,S1292G	*
mVRKG-p24	no PID substitutions	*
mVRKG-p25	D1180N	*
mVRKG-p26	F1134L,L1198P	*
mVRKG-p27	E1253D,N1317S	*
mVRKG-p28	Y1141H,S1248G,Y1336H	*
mVRKG-p29	K1156E	*
mVRKG-p30	R1114G	*

As indicated, dual-selection isolates are listed in the top portion of the table and positive-selection isolates are listed in the bottom portion. The 30 positive-selection isolates were scored for canavanine resistance by patching on a dual-selection plate; canavanine-sensitive (Can^S) isolates are marked with an asterisk.

Supplementary References

1 Kleinstiver, B. P. *et al.* Engineered CRISPR-Cas9 nucleases with altered PAM specificities. *Nature* **523**, 481-485 (2015).

2 Oakes, B. L. *et al.* Multi-reporter selection for the design of active and more specific zinc-finger nucleases for genome editing. *Nat Commun* **7**, 10194 (2016).

3 Brachmann, C. B. *et al.* Designer deletion strains derived from Saccharomyces cerevisiae S288C: a useful set of strains and plasmids for PCR-mediated gene disruption and other applications. *Yeast* **14**, 115-132 (1998)
