# Supplementary material for: Engineered dual selection for directed evolution of SpCas9 PAM specificity
Source: Nat Commun. 2021 Jan 13;12:349. doi: 10.1038/s41467-020-20650-x (PMC7807044; doi:10.1038/s41467-020-20650-x)
Supplement: Supplementary file 3 — Description of Additional Supplementary Files [file 41467_2020_20650_MOESM3_ESM.pdf]

Title: Supplementary Data 1
Description: PAM tallies from processed reads and normalized post-depletion PAM frequencies associated with Figure 4
Data extracted from each of the two PAM bins analyzed for Figure 4 (i.e., "Position 2 through 4" or "Position 1 through 3") are provided on separate sheets in the file. For each of the indicated PAMs, post-depletion normalized frequencies were calculated based on tallies of the spacer-specific PAM counts and the total number of PAMs detected in the corresponding post-depletion sample and pre-depletion PAM library (see Methods for more details). Numerical values are provided in each column as indicated.

Title: Supplementary Data 2
Description: PAM tallies from processed reads and normalized post-depletion PAM frequencies associated with Supplementary Figure S10
For each five-bp PAM, post-depletion normalized frequencies were calculated based on tallies of the spacer-specific PAM counts and the total number of PAMs detected in the corresponding post-depletion sample and pre-depletion PAM library (see Methods for more details). Numerical values are provided in each column as indicated.

Title: Supplementary Data 3
Description: Complete summary of GUIDE-seq hits detected for Wt Cas9, VRKG, and SpRY with the 'HEK site 4' sgRNA
Data for each variant are provided on separate sheets in the file, with the details for each off-target hit (or the on-target hit with zero mismatches) provided in various columns as indicated. Processed reads mapping to each genomic site's negative or positive strand, respectively, are tallied in the "- Reads" and "+ Reads" columns which sum to "Total Reads". Columns labelled "- ODN Reads" and "+ ODN Reads" tally processed read counts originating from the double-stranded oligodeoxynucleotide (dsODN) tag's negative or positive strand, respectively. Browser Extensible Data (BED) formatting was used in genomic analyses.

Title: Supplementary Data 4
Description: Plasmid DNA sequences and donor DNA sequences used during strain construction
The DNA identifier, expected sequence, and brief description is provided for each entry in separate columns as indicated. Entries corresponding to plasmid DNA (assumed circular) are denoted as such in their description or otherwise using an identifier that begins with a lowercase 'p'.

Title: Supplementary Data 5
Description: Sequences of commercially synthesized ssDNA oligonucleotides or linear dsDNA fragments
The identifier, ssDNA or dsDNA sequence ordered, and purpose(s) in this study is provided for each entry in separate columns as indicated. Entries containing identifiers that begin with a 'gGG' prefix correspond to dsDNA fragments; all other entries correspond to oligonucleotides.

Title: Supplementary Data 6
Description: List of yeast strains used in this work
The strain identifier, expected genotype, and brief description is provided for each entry in separate columns as indicated.
