# Supplementary material for: Engineered dual selection for directed evolution of SpCas9 PAM specificity
Source: Nat Commun. 2021 Jan 13;12:349. doi: 10.1038/s41467-020-20650-x (PMC7807044; doi:10.1038/s41467-020-20650-x)
Supplement: Supplementary file 10 — Reporting Summary [file 41467_2020_20650_MOESM10_ESM.pdf]

nature research

Corresponding author(s): Marcus B. Noyes
Gregory W. Goldberg

Last updated by author(s): 11/13/2020

Reporting Summary

Nature Research wishes to improve the reproducibility of the work that we publish. This form provides structure for consistency and transparency in reporting. For further information on Nature Research policies, see Authors & Referees and the Editorial Policy Checklist .

Statistics

For all statistical analyses, confirm that the following items are present in the figure legend, table legend, main text, or Methods section.

n/a	Confirmed	
☐	☒	The exact sample size (*n*) for each experimental group/condition, given as a discrete number and unit of measurement
☐	☒	A statement on whether measurements were taken from distinct samples or whether the same sample was measured repeatedly
☐	☒	The statistical test(s) used AND whether they are one- or two-sided<br>*Only common tests should be described solely by name; describe more complex techniques in the Methods section.*
☒	☐	A description of all covariates tested
☐	☒	A description of any assumptions or corrections, such as tests of normality and adjustment for multiple comparisons
☐	☒	A full description of the statistical parameters including central tendency (e.g. means) or other basic estimates (e.g. regression coefficient) AND variation (e.g. standard deviation) or associated estimates of uncertainty (e.g. confidence intervals)
☐	☒	For null hypothesis testing, the test statistic (e.g. *F*, *t*, *r*) with confidence intervals, effect sizes, degrees of freedom and *P* value noted<br>*Give P values as exact values whenever suitable.*
☒	☐	For Bayesian analysis, information on the choice of priors and Markov chain Monte Carlo settings
☒	☐	For hierarchical and complex designs, identification of the appropriate level for tests and full reporting of outcomes
☒	☐	Estimates of effect sizes (e.g. Cohen's *d*, Pearson's *r*), indicating how they were calculated

Our web collection on statistics for biologists contains articles on many of the points above.

Software and code

Policy information about availability of computer code

Data collection	Raw deep sequencing data was collected using Illumina NextSeq 500 or MiSeq instruments. Raw flow cytometry data was collected using a SONY SH800 cell cytometer. Raw attenuance measurements from bacterial cultures grown in 96-well plates were collected using a Synergy H1 microplate reader (BioTek).
Data analysis	After demultiplexing of dual-indexed raw sequencing data from NextSeq 500 runs, paired-end reads were analyzed with UNIX commands and packages (including PEAR v0.9.11 and EMBOSS Transeq v6.6) and custom python (v3.6) or R (v3.5) scripts as described in the Methods. Raw flow cytometry data was analyzed using commercial software provided with the SONY SH800 instrument. All quantitative data was analyzed and plotted using Microsoft Excel 2016 MSO (16.0.13231.20250) 64-bit, GraphPad Prism 8, or ggplot2 (v3.1.0) for R. PyMOL (v2.2) was used for all structural analyses and molecular modeling.

For manuscripts utilizing custom algorithms or software that are central to the research but not yet described in published literature, software must be made available to editors/reviewers. We strongly encourage code deposition in a community repository (e.g. GitHub). See the Nature Research guidelines for submitting code & software for further information.

Data

Policy information about availability of data

All manuscripts must include a data availability statement. This statement should provide the following information, where applicable:

- Accession codes, unique identifiers, or web links for publicly available datasets
- A list of figures that have associated raw data
- A description of any restrictions on data availability

Relevant data supporting the findings of this study will be made available in the published article and its Supplementary Information files, the Sequence Read Archive of the NCBI (BioProject accession codes: PRJNA672767, PRJNA673046, and PRJNA673206), and from the corresponding authors upon reasonable request.

Field-specific reporting

Please select the one below that is the best fit for your research. If you are not sure, read the appropriate sections before making your selection.

☒ Life sciences ☐ Behavioural & social sciences ☐ Ecological, evolutionary & environmental sciences

For a reference copy of the document with all sections, see nature.com/documents/nr-reporting-summary-flat.pdf

Life sciences study design

All studies must disclose on these points even when the disclosure is negative.

Sample size	Statistical testing to predetermine experimental sample sizes was not performed in this work; sample sizes were chosen based on existing procedures and standards in the field.
Data exclusions	Sequences from screened library isolates yielding incomplete or poor-quality Sanger sequencing traces that could not be unambiguously aligned and translated in the region of interest were not presented in this work and were excluded from our analyses. GUIDE-seq data analyses adhered to previously established read exclusion criteria; all other deep sequencing analyses considered only reads that could be retrieved with perfectly matching sequences flanking the region of interest because the experiments were designed to quantify sequence differences only within the region of interest (on genetic backgrounds that were otherwise isogenic).
Replication	GUIDE-seq data were generated from single biological replicates. PAM-depletion data were also generated from single biological replicates but each variant was tested in two independent experiments with different spacers (one biological replicate for each variant-spacer combination). All other assays were conducted with a minimum of two biological replicates for each experiment, and results were reliably reproduced under the conditions tested.
Randomization	Randomization was generally not relevant to our study because our experiments did not involve human or animal subjects, and because most experiments were designed to test genetic variables on otherwise-isogenic backgrounds; all samples could be treated equivalently in such cases. In other cases where aliquot samples were withdrawn from a single starting population (clonal or library pool) for treatment under different downstream conditions, pipetting of equivalent volumes from a well-mixed liquid starting population effectively allowed for random sampling.
Blinding	Investigators were not blinded during data collection because the experiments did not involve human or animal subjects and were readily controlled without blinding. Investigators were not blinded during data analyses because the key findings are supported by quantitative measurements (with statistical testing where relevant) that do not rely heavily on subjective judgment for interpretation.

Reporting for specific materials, systems and methods

We require information from authors about some types of materials, experimental systems and methods used in many studies. Here, indicate whether each material, system or method listed is relevant to your study. If you are not sure if a list item applies to your research, read the appropriate section before selecting a response.

Materials & experimental systems

n/a	Involved in the study	
☒	☐	Antibodies
☐	☒	Eukaryotic cell lines
☒	☐	Palaeontology
☒	☐	Animals and other organisms
☒	☐	Human research participants
☒	☐	Clinical data

Methods

n/a	Involved in the study	
☒	☐	ChIP-seq
☐	☒	Flow cytometry
☒	☐	MRI-based neuroimaging

Eukaryotic cell lines

Policy information about cell lines

Cell line source(s)	The U2OS.EGFP human cell line was received from the Joung lab, Mass. General Hospital.
Authentication	STR profiling by ATCC and deep sequencing, performed previously.
Mycoplasma contamination	Cell lines were previously confirmed negative for Mycoplasma contamination, but were not re-tested during the course of this study.
Commonly misidentified lines (See ICLAC register)	No commonly misidentified cell lines were used in this work.

Flow Cytometry

Plots

Confirm that:

- [x] The axis labels state the marker and fluorochrome used (e.g. CD4-FITC).
- [x] The axis scales are clearly visible. Include numbers along axes only for bottom left plot of group (a 'group' is an analysis of identical markers).
- [x] All plots are contour plots with outliers or pseudocolor plots.
- [x] A numerical value for number of cells or percentage (with statistics) is provided.

Methodology

Sample preparation	Bacterial samples were aliquoted from minimal media cultures, and human cell culture samples were trypsinized and aliquoted from culture dishes 52 h post-nucleofection; see relevant Methods sections for more details.
Instrument	SONY SH800 cell cytometer
Software	Commercial software provided with the SONY SH800 instrument.
Cell population abundance	30,000 events were analyzed per sample in bacterial experiments; 20,000 events were analyzed per sample in human cell culture experiments.
Gating strategy	Bacterial gating was pre-calibrated using single-color controls from the same strain background, and human cell culture gating was determined using untransfected or tdTomato-transfected controls; see relevant Methods sections for more details.

- [x] Tick this box to confirm that a figure exemplifying the gating strategy is provided in the Supplementary Information.
