# Supplementary material for: Engineered dual selection for directed evolution of SpCas9 PAM specificity
Source: Nat Commun. 2021 Jan 13;12:349. doi: 10.1038/s41467-020-20650-x (PMC7807044; doi:10.1038/s41467-020-20650-x)
Supplement: Supplementary file 2 — Peer Review File [file 41467_2020_20650_MOESM2_ESM.pdf]

Reviewers' Comments:

Reviewer #1:
Remarks to the Author:
This paper describes two novel techniques for doing simultaneous positive and negative selection for proteins with desired DNA-binding specificity. One method is performed in bacteria and is an extension of the bacterial one-hybrid method for which expression of an essential is under both positive and negative control. The other method utilizes yeast and selects for functional cleavage activity of the variant Cas9 proteins, again both positively and negatively. Current approaches use sequential, rather than simultaneous, positive and negative selections and the authors point out that there are some advantages, besides just being faster. The two different systems generally give the same results, but there may be advantages to one or the other for specific problems. They utilize these methods to obtain novel variants of the Cas9 protein that alters the PAM specificity, now preferring an NAG sequence while discriminating against the normal NGG sequence. Additional rounds of mutation and selection identified a 4 amino acid variant, VRKG, with increased specificity for NAG over YGG sites but with a surprising affinity for RNG sequences. They follow this up with additional experiments and computational modeling to try to explain the observed preferences, which is partially successful. They also try adding a zinc finger domain to the Cas9 protein because it increases the specificity in vivo for sites that also contain the ZF binding site. However this did not lead to improvements in the dual selection strategy and was not pursued further.
The two strategies presented in this paper are clever ways to do both and negative selection simultaneously. Besides speeding up the process this provides a different selection pressure that may lead to a different exploration of the sequence/function space and may lead to different solutions than the standard sequential selection methods. They didn't do a comparison so we don't know if that is true. I think the method will be useful for people doing selections for DNA binding proteins with altered specificity, not just Cas9 variants. The paper is well written and contains several additional and interesting analyses that I didn't describe above. The modeling part is a bit hand-wavy and it is hard to know if their speculations are valid.
I have only suggested revision. The initial library randomized 5 positions within the PAM interacting domain (PID) of the protein and lead to a variant, KG, with the altered specificity they desired. They then did a further randomization using error-prone PCR to obtain additional variants, leading to the VRKG that they analyzed in more detail. I don't think they ever defined what the PID region is (what amino acid position it covers) and I was at first surprised to see that the new variants were more than 200 aa away from the first ones. It would have helped my understand that section if they had explicitly stated what region they were mutagenizing. The Methods section provides a bit more detail, but I think that information is still missing.

Reviewer #2:
Remarks to the Author:
In this manuscript, Goldberg, et. al. describe a dual negative/positive selection strategy in order to identify mutations within the PID of S. pyogenes Cas9 which utilize the non-canonical NAG PAM recognition sequence and minimize the acceptance of the wild-type NGG PAM sequence motif. After two rounds of directed evolution with their dual selection platform, the authors highlight a quadruple mutant, VRKG, which enables higher tolerance of NAG PAMs, but still retains some low-level wild-type NGG tolerance that further counter selection could not resolve. The work demonstrates the first development and application of a dual selection approach to access productive S. py. Cas9 PAM variants.

Overall:
This manuscript was well thought out and followed a logical flow, with a focus on enabling directed evolution of Cas9 PAM acceptance. I appreciated the care that was taken to develop and validate the mechanics of the selection strategy. The discussion was particularly engaging and illuminating

of the mechanism of altered substrate tolerance.

Major concern:

Whilst the authors did utilize a dual selection applied to the PID of S. pyogenes Cas9 for the first time, my major concern was that it wasn't clear to the reader that the authors accessed/identified a superior Cas9 variant relative to what has been already identified in the field through directed evolution and engineering without counter-selection (Miller et al, Nature Biotechnology, 2020) or step-wise positive selection followed by negative selection as described in reference 7.

To be convincing that this dual selection strategy is advancing Cas9 directed evolution approaches, the authors need to do a head-to-head comparison of the NAG variants identified in previous publications, with the variant identified here, in human cells. To take it a step further, how has the counterselection effected the off-target profile of the engineered NAG-PAM nuclease? If there is still weak NGG activity and broader PAM sequence acceptance, has that impacted guide-independent, or guide-dependent nuclease activity? Have the authors selected for something that has higher substrate tolerance or shifted specificity (away from NGG and towards NAG)? The motivation for using a dual selection strategy, inclusive of a negative counter-selection, is (or I believe it to be) to maintain high specificity but to modulate on target activity. To support that aim, more robust characterization, in human cells is needed.

Can more rounds of evolution be conducted to fully realize the goal? How can the stringency of the selection be modulated to increase pressure in subsequent rounds of directed evolution?

Minor concerns:

what was the motivation for going after an NAG PAM. Since WT Cas9 already has activity on NAG, can you also pursue NCC PAMs for which there is no engineered Cas9 variant in existence?

Line 9 - I am not sure how to interpret "commensurate" in this sentence

Line 14 - the abstract is missing what the deliverable variant is out of the selection and some quantifying metrics. An example sentence would be along the lines of: "From our dual selection campaign, we report an XXXX variant of Cas9 which yields a xx-% (increase?) on the non-canonical NAG site relative to previously published variants and has a off-target profile of .... in human cells. This can only be included if the apples-to-apples comparison has been made and the variants identified from the screen are indeed superior to what has been characterized in the field previously.

Line 26 - should include reference: "Miller, et. al. Continuous evolution of SpCas9 variants compatible with non-G PAMs, Nature Biotechnol., 2020"

Line 66 - clarify a little how the omega subunit is contributing to the selection strategy. It may not be obvious to the reader that it is a subunit of RNA polymerase that non-covalently associated and can be used to recruit the polymerase. Question: why was a rpoZ KO strain not used? How does the w-dCas9 fusion outcompete endogenous omega subunit? maybe include a sentence here that explains why a knock out wasn't used.

Line 84 and 86, these lines could use a little bit of clarity, what was activated? Does this mean to say RNA polymerase hybridization with the omega subunit?

Line 113 - define NNS-randomized

Line 126 - how many colonies were sampled?

Line 181 - why were so few colonies sequenced? How did you confirm you adequately sampled all clones to fully capture all surviving variants?

Line 255 - "inefficient plating" is a bit confusing. Maybe depletion of colonies might be more clear? I was also confused by the term "Select" in this line as the plasmids have maintenance markers which antibiotic would be used to select for these plasmids. It may be helpful to say "maintenance plus challenge antibiotic"

Line 268 - For me, this section seemed to be a distraction and it may be more suitable for the SI or a note? In the end, the Zn finger approach didn't yield any productive improvements to your Cas9 variant.

Line 342 - avoid the use of "novel"

Authors' Response to Reviewer Comments (in light blue text)

Reviewer #1 (Remarks to the Author):

This paper describes two novel techniques for doing simultaneous positive and negative selection for proteins with desired DNA-binding specificity. One method is performed in bacteria and is an extension of the bacterial one-hybrid method for which expression of an essential is under both positive and negative control. The other method utilizes yeast and selects for functional cleavage activity of the variant Cas9 proteins, again both positively and negatively. Current approaches use sequential, rather than simultaneous, positive and negative selections and the authors point out that there are some advantages, besides just being faster. The two different systems generally give the same results, but there may be advantages to one or the other for specific problems. They utilize these methods to obtain novel variants of the Cas9 protein that alters the PAM specificity, now preferring an NAG sequence while discriminating against the normal NGG sequence. Additional rounds of mutation and selection identified a 4 amino acid variant, VRKG, with increased specificity for NAG over YGG sites but with a surprising affinity for RNG sequences. They follow this up with additional experiments and computational modeling to try to explain the observed preferences, which is partially successful. They also try adding a zinc finger domain to the Cas9 protein because it increases the specificity in vivo for sites that also contain the ZF binding site. However this did not lead to improvements in the dual selection strategy and was not pursued further.
The two strategies presented in this paper are clever ways to do both and negative selection simultaneously. Besides speeding up the process this provides a different selection pressure that may lead to a different exploration of the sequence/function space and may lead to different solutions than the standard sequential selection methods. They didn't do a comparison so we don't know if that is true.
We are very grateful to the reviewer for the critical reading of the manuscript and positive comments. Indeed, we did not directly compare our simultaneous dual-selection scheme to a sequential positive-followed-by-negative selection scheme, but the comparisons that we provided between positive selection alone and simultaneous dual-selection (covered in Figs. 1, 2, and 3, along with Supplementary Figure S3 and Supplementary Tables S1, S3, S4, S5, S6) offer some relevant insight into how the sequence/function space was explored in our experiments. Negative selection was intrinsically coupled to positive selection in our dual-selection ω-dCas9 system, and that precluded us from directly testing the sequential scenario in *E. coli*. Sequential selections are presumably more feasible with our *S. cerevisiae* system but were not tested. As such, no claims were made in this work regarding whether simultaneous dual-selection strategies offer an advantage over sequential positive-negative selection strategies. However, it seems a reasonable assumption to us that simultaneous selection is the more robust approach in practice, even though the two strategies should provide similar outcomes in principle.

I think the method will be useful for people doing selections for DNA binding proteins with altered specificity, not just Cas9 variants. The paper is well written and contains

several additional and interesting analyses that I didn't describe above. The modeling part is a bit hand-wavy and it is hard to know if their speculations are valid.
I have only suggested revision. The initial library randomized 5 positions within the PAM interacting domain (PID) of the protein and lead to a variant, KG, with the altered specificity they desired. They then did a further randomization using error-prone PCR to obtain additional variants, leading to the VRKG that they analyzed in more detail. I don't think they ever defined what the PID region is (what amino acid position it covers) and I was at first surprised to see that the new variants were more than 200 aa away from the first ones. It would have helped my understand that section if they had explicitly stated what region they were mutagenizing. The Methods section provides a bit more detail, but I think that information is still missing.
We thank the reviewer for bringing this to our attention. In the revised manuscript, we have now stated explicitly within the main text which sequences were covered using our random mutagenesis schemes in round 2 and round 3.

Lines 236-239:

> “We generated two additional ω-dCas9 libraries wherein error-prone PCR was used to randomly mutagenize a region within the KG variant’s PID (or within Wt Cas9’s PID for comparison) spanning codons 1101-1342 and part of codon 1100.”

Lines 300-304:

> “Having established a system with improved counterselection stringency, we followed up with a library selection experiment where we randomly mutagenized the VRKG variant within its PID region using error-prone PCR (effectively mutagenizing residues 1102-1342) and assembled these insert fragments into the Cas9-Zif268 backbone upon delivery.”

Reviewer #2 (Remarks to the Author):

In this manuscript, Goldberg, et. al. describe a dual negative/positive selection strategy in order to identify mutations within the PID of S. pyogenes Cas9 which utilize the non-canonical NAG PAM recognition sequence and minimize the acceptance of the wild-type NGG PAM sequence motif. After two rounds of directed evolution with their dual selection platform, the authors highlight a quadruple mutant, VRKG, which enables higher tolerance of NAG PAMs, but still retains some low-level wild-type NGG tolerance that further counter selection could not resolve. The work demonstrates the first development and application of a dual selection approach to access productive S. py. Cas9 PAM variants.

Overall:
This manuscript was well thought out and followed a logical flow, with a focus on enabling directed evolution of Cas9 PAM acceptance. I appreciated the care that was taken to develop and validate the mechanics of the selection strategy. The discussion was particularly engaging and illuminating of the mechanism of altered substrate

tolerance.
We are very grateful to the reviewer for their thorough reading of the manuscript and their overall encouraging, constructive critiques.

Major concern:

Whilst the authors did utilize a dual selection applied to the PID of S. pyogenes Cas9 for the first time, my major concern was that it wasn't clear to the reader that the authors accessed/identified a superior Cas9 variant relative to what has been already identified in the field through directed evolution and engineering without counter-selection (Miller et al, Nature Biotechnology, 2020) or step-wise positive selection followed by negative selection as described in reference 7.
Regarding the Kleinstiver et al., 2015 work (reference 7), it should be clarified that while those authors did indeed use positive selection to evolve PAM variants from Cas9 mutant libraries, the subsequent ‘negative selection’ system they employed was with a **randomized PAM library** (for the purpose of characterizing the PAM repertoire of *particular* Cas9 variants that had already been evolved); **they did not employ any counterselection system with libraries of Cas9 mutants when evolving their PAM variants**. It should also be emphasized that the Kleinstiver et al., 2015 work identified a potent NGA(G)-binding variant, as we explained in our main text, but not an NAG-binding variant.

To be convincing that this dual selection strategy is advancing Cas9 directed evolution approaches, the authors need to do a head-to-head comparison of the NAG variants identified in previous publications, with the variant identified here, in human cells.
We previously provided a head-to-head comparison between Wt and VRKG in human cells on two NGG and four NAG PAMs, using a published EGFP-disruption assay. In parallel, the previously engineered VQR and SpCas9-NG variants were also tested on the same NGG targets and two NGA targets, collectively confirming the superior potency of Wt, VQR, and SpCas9-NG on their respective preferred PAMs. However, we found that the VRKG variant generally performed at least as well as Wt on NAG, with significant or near-significant improvements for 3 of the 4 NAG targets tested. The 4th target has a NAGG PAM, and the observation that VRKG had no advantage on this NAGG PAM is plausibly related to Wt SpCas9’s slight preference for a ‘G’ at position 4 of the PAM (Fig. 4a and [PMIDs: 23360965, 26098369, and 32832642]). **We have now expanded these efforts by repeating the head-to-head assay for Wt and VRKG (using the same four NAG and two NGG guides we had previously tested), this time alongside additional variants that are expected to target NAG and NGG PAMs, given their reported NNG or NR(R) PAM recognition**. The SpCas9-NG (VRVRFRR) variant was included in this batch of experiments because it has also been reported to display some activity on NA PAMs (in addition to NG PAMs). Note that NAG targets can indeed also be accessed by variants that recognize NG PAMs if the guide is shifted 1 bp; we did not test NG variants with shifted guides because ideal head-to-head comparisons must utilize identical guides (given that guide-dependent effects impact

absolute targeting efficiencies [PMIDs: 30166441, 26780180, 31937939]), and because precise guide placement can be critical for controlling the editing window in base editor applications. Given the PAM repertoires reported for each of the previously published variants, our results were generally consistent with the literature. Our measurements with Wt SpCas9 also agreed quite closely with values reported in the Kleinstiver et al., 2015 work from which we adapted this assay. In addition, these results help to highlight VRKG's evolved preference for NAG PAMs over YGG PAMs, as VRKG was the only variant tested here that provided less than 20% knockdown efficiency with both of the NGG sgRNAs.

To take it a step further, how has the counterselection effected the off-target profile of the engineered NAG-PAM nuclease? If there is still weak NGG activity and broader PAM sequence acceptance, has that impacted guide-independent, or guide-dependent nuclease activity?
It should be clarified that we did not detect broader PAM acceptance relative to Wt when considering all 5 bp PAM possibilities in our PAM depletion dataset (see below in response to the next comment)**.**

We should also point out that all of our mutations are confined to Cas9's PID, and this domain is not directly involved in the regulation of Cas9's mismatch fidelity (e.g., in the same way that the REC3 domain is). Nevertheless, we agree with the reviewer that off-target profiling in human cells offers additional insight into the PAM preference and overall specificity of Cas9 variants. Therefore, we included some GUIDE-seq data in the revised manuscript. PAMs that conform to NAG or RNG were clearly abundant in the top off-target hits for VRKG (Supplementary Fig. S12), corroborating our other data throughout the work. The lower—albeit somewhat similar—number of total off-targets detected with VRKG compared to Wt (Fig. 5b) plausibly reflects VRKG's lower overall activity, rather than differences in its PAM permissiveness. In fact, additional analyses of our PAM-depletion dataset are now provided in the revised manuscript as explained below, and these analyses clearly show that VRKG's PAM repertoire is similar to Wt's in breadth, but its overall activity is lower (Supplementary Fig. S10). The ~ 9-fold higher on-target activity measured for VRKG compared to Wt Cas9 in this experiment (Fig. 5c) is likely also related to Wt Cas9's higher overall activity (as we expect higher activity to result in more cleavage at off-target sites, even if equivalent specificities are assumed). Future work could examine whether truncated sgRNAs [PMIDs: 24463574, 25513782] or known high-fidelity mutations outside of the PID [PMIDs: 26735016, 26628643, 29431739, 30082871, 30082838] could be combined with VRKG to further reduce its off-target activity.

Have the authors selected for something that has higher substrate tolerance or shifted specificity (away from NGG and towards NAG)?
This is a central question in our work, and again we want to thank the reviewer for sharing their critical perspective and prompting further analyses on our part. We did aim to answer this initially with our comprehensive PAM-depletion experiment and

subsequent modeling analyses (Figs. 4, 6 and Supplementary Figs. 9, 14). From those experiments and analyses it appears that VRKG possesses on the one hand shifted specificity away from NGG (in particular YGG) and NGA PAMs, and on the other hand, higher substrate tolerance (recognizing 'RNG' PAMs in addition to NAG, although NAG PAMs appear to be recognized most strongly). To more quantitatively compare the global PAM tolerance of our variants with that of wild type, we re-analyzed our PAM-depletion dataset to determine the number of unique five-bp PAMs depleted by each variant (out of the 1024 possibilities), with each spacer. We found a striking similarity in the total number of unique PAMs depleted by our variants (Supplementary Fig. S10a)—despite their shifted preferences—and therefore conclude that the specificity of these variants was principally *altered* rather than *broadened*. Given that the maximal activity observed with wild type on its preferred PAMs (NGG) was greater than that of our evolved variants on their preferred PAMs (NAG) in various assays, we also compared total depletion activities across the entire five-bp PAM repertoires by summing the fold-depletion values measured with each variant and each spacer (Supplementary Fig. S10b). This latter analysis corroborated our results from other assays, confirming that Wt Cas9 offers greater targeting potential than the KG and VRKG variants when considering their entire five-bp PAM repertoires.

The motivation for using a dual selection strategy, inclusive of a negative counter-selection, is (or I believe it to be) to maintain high specificity but to modulate on target activity. To support that aim, more robust characterization, in human cells is needed.
There are, evidently, many ways to navigate Cas9's sequence space and functional landscape. We have highlighted simultaneous positive/negative selection in this work, but various caveats of the approach were clear to us, and we made an effort to present them transparently to the readership (e.g., by including parallel experiments using positive selection alone in each round, and also by discussing works from other groups that successfully employed positive selection alone). We believe that robust characterization of Cas9 variants and their PAM preferences can in fact be achieved through various means, given appropriate controls and eventual benchmarking in human cell culture assays (as the latter have indeed become standard). Toward this end, we've added additional head-to-head comparisons with relevant engineered and wild type variants in human cells, as mentioned above. In addition, a GUIDE-seq comparison was performed in human cells using a published sgRNA: the 'HEK site 4' sgRNA previously tested with Wt SpCas9 in the HEK293 and U2OS backgrounds [PMIDs: 25513782 and 32042170]. Results from these experiments are included in a main figure item (the new Figure 5) to ensure their visibility in the work.

Can more rounds of evolution be conducted to fully realize the goal? How can the stringency of the selection be modulated to increase pressure in subsequent rounds of directed evolution?
We did attempt a 3rd round of directed evolution using a modulated version of our yeast system where negative selection stringency was specifically increased through the fusion of compensatory zinc fingers, but we were unsuccessful in detecting further

evolution via random mutagenesis in the PID region (Supplementary Fig. S7 and Table S6). At least in these experiments, we do not believe that a lack of stringency was limiting our efforts *per se*, for the following reasons. Regarding positive selection, we found that Wt Cas9 can achieve almost 10-fold higher cutting efficiency on an NGG PAM at the *LYS2* locus when compared to the absolute cutting efficiency of the KG or VRKG variants on the same PAM (Supplementary Fig. S7a; see also Supplementary Fig. S2a for Wt vs. KG on an NAG PAM), implying that positive selection was not yet saturated and that there is still room for an 'upward climb'. Note also that the KG/VRKG variants' NAG activity with the Cas9 backbone lacking zinc fingers falls into the same regime as their NGG activity in this assay, as shown by comparisons between panels 'a' and 'b' in Supplementary Fig. S7 and with Supplementary Fig. S2a. Regarding negative selection, Supplementary Fig. S7b clearly demonstrates that we've increased selection stringency against the KG and VRKG variants, because virtually background levels of colonies are forming when clonal inserts are co-transformed with our Cas9-Zif268 backbone.

Nevertheless, we also considered the possibility that our negative selection stringency in this Cas9-Zif268 context was *too strong* to allow co-selection for variants that maintain robust NAG-targeting activity (given that NAG and NGG PAMs are so similar). In other words, we may have imposed a bottleneck on the population. We were able to specifically tune this counterselection stringency by supplying weaker binding sites for the Zif268 zinc fingers, based on a published dataset with *in vitro* affinity measurements [PMID: 23012409]. Using this strategy we resolved at least one additional negative stringency tier whereby only the Wt and KG variants—but not the mKG-d4 variant carrying VRKG substitutions—were robustly counterselected upon assembly with a Cas9-Zif268 backbone (see below for the 1.76e-08 and 1.74e-08 K_d sites). Thus, in principle it appears that we could have re-isolated the VRKG substitutions by mutagenizing the KG PID for screening in these modified yeast strains.

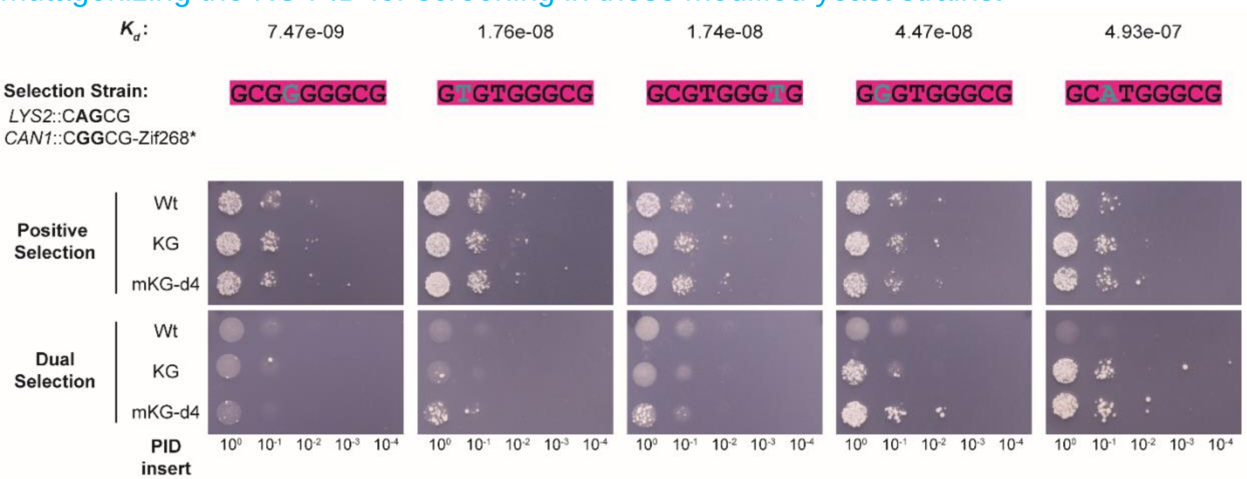

Lower-affinity binding sites for Zif268 offer reduced counterselection stringency in yeast selection experiments with a Cas9-Zif268 backbone. Testing of the Wt, KG, and mKG-d4 PIDs in a dual-selection strain configured with a CAG PAM at the *LYS2* locus and a CGG PAM with one of several modified Zif268 binding sites (Zif268*) at the *CAN1* locus. Sequence deviations from the maximal-affinity Zif268 consensus binding site are colored in teal lettering. Cas9-Zif268 backbone was co-delivered with a molar

excess of clonal PID inserts, and cells were plated in parallel as 10-fold serial dilutions on positive-selection plates and dual-selection plates as indicated (as well as control plates to determine total transformants; data not shown).

We also believe we identified another negative stringency tier using the 7.47e-09 K_d site, whereby the VRKG variant was counterselected to near-background levels, but not below (as observed with the original Zif268 consensus binding site). We attempted to use this strain for re-screening our round 3 randomly mutagenized PID library. Unfortunately, although additional dual-selection isolates were recovered this time around, mutational convergence was still not detected (data not shown).

In summary, given that we have apparently not exhausted our selection stringency in the yeast system, it is conceivable that further rounds of evolution could indeed be successfully conducted. However, we speculate that this would likely require alternative site-saturation mutagenesis schemes for the PID, or mutagenesis of other regions in the Cas9 protein.

Minor concerns:

what was the motivation for going after an NAG PAM. Since WT Cas9 already has activity on NAG, can you also pursue NCC PAMs for which there is no engineered Cas9 variant in existence?
Early work from other groups had clearly shown that NAG PAMs are the most strongly recognized non-NGG PAMs for Wt SpCas9 [PMIDs: 23360965, 26098369], and this is in fact the primary reason we pursued NAG; we surmised that NAG substrates offer the closest mimic to NGG, and evolving preference for NAG over NGG might therefore be difficult without the aid of negative selection. Furthermore, when this work began, we were not aware of any SpCas9 variants that were engineered to lose NGG activity while maintaining commensurate *or* improved activity on NAG PAMs. Our aim was to evolve variants with altered, rather than broadened specificity, with potential utility in allele-specific editing applications whereby single-nucleotide discrimination between NAG and NGG sequences could be achieved at the PAM-recognition step. A mention of allele-specific editing was added to the introduction, along with some references (Lines 31-34). In line with this aim, we successfully evolved variants whose specificity was shifted towards NAG recognition and away from NGG recognition. However, given the lower maximal activity that we measured for the KG and VRKG variants in our experiments relative to Wt on their respective preferred PAMs, including in our human cell-culture experiments, it should be clear to the reader that this aim was not fully realized.

Regarding NCC recognition, we've included B1H control experiments to further justify our use of GCC as a null counterselection PAM in bacteria (validating the notion that we could effectively achieve 'positive selection alone' with isogenic reporter plasmids that harbored NCC instead of NGG at the downstream counterselection site). In these control experiments, we attempt to detect activity on NCC by co-delivering ω-dCas9 libraries (or a Wt ω-dCas9 control plasmid with non-targeting sgRNA) and reporter

plasmids that harbored only the NCC PAM at the upstream activation site, and then plated with 2mM 3-AT for low-stringency selection. From these selection experiments it was apparent that our libraries did not offer detectable NCC activity above background, in stark contrast to what was observed when the NGG PAM was provided (Supplementary Fig. S13).

In parallel, we plated additional cells from the NCC-selected pools on larger plates (1e8 double transformants for each of the non-targeting control and randomly mutagenized library pools, corresponding to ~10-fold and ~3-fold oversampling, respectively, of the theoretical double-substitution diversity within the mWt and mKG libraries; and, 4e8 double transformants for the initial saturation library with 1332-1336 NNS-randomized, corresponding to ~6-fold oversampling of the estimated actual library size). After 44h at 37 C, we Sanger-sequenced the PID region from 30 colonies of the saturation library pool, and from 24 colonies of each of the random mutagenesis pools, but could not discern enrichment for any residues (data not shown). Therefore, it appears that NCC-binding variants do not exist in our libraries and the evolution of such variants, if possible, would require alternative randomization schemes. For example, one might focus on minor groove contacts or 'second shell' residues that influence how residues 1332-1336 engage the DNA.

Line 9 - I am not sure how to interpret "commensurate" in this sentence
We clarified that sentence by adding the phrase "relative to wild type". It now reads as follows:

Lines 7-10:

> "In this work, we exploit *in vivo* directed evolution systems that incorporate simultaneous positive and negative selection to evolve SpCas9 variants with commensurate or improved activity on NAG PAMs **relative to wild type** and reduced activity on NGG PAMs, particularly YGG PAMs."

In that sentence, "commensurate" activity is meant to imply *equivalent* activity; e.g., as measured with the NAG-4 sgRNA in EGFP knockdown assays, where no significant difference was discernible relative to wild type (Fig. 5a and Supplementary Fig. S11a). In other cases, e.g., with the NAG-1 and NAG-2 sgRNAs, a statistically significant *improvement* was measured (Fig. 5a and Supplementary Fig. S11a). These results were fully consistent with results from our other experiments throughout the work, and we therefore believe inclusion of this claim in the abstract is well-justified.

Line 14 - the abstract is missing what the deliverable variant is out of the selection and some quantifying metrics. An example sentence would be along the lines of: "From our dual selection campaign, we report an XXXX variant of Cas9 which yields a xx-% (increase?) on the non-canonical NAG site relative to previously published variants and has a off-target profile of .... in human cells. This can only be included if the apples-to-apples comparison has been made and the variants identified from the screen are

indeed superior to what has been characterized in the field previously.

In the abstract (as mentioned above) and throughout most of this work, our claims were focused on comparisons with the Wt SpCas9 protein. We believe this was appropriate for at least two reasons. First, our variants were evolved from Wt SpCas9 in two sequential rounds. Second, the other published NAG-targeting variants were engineered for broadened specificity, rather than altered specificity. Therefore, the other engineered variants are not expected to display strongly reduced activity on NGG PAMs, and indeed, results from our head-to-head EGFP knockdown experiments in human cells suggest that only VRKG will offer reliably lower activity on YGG PAMs among the variants tested. The fact that certain sgRNAs produced lower NAG-targeting activity with our VRKG variant than with the SpCas9-NRRH and SpRY variants in EGFP knockdown assays is important to note—and indeed was noted in the results section. Having said that, this result was also not surprising given what is established about negative selection: in some cases it can constrain improvements in on-target activity (perhaps especially in cases where the on- and off-target substrates are closely related, as for NAG and NGG PAMs, respectively). We allude to these caveats in the introduction and elaborate more thoroughly on them in the discussion.

It was our intention to remain within the journal's strict 150-word limit for Abstract sections. If the editor feels that an expanded Abstract is necessary, we could provide one in a later revision.

Line 26 - should include reference: "Miller, et. al. Continuous evolution of SpCas9 variants compatible with non-G PAMs, Nature Biotechnol., 2020"

We thank the reviewer for bringing this impressive work to our attention. We now included the citation in that sentence of the introduction as suggested. Furthermore, references were updated throughout the manuscript to include additional citations to that work and other relevant recent publications, where appropriate.

Line 66 - clarify a little how the omega subunit is contributing to the selection strategy. It may not be obvious to the reader that it is a subunit of RNA polymerase that non-covalently associated and can be used to recruit the polymerase. Question: why was a rpoZ KO strain not used? How does the w-dCas9 fusion outcompete endogenous omega subunit? maybe include a sentence here that explains why a knock out wasn't used.

Line 84 and 86, these lines could use a little bit of clarity, what was activated? Does this mean to say RNA polymerase hybridization with the omega subunit?

To be clear, the *E. coli* selection strain we used in this work is indeed effectively *rpoZ*$^{-}$, as it harbors a chromosomal zeocin resistance marker insertion in place of the endogenous *rpoZ* coding sequence. This strain was established in previous works that are referenced in the first paragraph of Results (as well as in Methods, where a full genotype was also provided).

Regarding RNA polymerase recruitment, our usage of the term “activation” in the context of those sentences can be understood to imply *transcriptional* activation. To be more explicit about that, we edited one of the sentences to read “ω-dependent **transcriptional** activation of a plasmid-borne *HIS3* marker” (Lines 69-70). Generally speaking, we believe “transcriptional activation” is unambiguously understood to imply processes whereby the recruitment of RNA polymerase to a promoter is upregulated (resulting in elevated transcription of the downstream locus). Because our B1H system is in turn engineered to depend on ω-fused proteins for transcriptional activation (above background), as we also explained in that paragraph, this was referred to as “ω-dependent transcriptional activation”.

For these reasons, we likewise edited the first sentence of the second paragraph to include “transcriptional” as a qualifier for “activation”. It now reads as follows:

> “Previous reports demonstrated that ω-dCas9 (or dCas9-ω) **transcriptional** activation requires precise target site positioning upstream of promoters[15,35], but prescriptive guidelines for ensuring optimal activation were proposed only recently[37].” (Lines 88-91)

If the reviewer feels that additional clarifications are still needed, we would be happy to add them in a later revision.

Line 113 - define NNS-randomized
We’ve adjusted the text in line with this reviewer’s suggestion. It now reads:

> “The initial ω-dCas9 library we constructed and screened was NNS-randomized (where ‘N’ represents any of the four DNA bases and ‘S’ represents ‘C’ or ‘G’) at five codons (1332-1336) of the PAM-interacting domain (PID), including the R1333 and R1335 residues that are critical for Cas9’s activity on NGG PAMs[39]. NNS-randomized triplets offer at least one codon for each of the 20 standard amino acids and one stop codon.” (Lines 117-123)

Line 126 - how many colonies were sampled?
The results summarized in Supplementary Table S1 are from experiments where 24 colonies were sampled for each condition. Typically, 24-32 colonies from each selection experiment in this work were picked and used for seeding colony PCRs, which were then sent for Sanger sequencing directly (otherwise, overnight cultures were launched for miniprepping and then sequenced). Unless noted otherwise in the text (namely, for the dual selection experiment of Supplementary Table S6), we obtained an excess of colonies on selection plates compared to the number of colonies we picked for sequencing. In some cases, all of the colonies we sampled for a given condition produced sequences that could be unambiguously translated; in other cases, a few of the isolates either produced no trace or an ambiguous trace. Given that colony PCR was typically employed, missing or incomplete sequences could have arisen from failed PCRs (or otherwise, processing errors at the sequencing facility). When isolates from

our experiments were not saved, re-amplification/re-sequencing attempts were not an option. Hence, sequences from isolates that could not be unambiguously translated were excluded from the tables and WebLogo analyses in this work (which is also why isolate numbers are discontinuous in some of the tables, when they were listed). The “Data exclusions” box in the reporting summary document was updated to reflect this.

The final number of sequences used for each WebLogo (in Figs. 1 and 2) was always indicated in the figures or their legends, and all of the *complete* PID protein sequences we recovered in each experiment were summarized in their respective Supplementary Tables. For example, in Supplementary Table S1, all non-synonymous substitutions we identified among the complete protein sequences are summarized in accordance with the footnote description (residues not listed or written in black text were unsubstituted relative to wild type). The only exception was in Supplementary Table S3, where the fraction of non-Wt sequences out of total sequences recovered was indicated above the table, but the Wt sequences were excluded to reduce clutter (see our response to the next comment below for more details about that experiment).

Line 181 - why were so few colonies sequenced? How did you confirm you adequately sampled all clones to fully capture all surviving variants?
In fact, we sampled more than just 14 colonies and 19 colonies in that experiment. As noted in the legend, that was just the final number of non-Wt sequences recovered. With positive selection alone, 60 colonies were checked and we obtained 58 sequences (of which 14 were non-Wt – Table S3). With dual selection, 24 colonies were checked and we obtained 19 sequences (of which all 19 were non-Wt – Table S3). More colonies were checked with positive selection alone because we anticipated recovering a surplus of parental Wt carryover, based on our initial colony-picking experiments with the ω-dCas9 library from which that yeast library was derived. **In both cases, the number of colonies recovered on selection plates was in excess of the number of colonies that we checked** (672 colonies with positive selection and 60 colonies with dual selection), so we know that our library screening depth was adequate, although we did not deep sequence any post-selection pools of yeast. Indeed, the frequency of parental Wt recovered with positive selection alone (44/58) roughly mirrored what we recovered in our B1H deep sequencing experiments—which were plated in 6-fold excess of the library size and in biological duplicate (as explained in the Methods). Importantly, as we noted in the Fig. 2c legend, recoded Wt isolates were not detected in either of these experiments. Moreover, given the convergence on the KG variant observed among the sequences we *did* recover (particularly in the yeast dual-selection experiment), as well as its relative abundance in the bacterial deep sequencing pools, we believe our sampling efforts were acceptable. In the Methods section we estimate the effective library sizes assembled in yeast under positive- or dual-selection conditions; these estimates were back-calculated based on the concentration of total transformants measured for each transformation mixture and the volume plated under each condition. From these calculations it is clear that the effective library sizes we assembled in yeast were smaller than the theoretical complexity those libraries could encode. As such, we avoided claims about oversampling in yeast.

Line 255 - "inefficient plating" is a bit confusing. Maybe depletion of colonies might be more clear? I was also confused by the term "Select" in this line as the plasmids have maintenance markers which antibiotic would be used to select for these plasmids. It may be helpful to say "maintenance plus challenge antibiotic"
We were operating under an assumption that 'plating efficiency' is generally understood as the efficiency of colony or plaque formation; thus, "inefficient plating" would imply a reduction in the number of colonies formed. We should have been more clear, and in line with this reviewer's suggestions we've updated the sentence to be more explicit:

> "When single-target reporter and Cas9 plasmids are co-delivered, reduced colony formation on media with antibiotics that select for maintenance of both plasmids is indicative of target plasmid cleavage." (Lines 264-266)

Line 268 - For me, this section seemed to be a distraction and it may be more suitable for the SI or a note? In the end, the Zn finger approach didn't yield any productive improvements to your Cas9 variant.
As explained in response to a major comment above, our failure to achieve improvements in round 3 was apparently due to limitations of the PID random mutagenesis strategy we employed, and not the selection system itself. Given that engineered dual-selection systems are the focus of the paper we felt it appropriate to highlight this novel scheme for counterselection stringency modulation, and we believe this strategy could prove useful for navigating other evolutionary landscapes (especially given that it is likely to be applicable to the engineering of DNA binding proteins in general). The relevant Figure and Table items referenced in this section remain within the SI file, and we do not believe relocating the entire section to a Supplementary Note should be necessary. If the editor feels strongly that inclusion of that section in the main text is unjustified, we could move it to the supplement in a later revision.

Line 342 - avoid the use of "novel"
Removed from the sentence.

Reviewers' Comments:

Reviewer #1:
Remarks to the Author:
I think the authors have adequately addressed all of the concerns raised in the previous review. I have no more suggested revisions.

Reviewer #2:
Remarks to the Author:
Thank you to the authors for clearly and kindly addressing my concerns and questions. I am satisfied with their responses and amendments to the manuscript.

Authors’ Response to Reviewer Comments (in light blue text)

Reviewer #1 (Remarks to the Author):

I think the authors have adequately addressed all of the concerns raised in the previous review. I have no more suggested revisions.
We again thank this reviewer for his or her critical reading of the manuscript and overall positive commentary.

Reviewer #2 (Remarks to the Author):

Thank you to the authors for clearly and kindly addressing my concerns and questions. I am satisfied with their responses and amendments to the manuscript.
We are glad to hear that this reviewer’s concerns were clearly addressed with our revisions, and grateful for his or her insightful critiques and commentary.
